# 3D In Vitro Platform for Cell and Explant Culture in Liquid-like Solids

**DOI:** 10.3390/cells11060967

**Published:** 2022-03-11

**Authors:** Duy T. Nguyen, Jack E. Famiglietti, Ryan A. Smolchek, Zadia Dupee, Nickolas Diodati, Diego I. Pedro, Juan M. Urueña, Matthew A. Schaller, W. Gregory Sawyer

**Affiliations:** 1 Department of Mechanical and Aerospace Engineering, University of Florida, Gainesville, FL 32611, USA; nguyenduy2308@ufl.edu (D.T.N.); jackfamiglietti@ufl.edu (J.E.F.); rsmolchek@ufl.edu (R.A.S.); ndiodati@dental.ufl.edu (N.D.); dpedro@ufl.edu (D.I.P.); jmuruena@ufl.edu (J.M.U.); 2 Division of Pulmonary, Critical Care, and Sleep Medicine, University of Florida, Gainesville, FL 32611, USA; zadia.dupee@medicine.ufl.edu (Z.D.); matthew.schaller@medicine.ufl.edu (M.A.S.)

**Keywords:** 3D cell culture, liquid-like solids, microgels, perfusion, in vitro, microexplants

## Abstract

Existing 3D cell models and technologies have offered tools to elevate cell culture to a more physiologically relevant dimension. One mechanism to maintain cells cultured in 3D is by means of perfusion. However, existing perfusion technologies for cell culture require complex electronic components, intricate tubing networks, or specific laboratory protocols for each application. We have developed a cell culture platform that simply employs a pump-free suction device to enable controlled perfusion of cell culture media through a bed of granular microgels and removal of cell-secreted metabolic waste. We demonstrated the versatile application of the platform by culturing single cells and keeping tissue microexplants viable for an extended period. The human cardiomyocyte AC16 cell line cultured in our platform revealed rapid cellular spheroid formation after 48 h and ~90% viability by day 7. Notably, we were able to culture gut microexplants for more than 2 weeks as demonstrated by immunofluorescent viability assay and prolonged contractility.

## 1. Introduction

The history of cell culture is resplendent in efforts to extend the long-term culture of primary cells and tissues. Existing models and techniques for tissue culture (e.g., organoids and patient-derived explants) aim to recapitulate an aspect of in vivo biology in greater detail than cells grown in a monolayer [1,2,3,4]. The high rate of failure in phase 1 clinical trials is often attributed to ambiguous drug response in cell lines cultured in a two-dimensional (2D) liquid-cover monolayer, a lack of autologous immune cells in models, and the inaccurate representation of inter-individual heterogeneity in oversimplified in vitro models [4,5,6]. Three-dimensional (3D) cell models have been shown to recapitulate the relevant in vivo conditions such as cell–cell interaction, migration, differentiation, and drug sensitivity [7,8]. Existing 3D in vitro models include spheroids [9], organoids [10], and patient-derived explants [2,11]. The ex vivo culture of tissue explants retains the greatest degree of complexity and preserves the cell types, the cellular architectures, the fibrotic stroma found in the host-derived primary organ, as well as immune cell subsets, extracellular matrix proteins, and intricate cellular structures [2]. Extended explant culture will provide a valuable platform for studies of pharmacology, immunology, and physiology, amongst others.

One major obstacle to overcome in all 3D in vitro models is waste metabolite removal [4,12,13,14,15]. In 2D liquid-cover culture and conventional 3D methods such as suspension culture [16,17], hanging drop [18], or agitation of media [13,14,19] the waste metabolites are rapidly diluted into the media via free convection, resulting in a complex milieu of signals that may alter cellular function [4,15]. Other existing three-dimensional (3D) culture platforms such as embedment culture in gels of collagen [20], decellularized allogeneic extracellular matrix [21,22], or Matrigel^TM^ [23] restrict convection and are limited to diffusive transport that results in steep metabolite gradients that create a localized accumulation and concentration of waste metabolites, resulting in increased toxicity within the explant microenvironment. In nature, the bulk transport of oxygen, nutrients, and metabolic waste occurs through capillary-based forced network perfusion from the vascular and lymphatic system that permeates every tissue in the body; this exquisite network enables the precise control of local concentrations. Many recent efforts in bioprinting and microfluidic technologies have focused on reproducing microvascular networks [24,25,26], which remain a grand challenge of biofabrication and tissue engineering. Perfusing cell culture media through monocultures or organoids composed of gut, liver, or kidney cells is another pioneering approach facilitating the growth of primary cells from tissues [27,28,29,30].

Microfluid platforms and 3D printing technologies have provided tools for cell culture and various in vitro assays [19,31,32]. This rapidly growing field of research has contributed valuable tools to investigate mechanistic studies that utilize the advantages of length scales below 100 µm to enable short reaction times, predictable laminar flows, and rapid thermal transport [33,34]. Microfluidic bioreactors and organ-on-chip platforms have been designed to culture cells in microchamber(s) through which media is perfused via networks of nano- or micro-channels. These microfluidic technologies are often fabricated based on certain well-known criteria and aim to recapitulate various aspects of in vivo conditions including physiologically relevant mechanical cues and controllable flow rates, while also providing optical access to facilitate in situ imaging capability. These microfluidic systems are increasingly facilitating the co-culture of different cell types and perfusion of small molecules, drugs, and growth factors, and have recently been applied successfully as a tool to test several therapeutic compounds on patient-derived cancer cells [19,35]. Indeed, the organ-on-chip models (e.g., lung-on-a-chip, vessels-on-a-chip, tumor-on-a-chip) have triggered numerous applications in studying alveolar–capillary interface models [36], flow-induced differentiation [37], tumor angiogenesis [38], and cancer cell extravasation [39]. These platforms represent significant advances over traditional cell culture methods but have not been widely applied to tissue explant models. The ex vivo culture of tissue explants is complicated by irregular geometries, significant tissue-level organization, and heterogeneity, and it has proven extremely difficult to maintain and balance media components to support the continuous culture of a heterogeneous population of cells that are present in these explants.

We have developed a 3D ex vivo culture platform that enables the controlled delivery of media and removal of waste metabolites by means of perfusion through a porous medium of granular microgels with interstitial spaces that are designed to recapitulate an in vivo capillary bed. Liquid-like solids (LLS) provide a soft granular microgel bed made of hydrogel particles that are approximately 100 µm in characteristic diameter [40] to act as both a 3D support medium and as a linear resistor, enabling controlled perfusion rates by varying the height of the LLS bed, the permeability, or both. We have demonstrated the advantages of this platform by culturing single cells and mouse intestinal microexplants ex vivo for more than 2 weeks, demonstrating functional readouts of cell viability and gut contractions that are lost at earlier time points in traditional 2D liquid-cover culture. These results indicate a potential for perfusion-enabled 3D culture to facilitate detailed in vitro investigation of tissue microexplants comprising a variety of physiologically relevant cell types and structures.

## 2. Materials and Methods

### 2.1. Manufacturing, Assembly, and Sterilization of Injection-Molded Perfusion Plates

The perfusion-enabled 3D culture plates comprise 4 injection molded polystyrene components, hydrophilic polycarbonate track-etched (PCTE) semi-permeable membrane (Qty: 1) (Sterlitech, Auburn, WA, USA, PCT2020030), and elastomer retaining bands (Qty: 2). The polystyrene components are manufactured free of pyrogens, enzymes, and other harmful contaminants. Preparation and assembly are completed in a sterile environment. The PCTE semi-permeable membrane is heat-sealed to the surface that bounds the outlets of the wells, selectively allowing passage of media and small molecules but retaining LLS and biological samples. The base component of the assembly that encloses the effluent collection compartments and sustains low vacuum is sealed using a biocompatible, light-curing acrylic adhesive (LOCTITE^®^, Henkel, Düsseldorf, Germany, AA 3926™) and 365 nm light source. Each assembly is then individually wrapped in Tyvek bags (Andersen Sterilizers, Inc., Haw River, NC, USA, Cat. AN880) and sterilized in an ethylene oxide (EO) chamber (Andersen Sterilizers, Haw River, NC, USA, AN74iX).

### 2.2. Human Cardiomyocyte AC16 Culture and Handling in LLS

AC16 human cardiomyocyte cell line was purchased from Millipore Sigma Cat. No. SCC109 (Burlington, MA, USA). The cell line was employed as an adherent single-cell model for these perfusion culture studies. Culture media for AC16 cells was prepared and filtered through a 0.2 µm bottle filter, following the protocol provided with the cells [41]. Prior to perfusion culture, the cells were cultured in a T75 cell culture flask in DMEM/F12 with L-Glutamine (Corning Inc., Corning, NY, USA, 10-092-15), 12.5% FBS (Fisher Scientific (Waltham, MA, USA)/Mediatech Inc. (Manassas, VA, USA), Cat MT35011CV, Heat inactivated), and 0.15% Primocin. For perfusion culture, AC16 cells at passage 2 were suspended in LLS equilibrated in media at a density of 10^6^ cells/mL, dispensed into each well (200 µL each) of the perfusion plate, and cultured at 37 °C, 5% CO_2_, and at 3 different flowrates (25.4 ± 4.1, 35.3 ± 4.1, and 43.1 ± 4.8 µL/hr/well) to investigate optimal culture conditions.

Cell dispersion in LLS was achieved by gently mixing cells into LLS, yielding a randomly dispersed cell density of 10^6^ cells/mL. The mixture of cells and LLS in liquid media was centrifuged at 100 g for 5 min with a moderate acceleration and deceleration setting of 5 (Thermo Fisher Scientific, Waltham, MA, USA, Sorvall ST 40R). After centrifugation, the LLS–cells mixture was collected at the bottom of the centrifuge tube. The supernatant was then aspirated, and the centrifuged LLS–cells mixture was gently mixed with wide-bore pipette tips to ensure uniform dispersion of cells. The mixture was then transferred to each well of a perfusion plate at 200 µL/well using wide-bore pipette tips. The mixture of cells and LLS was loaded into the individual wells (24) within a perfusion plate, and the perfusion plate assembly was then centrifuged at 100 g for 5 min at the same acceleration and deceleration setting of 5 (Thermo Fisher Scientific, Waltham, MA, USA, Sorvall ST 40R). A liquid culture medium was then slowly added to fill the media reservoirs. Perfusion was initiated, and the corresponding time was marked.

At 24 h intervals, the effluent was collected, and the volume from each quadrant was measured to record the daily flow rate for each culture condition. A small sample of effluent was collected each day and stored at −80 °C for metabolite analysis.

### 2.3. Glucose and Lactate Assays

Investigation of glucose and lactate concentrations in the effluent media was performed via bioluminescent detection assay kits (Promega GlucoseGlo, J6021, and Promega LactateGlo, J5021, Promega, Madison, WI, USA) and recorded using a plate-reading luminometer (BioTek Synergy HTX multimode microplate reader, Agilent, Canta Clara, CA, USA) following the manufacturer-provided protocols. In brief, daily effluent media, perfused through all wells containing AC16 cells (initial seeding density of 10^6^ cells/mL/well) from each culture condition, were collected. The effluent media were diluted in PBS (500-fold for glucose and 200-fold for lactate) to fit into the linear range of the assays according to the manufacturer’s instructions. The diluted samples and detection reagent solution, a mixture containing reductase, reductase substrate, glucose or lactate dehydrogenase, and NAD (provided by the manufacturer), were gently mixed at a 1:1 ratio by volume (50 µL of samples: 50 µL detection reagent solution) in a 96-well plate and incubated in the dark at room temperature for 60 min followed by luminescence recording using a plate-reading luminometer. A standard curve was used in parallel, as specified by the manufacturer, to determine the concentrations of glucose and lactate.

### 2.4. Mouse Colon Explant Culture

C57bl/6 mice (The Jackson Laboratory, Bar Harbor, ME, USA, strain #: 000664) were sacrificed following UF IACUC protocol 201810479. An approximately 10 cm segment of the large intestine from the cecum to the distal colon was harvested and placed in ice-bath cooled 1X PBS without calcium and magnesium. External membranes and fat were further removed using forceps and scissors. A syringe with an 18G needle was inserted into one end of the segment to flush the sample at least 3 times with ice-bath cooled PBS. The sample was then opened longitudinally using dissecting scissors and washed an additional 3 times with cold PBS. Holding one end of the sample with forceps, the intestinal segment was cut into small 1 mm pieces using a scalpel and transferred to a 2 mL microcentrifuge tube. The pieces were further cut with surgical scissors such that the characteristic size of each piece was less than 1 mm (with approximate tissue volume less than ~1uL). These microexplants were then transferred to a 15 mL tube containing ice-bath cooled PBS and further chilled on ice for at least 5 min. The supernatant was aspirated after observing a relatively clear collection of microexplants at the bottom of the tube. This aspiration procedure was repeated 3 times to remove residual single cells and extracellular matrix (ECM). The collected microexplants were separated into two groups for 2D and 3D perfusion culture using pre-wetted wide-bore micropipette tips.

For 2D experiments, the microtissue samples were directly cultured using a 96-well ultra-low attachment plate (Corning, Inc., Corning, NY, USA, 7007) covered with liquid media at 37 °C, 5% CO_2_, and in two different oxygen settings with partial pressures (PO_2_) = 20% and 90%. The liquid media used for all culture conditions (2D and 3D) were formulated with HEPES-buffered DMEM/F12 (Gibco, Billings, MT, USA, 11330032) supplemented with 10%FBS (Sigma-Aldrich, F4135), 100 U/mL penicillin and 100 µg/mL streptomycin (Gibco, 15140148), 50 µg/mL gentamycin (Sigma-Aldrich, St. Louis, MO, USA, G1914-250MG), 1:100 insulin/transferrin/selenite (Gibco, Billings, MT, USA, 41400045), 1 mg/mL AlbuMAX^TM^ (Gibco, Billings, MT, USA, 11020021), 14 nM glucagon (Sigma-Aldrich, St. Louis, MO, USA, G2044-5MG), 200 µM ascorbate-2-phosphate (Sigma-Aldrich, St. Louis, MO, USA, A28960-50MG), 50 ng/mL keratinocyte growth factor (Prospecbio, Ness-Ziona, Israel, CYT-721), 50 ng/mL mouse recombinant EGF (Gibco, Billings, MT, USA, PMG0844), 100 ng/mL mouse recombinant noggin (Peprotech, Cranbury, NJ, USA, 250-38-20UG), 1 µg/mL human recombinant R-spondin (Stemcell, Vancouver, BC, Canada, 78213), 10 nM [Leu15]-Gastrin I (Sigma-Aldrich, St. Louis, MO, USA, G9145), 500 nM A83-01 (Tocris, Bristol, UK, 2939), 10 mM nicotinamide (Sigma-Aldrich, St. Louis, MO, USA, N0636), and 10 µM SB202190 (Sigma-Aldrich, St. Louis, MO, USA, S7076). Media were changed every 24 h for all 2D cases.

For 3D perfusion culture, the microtissue samples were suspended in LLS equilibrated with liquid media. A small sample of this microtissue-LLS mixture was placed on an inverted light microscope to estimate the number of microexplants per unit volume. Each 3D culture condition used an individual perfusion plate, one for normoxic and another for hyperoxic conditions, with PO_2_ = 20% and 90%, respectively. Each well of a perfusion plate was then filled with a 200 µL volume of LLS containing up to 20 microexplants in suspension. Once filled, the plates were centrifuged to eliminate bubbles at 100 g for 5 min at room temperature with a moderate acceleration and deceleration setting of 5 (Thermo Fisher Scientific, Waltham, MA, USA, Sorvall ST 40R). Liquid medium was gently added into each quadrant of the plate. A 100cc Jackson-Pratt drain (Care Express, Cary, IL, USA, SU130-1305) was connected to induce the pressure gradient and initiate perfusion of the media. Identical culture conditions were maintained at 37 °C and 5% CO_2_, in two separate incubators with 2 different partial pressure settings, PO_2_ = 20% and 90%. The perfusion plates were cycled every 24 h by adding media to the media reservoirs and collecting effluent media from the waste collection compartments.

At specified time points during the experiment, random samples from each condition were observed and gut contractions were recorded using a Nikon inverted light microscope recording at an image collection frequency of 1 fps for 5 min. Samples from perfusion culture were transferred to a 96-well ultra-low attachment plate with pre-warmed media before video recording.

### 2.5. Microexplant Collection from LLS

To isolate microexplant samples from within the LLS suspension in each well, the liquid medium in each quadrant was completely removed. Then, using wide-bore 200 µL pipette tips, the microexplant–LLS mixture in each well was transferred dropwise into a 10 mL column of ice-bath chilled PBS + 10%FBS solution in a 15 mL centrifuge tube. The tube was mixed by inversion 3 times, and the microexplants were allowed to settle within the centrifuge tubes for at least 5 min. Due to a difference in size between the microtissues and the microgels, the microtissues settled more quickly than the isolated microgel particles. The settlement of microexplants could be visually observed, and once a pellet of microexplants had settled at the bottom of the tube, the supernatant was aspirated until ~1 mL of liquid was left above the settled samples. This step was repeated an additional 3 times to remove the vast majority of the microgel particles that made up the LLS. On the third wash, the supernatant was removed completely, and ice-bath cooled culture medium (2 mL) was added to proceed with the intended assays.

### 2.6. Immunofluorescence Assay

The immunofluorescence (IF) staining protocol was previously completed in [42]. In brief, microexplant samples were fixed in 4.0% formaldehyde in 1X PBS overnight at 4 °C, washed twice, and incubated in PBS for 1 h at room temperature. The samples were then permeabilized in 0.5% Triton X-100 (Sigma-Aldrich, St. Louis, MO, USA, X100-100ML) for 2 h, washed twice, and blocked with 3% bovine serum albumin in PBS for 3 h at room temperature. After blocking, samples were washed 3 times with PBS and then incubated overnight with conjugated antibodies at 4 °C. The antibodies used in this study included E-cadherin (BD Biosciences, San Jose, CA, USA, 560062) and Invitrogen™ Alexa Fluor™ 568 Phalloidin (Invitrogen, Waltham, MA, USA, A12380). After overnight incubation with the antibodies, the samples were washed 3 times with PBS and counterstained with Hoechst 33342 (Invitrogen, Waltham, MA, USA, H3570) for 15 min before imaging. For viability staining, a live/dead kit (Thermo Fisher, Waltham, MA, USA, R37601) consisting of Calcein AM and BOBO-3 iodide was used following the manufacturer’s protocol. All colorectal microtissues were then imaged using a Nikon A1R HD25 confocal microscope equipped with a high-definition Galvano scanner.

### 2.7. Semi-Quantification Analysis of Mouse Colon Microexplant Viability

Estimation of percent viability from IF images of microexplants (*n* = 3 for each culture condition) was manually performed using ImageJ. Particle count analysis from 2 channels, including TRITC (dead nuclei) and DAPI (all nuclei) was performed by thresholding. The ratio of dead nuclei to total cell nuclei was used to estimate the percent viability of all samples.

### 2.8. Flow Cytometry

Before perfusion culture, AC16 cells were labeled with CFSE to enable their separation from LLS during flow cytometry (CFSE Cell Division Tracker Kit, 423801; BioLegend, San Diego, CA, USA). The mixture of LLS and cells was suspended in PBS and then resuspended in flow cytometry buffer (15 mL PBS with 2% FBS and 1 mM EDTA). The suspension was filtered through a 100 μm Nitex nylon mesh (Genesee Scientific, San Diego, CA, USA, 57-103), washed in flow cytometry buffer, and then filtered again through a 40 μm cell strainer (Corning, Inc., Corning, NY, USA, 352340) into a 50 mL centrifuge tube to further remove LLS particles. The filtered LLS–cell suspension was centrifuged at 400× *g* for 5 min at room temperature, transferred to round-bottom 5 mL flow tubes, and then washed with DPBS for viability staining.

Samples were resuspended in 200 µL DPBS, stained with 1 µM of DAPI (BioLegend, San Diego, CA, USA, Cat. No. 422801) and incubated for 5 min at room temperature. Cells were analyzed by flow cytometry using a Cytoflex instrument (Beckman Coulter, Brea, CA, USA) equipped with the standard three-laser configuration (a 405 nm violet laser, a 488 nm blue laser, and a 638 nm red laser). To gate for viability, cells were heat-killed at 65 °C for 45 min, stained with DAPI, and recorded. Fluorescence-minus-one (FMO) controls were prepared for viability markers. Both unstained cells and LLS were also analyzed as a negative control.

Samples were gated to exclude doublets using forward scatter height (FSC-H) and width (FSC-W). The majority of the remaining LLS microgel particles were excluded from the analysis based on size and granularity by comparing forward scatter area (FSC-A) versus side scatter area (SSC-A) between the LLS-only control and samples. Viable single cells were then separated from residual LLS based on specific staining.

### 2.9. Statistical Analysis

Statistical analysis was performed using GraphPad Prism (version 9.3 Mac, GraphPad, San Diego, CA, USA). Comparisons of metabolic activity and viability for AC16 cells and analysis of contractility in explants were performed using a two-way ANOVA with Dunnett’s post-test to determine significance. Comparisons of colon microexplant viability were performed using Brown–Forsythe ANOVA with Dunnett’s post-test to determine significant differences. A probability value of *p* < 0.05 was considered statistically significant.

## 3. Results

### 3.1. Three-Dimensional Cell Culture Platform

**Figure 1:**
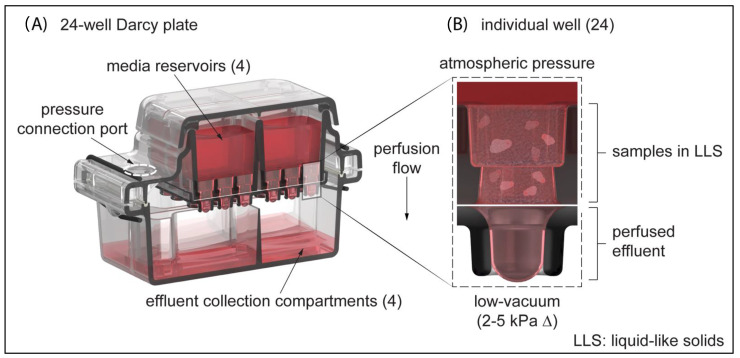
24-Well Darcy Plate (**A**) A 24-well plate consists of 4 injection-molded polystyrene components: a lid, media reservoir, droplet-guiding cover, and effluent collection chambers. The lid protects the media reservoirs from sources of contamination and allows the exchange of gases. The media reservoir is divided into quadrants that each can contain up to 6 mL of culture media. (**B**) Detailed view of an individual well where samples can be suspended in LLS. A PCTE membrane is heat-sealed against the bottom surface during the plate assembly process, securing the suspension in place. A pressure device is used to create a light-vacuum pressure within the effluent collection compartments that drives the perfusion of media through the LLS and PCTE membrane.

We developed an injection-molded polystyrene perfusion plate assembly that provides an accessible platform for long-term ex vivo culture of cells and tissue microexplants in 3D. As shown in Figure 1, these perfusion plates have four media reservoirs, each of which supplies six wells (24 wells per plate). Each media reservoir accommodates up to 6 mL of media, allowing for up to four different experimental conditions to be conducted in parallel. The suspension of biological samples can potentially be facilitated by a variety of permeable extracellular matrix scaffolds. In this study, we used a soft granular bed of aqueous microgels known as liquid-like solids (LLS) [40]. We employed a closed-suction medical device (e.g., a Jackson-Pratt wound drainage bulb) as a “negative pressure” source to actively perfuse liquid media through the interstitial spaces in the LLS and collect effluent into a sealed chamber with isolated collection wells. Darcy’s law, which characterizes fluid flow through a porous medium, was used to determine appropriate flow-channel geometries and media volumes:

(1) $$Q=\frac{\left(k\cdot A\cdot \Delta P\right)}{µ\cdot L}$$

Flow rate Q is a function of the cross-sectional area A of the well, the permeability constant k, the pressure differential ΔP, the dynamic viscosity µ, and the height of the channel L. The permeability constant k was found to be a function of the granular particle size as well as the modulus of the microgel particles, which is controlled by the polymer content in the microgels. The dynamic viscosity μ of cell culture media was approximately that of water and in the range of 0.8–1.0 mPa-s [43]. The individual well width was based on a standard single-well diameter from a 96-well plate (dimensioned as 6 mm), and the channel height was 7 mm. This channel geometry accommodates up to 200 µL of sample volume per well and allows modulation of the perfusion flow rate by varying the height of the LLS column. We named these perfusion plates Darcy plates, in honor of Henry Darcy and Darcy’s Law.

### 3.2. Rapid Generation of Cellular Spheroids in Perfusion Culture

**Figure 2:**
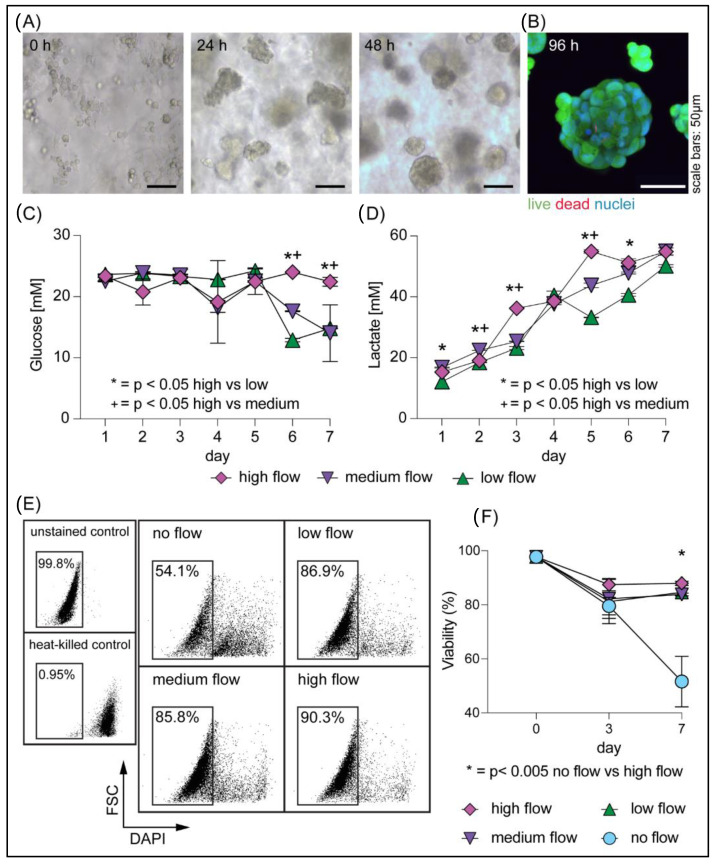
Single-cell human cardiomyocytes cultured in 3D in LLS: (**A**) Phase contrast microscopy images of AC16 cells cultured in a 24-well Darcy plate at 0, 24, and 48 h showing cellular aggregation and spheroid formation. (**B**) Viability of AC16 spheroids on day 4 was assessed by fluorescent microscopy using Calcein AM (live) and BOBO-3 Iodide (dead). (**C**) Glucose consumption and (**D**) lactate secretion were measured from two technical replicates of daily effluent media collection, revealing metabolic activities of the AC16 cells. Statistical analysis was performed using two-way ANOVA with comparisons to the high flow condition to assess significance at each time point. (**E**,**F**) The percentage of cell viability was assessed by flow cytometry. (**E**) Characteristic flow profiles for all four conditions for one representative replicate on day 7 (typical for all samples, *n* = 3 replicates for each condition). The insets on the left show an example of a gating strategy used for assessing viability including the use of a heat-killed control. (**F**) Proportion of viable cells at day 0, day 3, and day 7 from different flow rates, 25.4 ± 4.1 (low), 35.3 ± 4.1 (medium), and 43.1 ± 4.8 µL/hr/well (high) for all samples, confirms significant viability of the cell populations in the presence of perfusion flow. Triplicate wells were used to measure viability in each condition, and two-way ANOVA with Dunnett’s post-test was used for statistical analysis.

We first cultured the rapidly proliferating and metabolically active AC16 human cardiomyocyte cell line to determine the optimal cell culture conditions for the 24-well Darcy plate. A suspension of AC16 cells (10^6^ cells/mL) in LLS was cultured for 1 week; at this cell-seeding density, we observed the presence of cellular aggregates after 24 h and dense spheroid formations at 48 h (Figure 2A). After 2 days of culture, we were able to observe a reduction in effluent medium pH, as reflected by the change in its color. This potentially served as an indication of metabolic activity. We next sought to determine the optimal ranges of flow rates and determine whether there were any detrimental mechanical stresses to the cells as a result of increased flow rate (e.g., flow-induced mechanical shear stress) [44]. We applied various flow rates of growth media (25.4 ± 4.1, 35.3 ± 4.1, and 43.1 ± 4.8 µL/hr/well) to the culture for 7 days. We assessed cell viability via Calcein AM, a membrane-permeable viability dye that only fluoresces in metabolically active cells, and BOBO-3^TM^ Iodide, a cell-impermeant dye that specifically labels the nuclei of dead cells. The micrograph in Figure 2B reveals remarkable viability for all flow conditions, confirming that the perfusion flow rates selected for our platform did not impart harmful stresses on AC16 cells. Furthermore, measurements of glucose and lactate from effluent media were used to determine the daily metabolic activities of the samples under different flow conditions. As shown in Figure 2C,D, both daily stable fluctuations in glucose consumption and an increasing trend in lactate secretions were observed, which indicated healthy metabolic activity by AC16 cells in perfusion culture. We also observed a reduction in glucose levels on days 6 and 7 of culture in the slower flow rate (25.4 ± 4.1 and 35.3 ± 4.1 µL/hr/well) conditions, suggesting that the reduced nutrient flux in these wells was nearing the limit of the metabolic needs of the cells. We further confirmed the viability of the culture by flow cytometry (Figure 2E) and revealed that all cells that were cultured under perfusion were >80% viable on day 7, as compared to cells that were cultured in LLS without perfusion, which were 50% viable at the same time point (Figure 2F). These results emphasize the importance of media exchange through perfusion for the long-term culture of cells in 3D. Given the highly proliferative and metabolically active nature of the AC16 cell line, we determined that the initial cell seeding density and the presented flow rates may be broadly applicable to the culture of other cell types.

### 3.3. Long-Term Ex Vivo Culture of Microexplants in 3D

**Figure 3:**
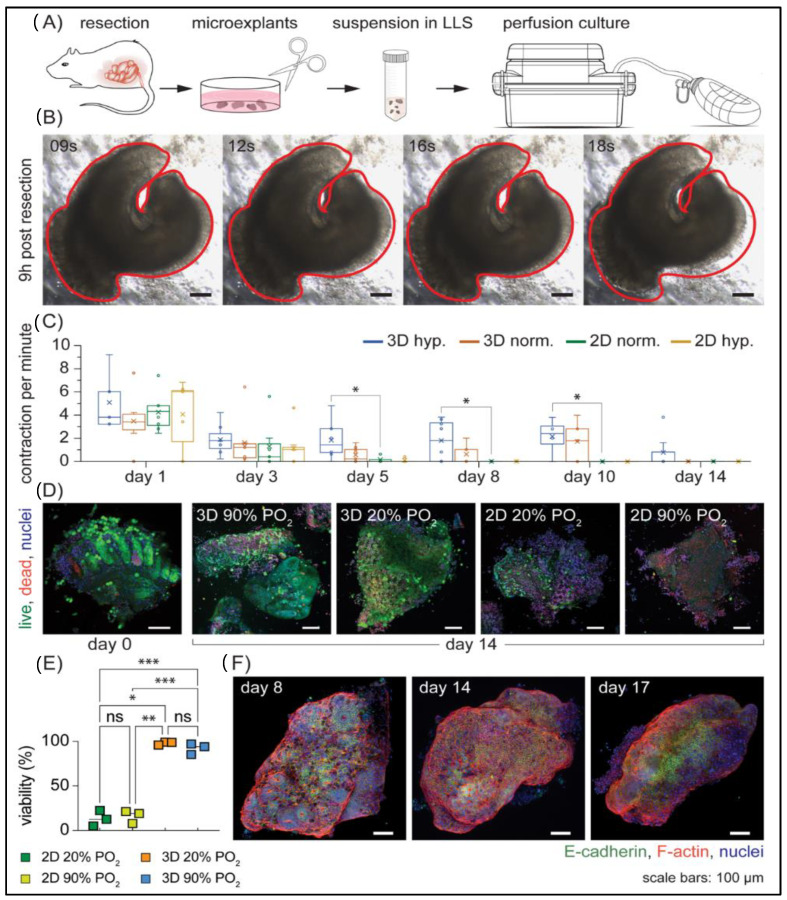
Ex vivo culture of microexplants: (**A**) Diagram outlining major steps of mouse colorectal microtissue fabrication and culture in perfusion platform. (**B**) A series of time-lapse microscopy images showing the periodic contraction of a representative mouse colorectal microexplant from collected samples (*n* = 20) that had been cultured under perfusion for 9 h. Contraction frequency was estimated to be 3 contractions per minute (cpm) and was recorded at 1 fps for 10 min. (**C**) Assessment of microexplant contractility (4 ≤ *n* ≤ 9 per condition, * = *p* < 0.05) under 4 culture conditions: 3D hyperoxic (90% PO_2_) and normoxic (20% PO_2_), and 2D hyperoxic (90% PO_2_) and normoxic (20% PO_2_). Measurements were compared to the 2D 20% PO_2_ to determine significance. (**D**) Micrographs of the viability of microexplants by fluorescent microscopy using Calcein AM (live) and BOBO-3 Iodide (dead). (**E**) Quantification of D (*n* = 3 per condition, * = *p* < 0.05, ** = *p* < 0.01, and *** = *p* < 0.001). (**F**) Representative microexplants from the 3D hyperoxic condition at days 8, 14, and 17, stained with Hoechst 33342 (nuclei), Alexa Fluor™ 568 Phalloidin (F-actin), and E-cadherin (epithelial cells), demonstrate the well-preserved heterogenous microexplant structure. Data in Figure 3 were collected from three separate mice and are representative of 5 experiments using 7 mice in total.

Encouraged by the successful culture of AC16 cells, we next tested the feasibility of extending the viability of mouse colorectal microexplants under perfusion. We reasoned that both oxygen and nutrient availability would be important for tissue viability. This idea of increasing oxygen partial pressure to increase oxygen availability is not new; various groups have reported varying oxygen partial pressures for their culture platforms [12,13,16,17,45,46,47], which further emphasizes the importance of this parameter (Table 1). Understanding that the final transport process in solid tissues is diffusion, we limited the largest dimension of our explants to 1 mm. Gut explants have previously been cultured by simply submerging them in liquid media with some success [16,17]; therefore, we used this approach as a control to compare the viability of explants in both liquid-cover culture (which we term 2D) in liquid media and under perfusion in LLS (which we term 3D) (Figure 3A). In total, we selected four conditions: perfusion culture under hyperoxic (90% PO_2_) and normoxic conditions (20% PO_2_), and liquid-cover culture under hyperoxic (90% PO_2_) and normoxic conditions (20% PO_2_). All of our experiments reproduced the tissue contractions (Figure 3B, Video S1, and Figure S1) as reported in the literature [17,45,46]. Overall, the presence and frequency of contractions appeared random between microtissue samples, but these observations of persistent contractions coupled with immunofluorescent data (Figure 3C–E) confirmed that the explants were both viable and functional. Microtissues cultured in 2D under both normoxic and hyperoxic conditions exhibited no contractility by day 5, while samples cultured under perfusion consistently demonstrated contractility after 10 days of culture, averaging 1.7 and 2.1 contractions per minute (cpm) respectively (Figure 3C). As shown in Table 2, the average observed contraction rates for randomly selected samples from 3D hyperoxic conditions were similar for days 3, 5, and 8 (at 1.88, 1.83, and 1.78 cpm respectively) and were higher than those cultured in all other conditions. Although the observed differences in contraction between the two 3D conditions were minimal, the presence of tissue contraction was detected on day 14 only for hyperoxic samples (Table 2), suggesting higher PO_2_ might be beneficial for mouse colon microtissue explants in 3D culture under perfusion. These results correlated with immunofluorescent viability assay data and were consistent with well-preserved viability in all 3D conditions (Figure 3D,E). Furthermore, immunostaining for the epithelial cell marker E-cadherin on day 17 (Figure 3F) confirmed that this system of Darcy plates, LLS, and perfusion supported the survival of epithelial cells. Taken together, these data suggest that perfusion culture can sustain a microtissue explant for an extended period.

## 4. Discussion

In this study, we introduced an in vitro platform in which cell lines and microtissue explants can be cultured in 3D by means of perfusion. The culture of the human cardiomyocyte AC16 cell resulted in significant viability (~90%) and rapid spheroid formation due to cellular aggregation (Figure 2), suggesting that this platform could also serve as a novel tool for cell culture and spheroid generation. Additionally, perfusion culture enabled the maintenance of resected mouse colorectal tissue ex vivo for an extended period. Notably, tissue heterogeneity, an important criterion for tissue explant models, was well maintained, as revealed by functional smooth muscle tissue contraction and the presence of epithelial cells within the colorectal microexplants at days 14 and 17 of culture (Figure 3). The ability to culture gut microexplants was demonstrated by the sustained viability and prolonged contractility for more than 2 weeks (Figure 3 and Figure S1, Table 2), comparable to previous attempts reported in the literature, such as catenary cultures [17], membrane inserts [46,47], and air–liquid interface intermittent exposure [12], as summarized in Table 1. However, the human or rodent intestinal tissues cultured in those platforms were restricted to larger sizes (>1 mm). Moreover, a common culture mechanism from these existing platforms relies on the interchange of macromolecules (e.g., growth factors and waste metabolites) within an abundant liquid medium. In contrast, the culture mechanism reported here provides a constant supply of fresh media and the removal of metabolic waste by means of continuous directional perfusion. Our injection-molded 24-well Darcy plate facilitates the culture of multiple microexplants (less than 1 mm in the longest dimension) in a single well. This potentially offers a wider range of experimental conditions especially when the collected sample is limited in size. We envision using this platform for the culture and expansion of stem cells and other tissue microexplants including those derived from patient samples.

The engineering principles behind the 24-well Darcy plate were guided by the need to be easily integrated within existing cell culture infrastructure and disposable at the end of the experiment. The Darcy plate presented here is an assembly of disposable injection-molded polystyrene parts (Figure 1) that incorporates 24 vertical wells equally distributed amongst four separate media reservoirs. This design allows for one environmental condition shared by all wells, four liquid media formulations shared by six wells each, and unique samples and microenvironments in each of the six wells. Uniform perfusion of media across all wells can be maintained by a passive pressure device (e.g., wound drainage bulb) which facilitates straightforward operation and avoidance of potential electronic malfunctions. Unlike existing platforms (e.g., microfluidics), each well of the perfusion plate can accommodate a broad range of cell densities and microtissue sizes, enabling greater freedom in experimental design. This is made possible due to the controllable flow rates of media through the system based on modulation of the LLS volume, its particle size distribution, or adjustment of the applied pressures. We developed this platform by applying biofabrication technology developed to support the precise printing of delicate structures in a completely self-healing 3D medium using liquid-like solids (LLS) [40]. In contrast to the rapid convective mixing that occurs in 2D culture with liquid media, convection is suppressed and Fickian diffusion is slower in the LLS. Therefore, perfusion is needed to ensure the transport of growth factors, cytokines, and metabolic waste. The interstitial spaces between the LLS particles recapitulate a capillary bed and enable the transport of small molecules and proteins facilitated by a controlled pressure gradient-driven perfusion process. While a steady flow of liquid media permeates the interstitial spaces between microgel particles, the low yield-stress property of the LLS acts as a support medium and allows unimpeded growth of multicellular structures, cell–cell interactions, and the production of ECM.

## 5. Conclusions

We developed a 24-well injection-molded polystyrene plate assembly, namely the Darcy plate, to provide an accessible platform for long-term ex vivo culture of cells and tissue microexplants in 3D. The working mechanism of the plate is enabled by means of continuous directional perfusion through a 3D support medium of granular microgels, allowing the constant supply of culture media and removal of cell-secreted metabolic waste. The uniform perfusion of media is facilitated by a pump-free suction device to avoid complex electronic accessories or burdensome tubing networks. We demonstrated the application of the Darcy plate by culturing the AC16 human cardiomyocyte cell line for 7 days and mouse gut microtissues for more than 14 days. The culture of highly proliferative AC16 cells revealed rapid spheroid formation after 48 h and ~90% viability by day 7 (Figure 2). We further tested the versatile application of the Darcy platform by maintaining the mouse gut microexplants in ex vivo culture for more than 2 weeks (Figure 3). The cultured gut microexplants were highly viable after 14 days as shown by the immunofluorescent viability assay. Notably, tissue heterogeneity was maintained as demonstrated by the presence of epithelial cells and prolonged smooth muscle contractility that was reported in the literature [17,45,46]. The Darcy plate platform may potentiate 3D cell culture as a simpler and more accessible approach.

## Figures and Tables

**Table 1:** Summary of existing gut explant culture platforms and culture conditions.

Platform	Culture Condition	Size	Duration	Note
Air–liquid interface	37 °C, 5%CO_2_, 95% O_2_	n/a	24 h	The interchange of macromolecules in the culture medium was defined as an important factor [12].
Rocker system	37 °C, 5%CO_2_, 95% O_2_	5 × 5 mm	3 months	The rocker system allowed the samples to be intermittently exposed to the air and prevented the accumulation of mucus, digestive enzyme, hormones, and growth factor by agitation. The technique allowed the culture of rat colon explants for up to 3 months [14], and human colon explants for up to 20 days [13].
Rocker system	37 °C, 5%CO_2_, 95% O_2_	5 × 5 mm	20 days	
Catenary culture	37 °C, 5%CO_2_, 95% Air	Whole section	10 days	Embryonic gut segments were cultured in suspension while immersed in media. The cultured segments showed random contraction after day 6 [17].
Free-floating culture	37 °C, 5%CO_2_, 95% Air	Whole gut	14 days	The whole embryonic mouse gut was cultured as a “free-floating organ”. Peristalsis was observed after 48 h [45].
Air–liquid interface	37 °C, 5%CO_2_, 95% Air	2–3 mm^3^	12 days	The samples were cultured on a submerged gel foam raft. Human colon explants with epithelial and muscular mucosae were cultured for HIV-1 infection study [48].
Immersion culture	37 °C, 5%CO_2_, 95% Air	Whole section	11 days	Mouse embryo intestinal explants were plated on fibronectin-coated coverslips and immersed in liquid media [16].
Membrane insert	37 °C, 5%CO_2_, 95% Air	200 µm slices	14 days	The contraction was maintained up to day 14. After 2 weeks of culture, treatment with 100 μM of serotonin and 10 μM of calcium channel blocker tetrodotoxin increased and reduced contraction, respectively [46].
Membrane insert	37 °C, 5%CO_2_, 95% Air	2 mm^2^	14 days	Murine colon segments were cultured in liquid-cover media for up to 2 weeks [49].
Membrane insert	37 °C, 5%CO_2_, 95% Air	12 mm^2^	35 days	Culture of colon segments from SCID mice by membrane insert technique [47].

**Table 2:** Contraction of mouse colorectal explants during long-term culture. (a) Average observable contraction per minute. (b) The maximum number of contractions per minute observable through brightfield microscopy. The data show a comparison between 3D vs. 2D culture at two oxygen partial pressures (20% and 90%). The explants cultured in 3D were able to maintain the contraction for 14 days, while those in 2D culture stopped the motion after 5 days of culture.

**(a)**				
**Duration of Culture (Days)**	**3-D**		**2-D**	
	**PO_2_ (%)**		**PO_2_ (%)**	
	**90%**	**20%**	**90%**	**20%**
3	1.88	1.60	1.14	1.29
5	1.83	0.57	0.07	0.17
8	1.78	0.60	-	-
14	0.77	-	-	-
**(b)**				
**Duration of Culture (Days)**	**3-D Perfusion**		**2-D**	
	**PO_2_ (%)**		**PO_2_ (%)**	
	**90%**	**20%**	**90%**	**20%**
3	4.2	6.4	4.6	5.6
5	4.8	1.6	0.4	0.6
8	3.8	2	-	-
14	3.8	-	-	-

## Data Availability

Not applicable.

## Supplementary Materials

The following supporting information can be downloaded at: https://www.mdpi.com/article/10.3390/cells11060967/s1, Figure S1: A panel showing gut explant morphology cultured in 2D vs 3D at different timepoints. Supplemental Video S1: A representative mouse gut microexplant cultured in perfusion platform for 3 days. Supplemental Video for Figure 3B: time-lapse video of gut contraction.
