# Supplementary figures and images for: 3D In Vitro Platform for Cell and Explant Culture in Liquid-like Solids (part 2 of 2)
Source: Cells. 2022 Mar 11;11(6):967. doi: 10.3390/cells11060967 (PMC8946834; doi:10.3390/cells11060967)

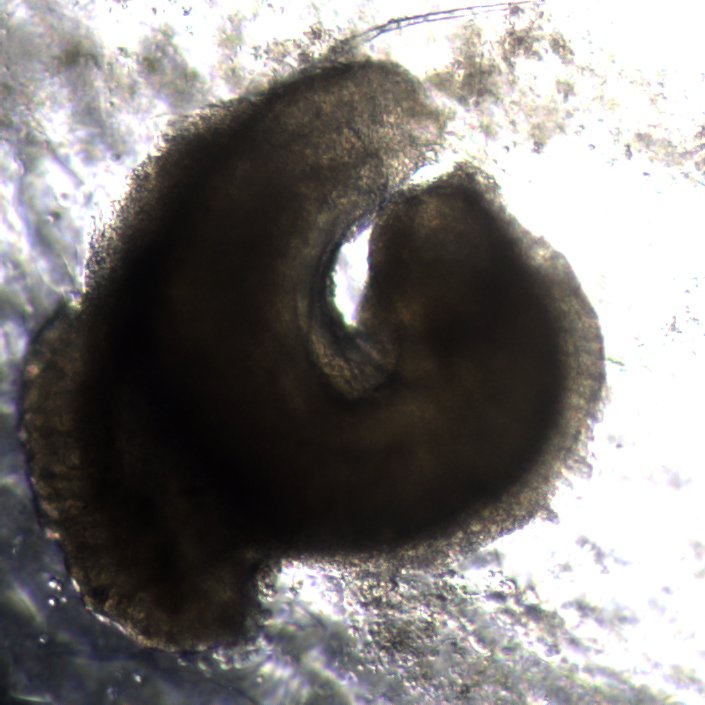

Supplement: Supplementary file 1 [file cells-11-00967-s001.zip › supplemetal videos/figure 3B mouse gut explant contraction JPEG time-lapse images/lobsterClaw533.jpg]

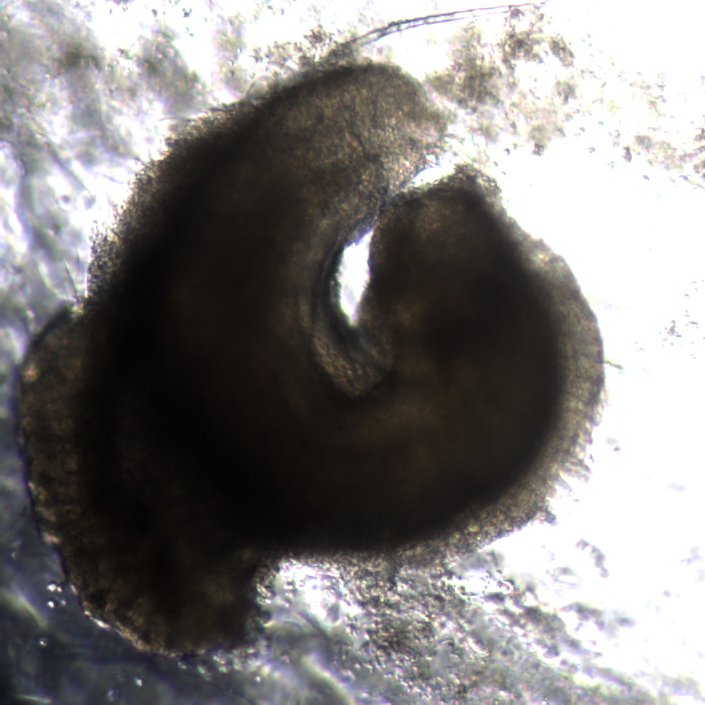

Supplement: Supplementary file 1 [file cells-11-00967-s001.zip › supplemetal videos/figure 3B mouse gut explant contraction JPEG time-lapse images/lobsterClaw255.jpg]

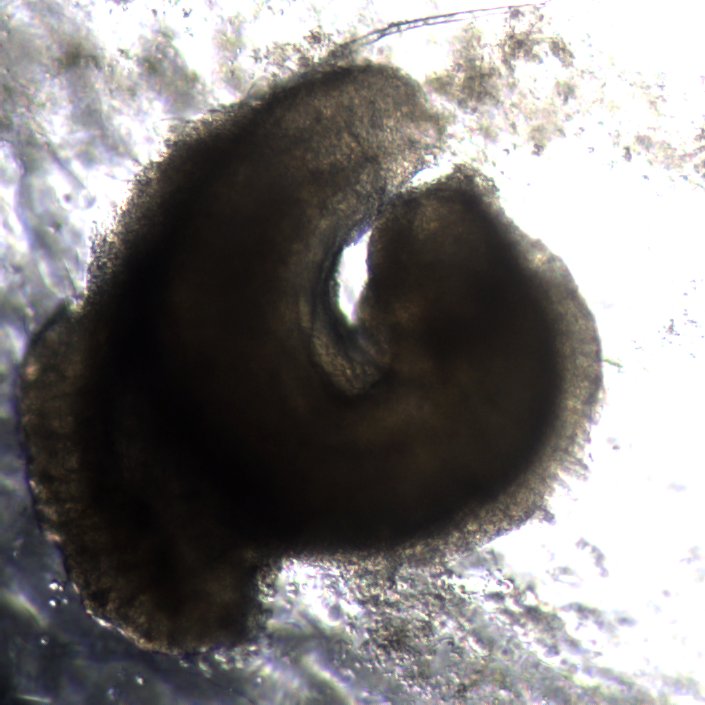

Supplement: Supplementary file 1 [file cells-11-00967-s001.zip › supplemetal videos/figure 3B mouse gut explant contraction JPEG time-lapse images/lobsterClaw282.jpg]

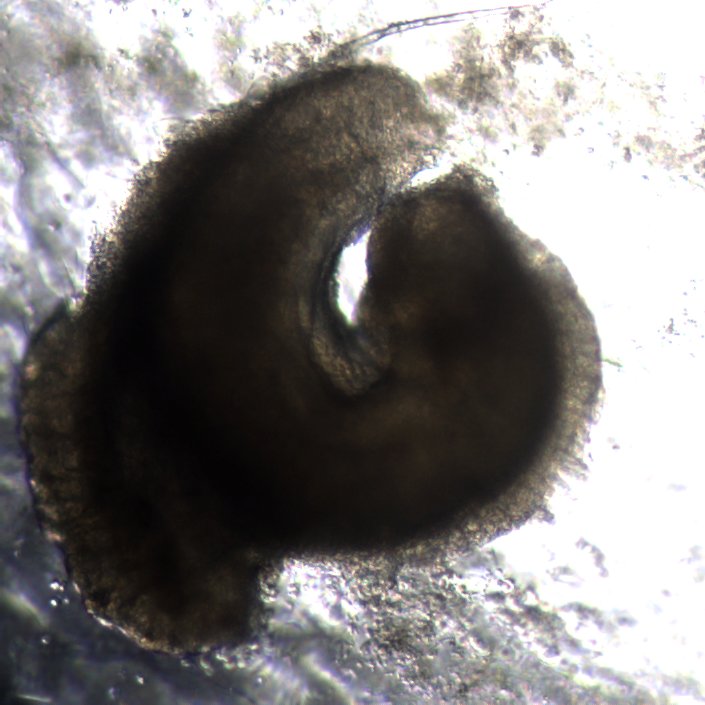

Supplement: Supplementary file 1 [file cells-11-00967-s001.zip › supplemetal videos/figure 3B mouse gut explant contraction JPEG time-lapse images/lobsterClaw296.jpg]

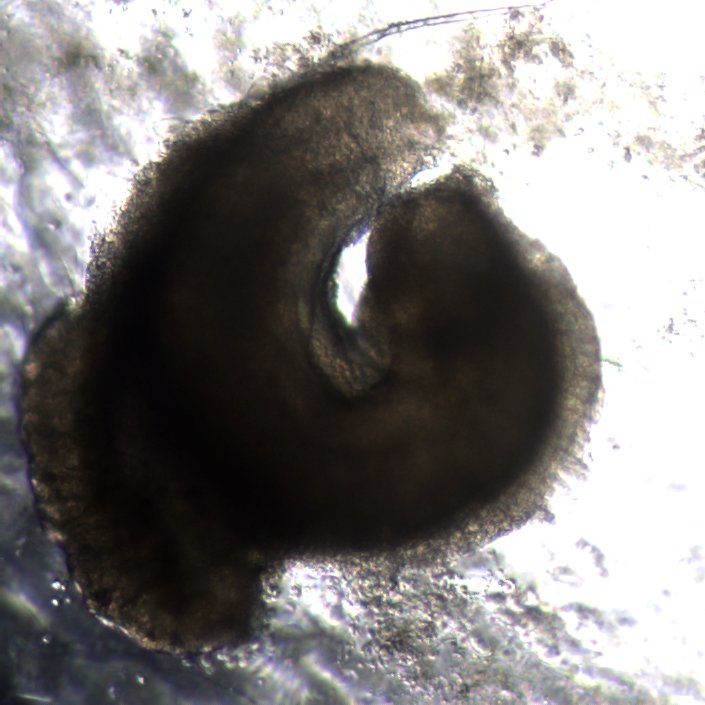

Supplement: Supplementary file 1 [file cells-11-00967-s001.zip › supplemetal videos/figure 3B mouse gut explant contraction JPEG time-lapse images/lobsterClaw297.jpg]

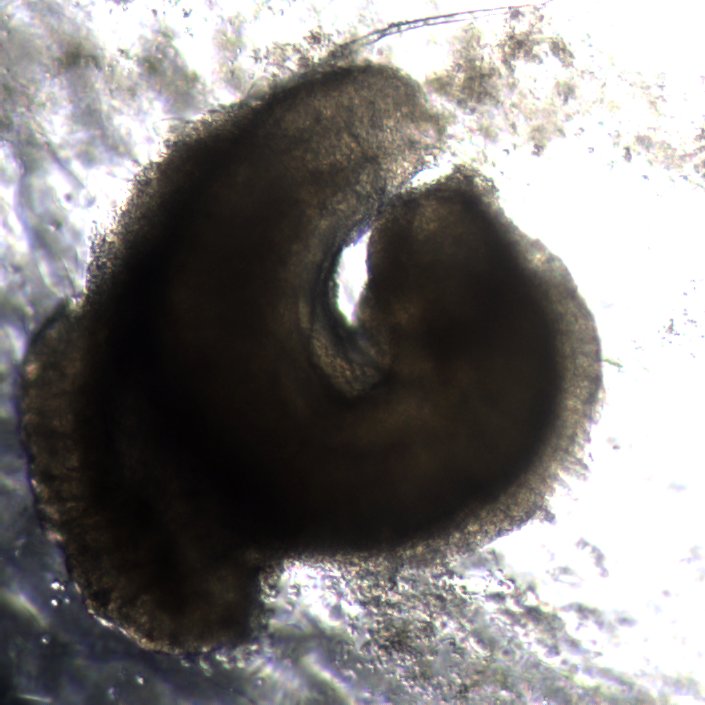

Supplement: Supplementary file 1 [file cells-11-00967-s001.zip › supplemetal videos/figure 3B mouse gut explant contraction JPEG time-lapse images/lobsterClaw283.jpg]

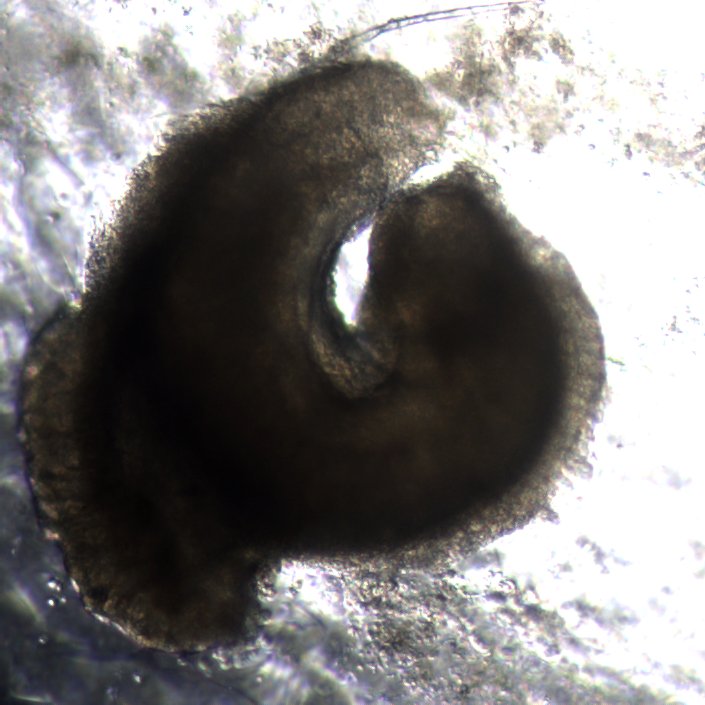

Supplement: Supplementary file 1 [file cells-11-00967-s001.zip › supplemetal videos/figure 3B mouse gut explant contraction JPEG time-lapse images/lobsterClaw532.jpg]

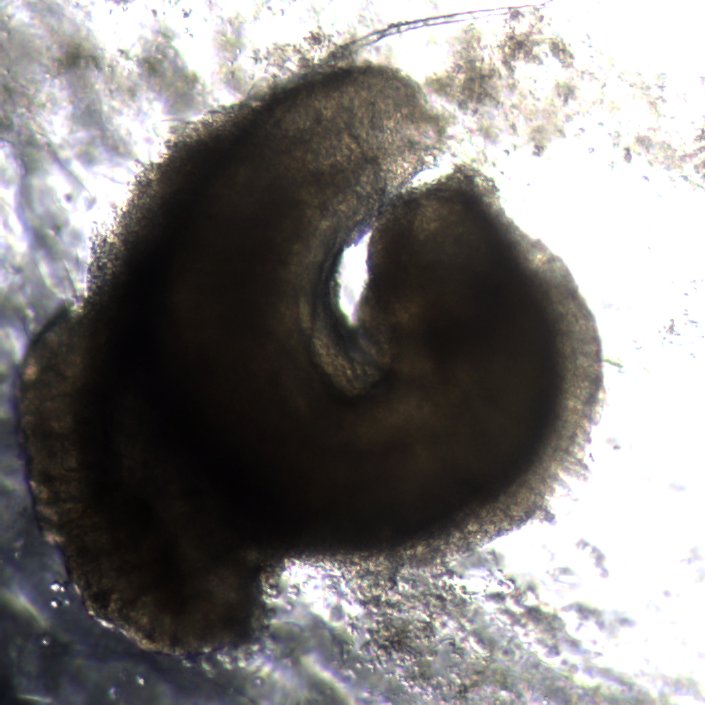

Supplement: Supplementary file 1 [file cells-11-00967-s001.zip › supplemetal videos/figure 3B mouse gut explant contraction JPEG time-lapse images/lobsterClaw254.jpg]

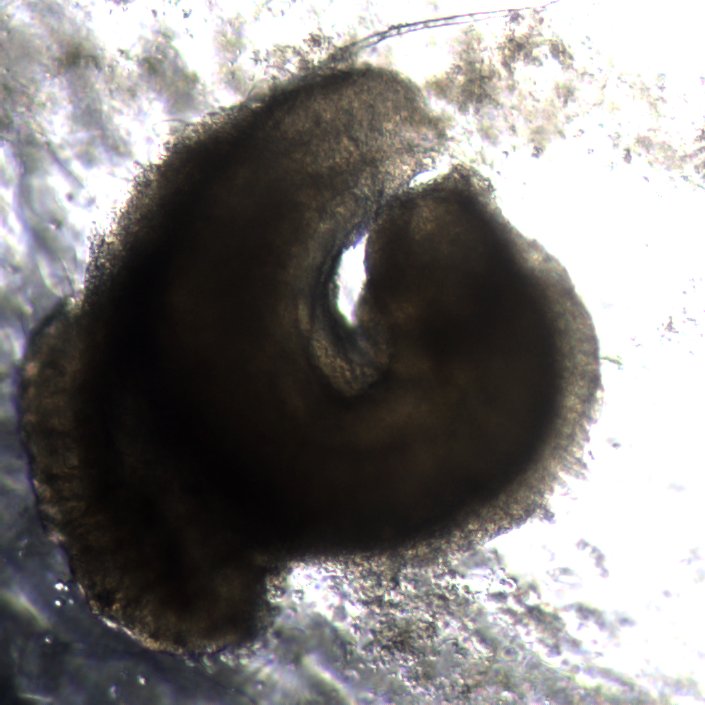

Supplement: Supplementary file 1 [file cells-11-00967-s001.zip › supplemetal videos/figure 3B mouse gut explant contraction JPEG time-lapse images/lobsterClaw240.jpg]

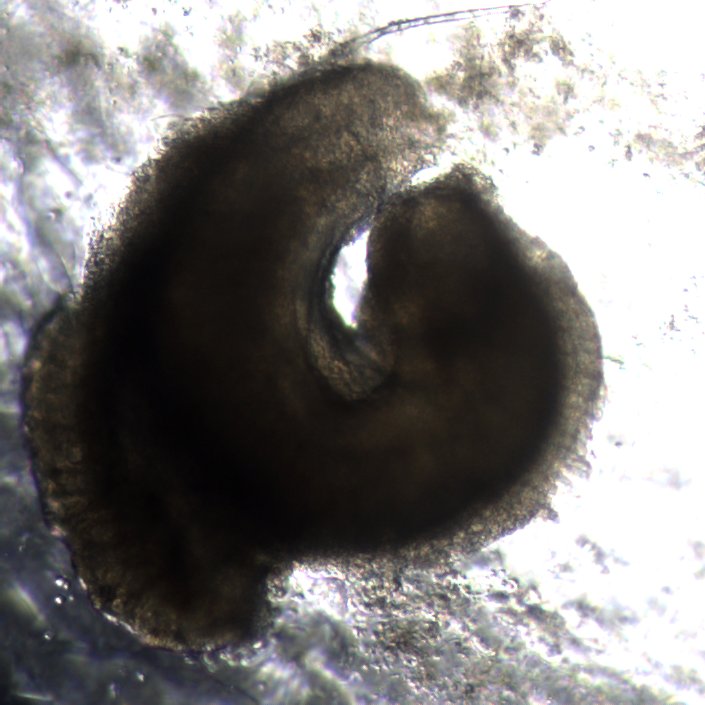

Supplement: Supplementary file 1 [file cells-11-00967-s001.zip › supplemetal videos/figure 3B mouse gut explant contraction JPEG time-lapse images/lobsterClaw526.jpg]

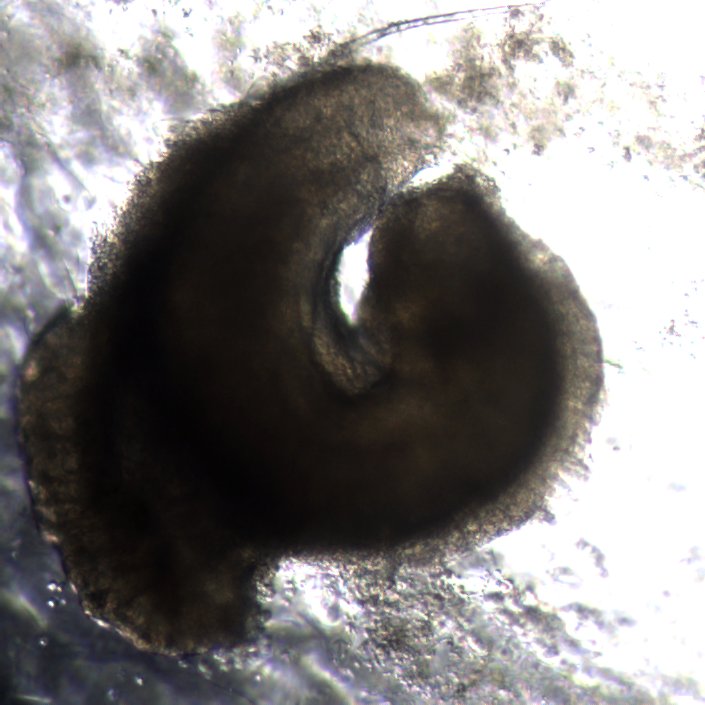

Supplement: Supplementary file 1 [file cells-11-00967-s001.zip › supplemetal videos/figure 3B mouse gut explant contraction JPEG time-lapse images/lobsterClaw268.jpg]

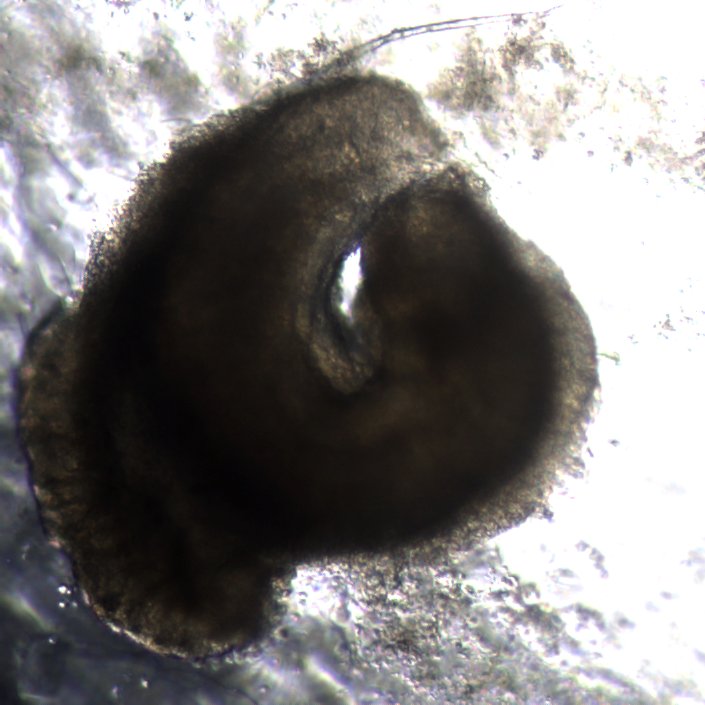

Supplement: Supplementary file 1 [file cells-11-00967-s001.zip › supplemetal videos/figure 3B mouse gut explant contraction JPEG time-lapse images/lobsterClaw095.jpg]

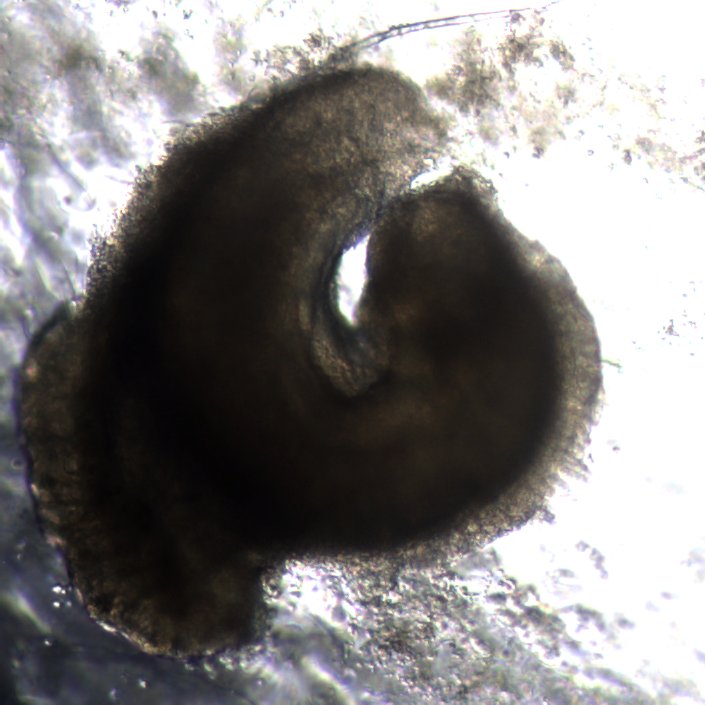

Supplement: Supplementary file 1 [file cells-11-00967-s001.zip › supplemetal videos/figure 3B mouse gut explant contraction JPEG time-lapse images/lobsterClaw081.jpg]

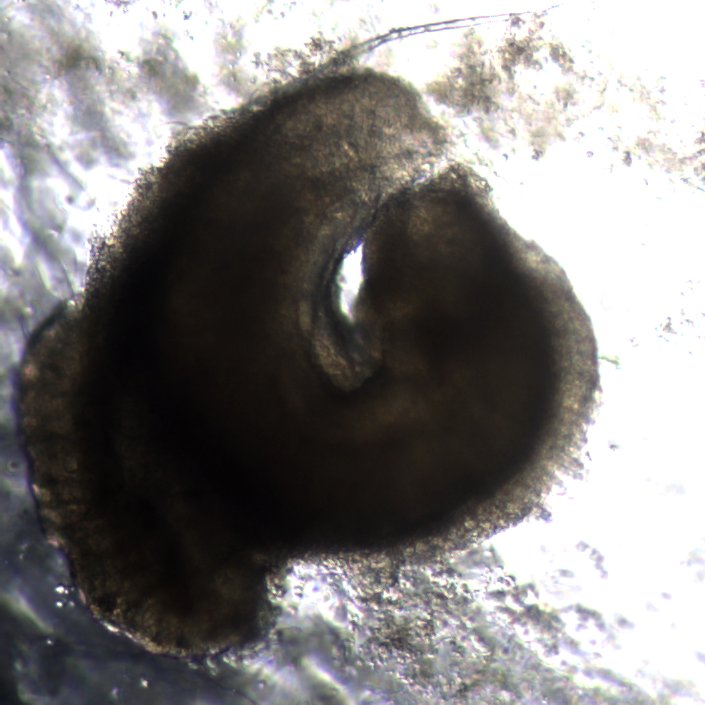

Supplement: Supplementary file 1 [file cells-11-00967-s001.zip › supplemetal videos/figure 3B mouse gut explant contraction JPEG time-lapse images/lobsterClaw056.jpg]

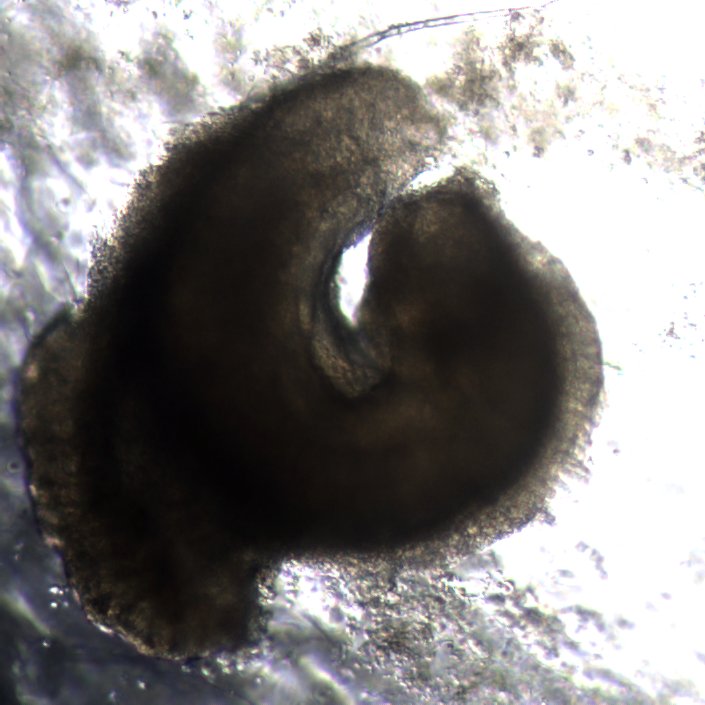

Supplement: Supplementary file 1 [file cells-11-00967-s001.zip › supplemetal videos/figure 3B mouse gut explant contraction JPEG time-lapse images/lobsterClaw042.jpg]

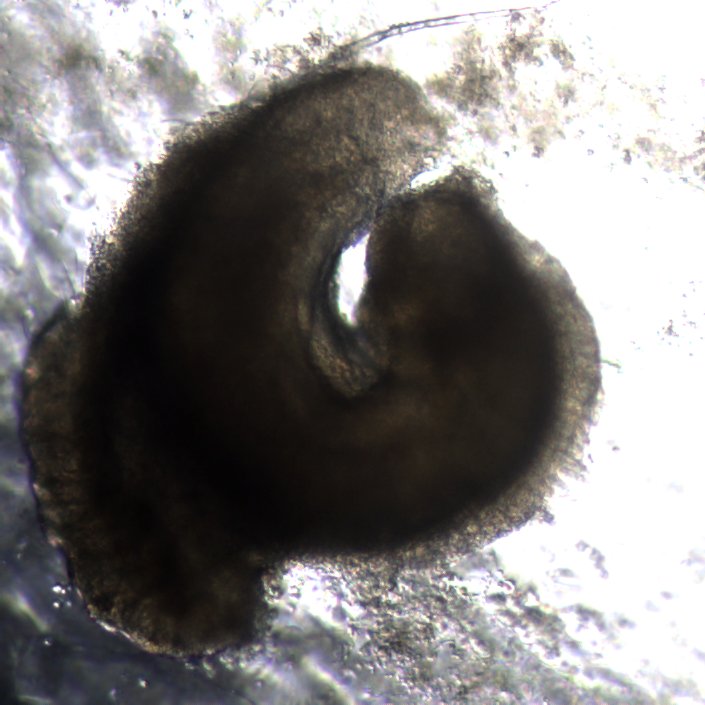

Supplement: Supplementary file 1 [file cells-11-00967-s001.zip › supplemetal videos/figure 3B mouse gut explant contraction JPEG time-lapse images/lobsterClaw122.jpg]

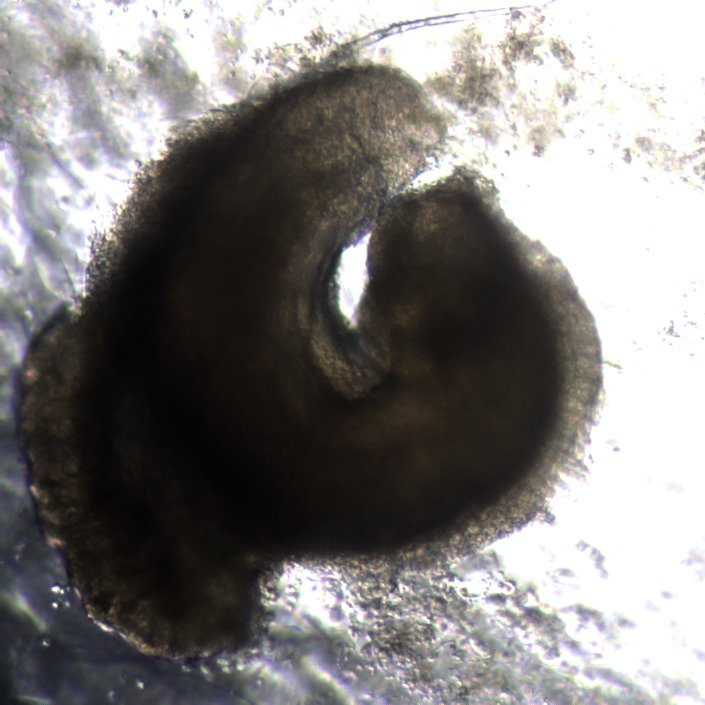

Supplement: Supplementary file 1 [file cells-11-00967-s001.zip › supplemetal videos/figure 3B mouse gut explant contraction JPEG time-lapse images/lobsterClaw136.jpg]

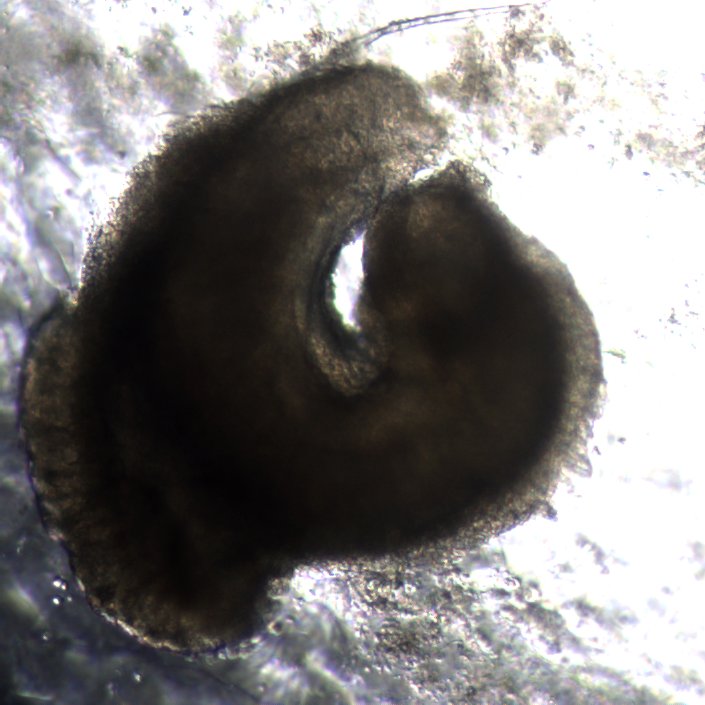

Supplement: Supplementary file 1 [file cells-11-00967-s001.zip › supplemetal videos/figure 3B mouse gut explant contraction JPEG time-lapse images/lobsterClaw485.jpg]

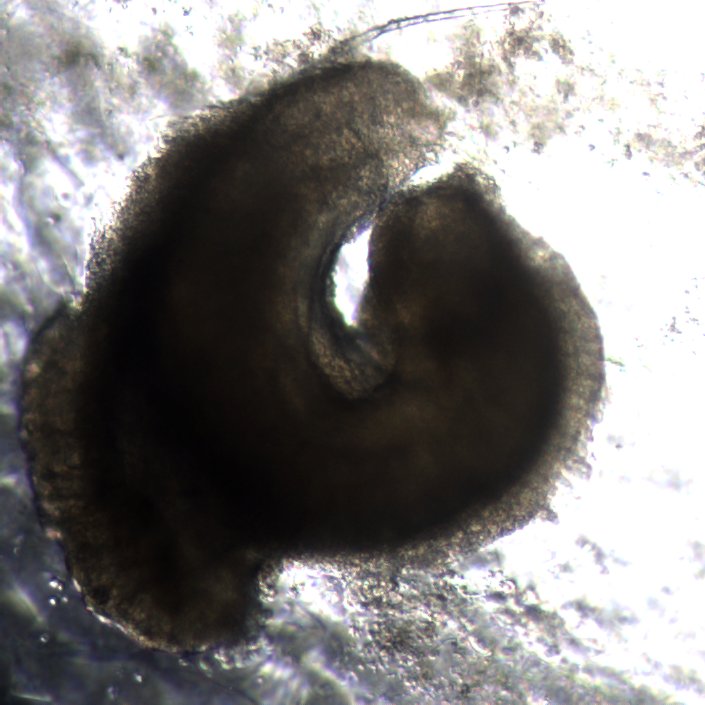

Supplement: Supplementary file 1 [file cells-11-00967-s001.zip › supplemetal videos/figure 3B mouse gut explant contraction JPEG time-lapse images/lobsterClaw491.jpg]

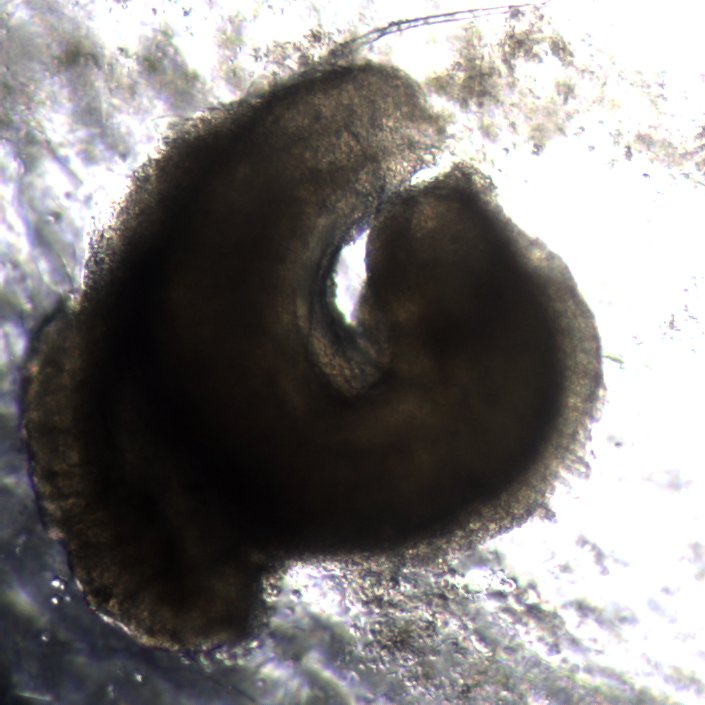

Supplement: Supplementary file 1 [file cells-11-00967-s001.zip › supplemetal videos/figure 3B mouse gut explant contraction JPEG time-lapse images/lobsterClaw446.jpg]

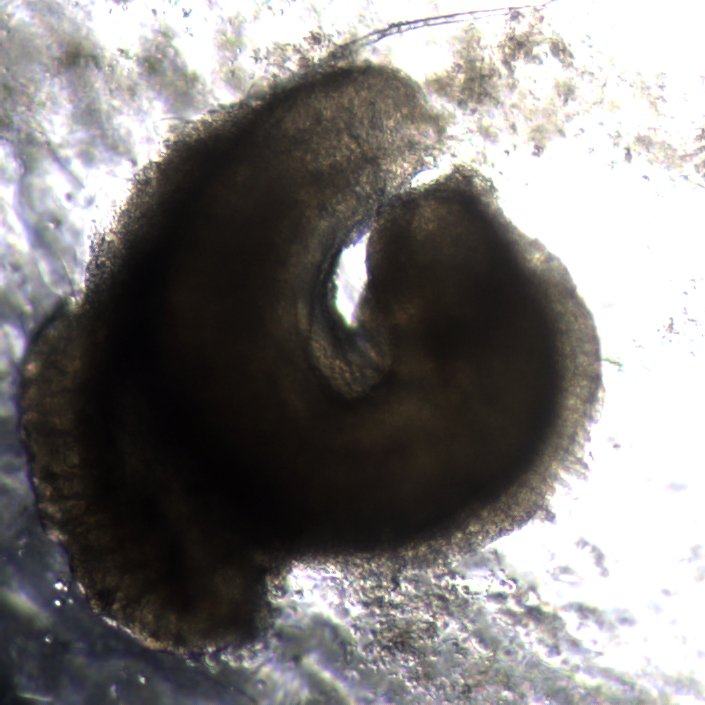

Supplement: Supplementary file 1 [file cells-11-00967-s001.zip › supplemetal videos/figure 3B mouse gut explant contraction JPEG time-lapse images/lobsterClaw320.jpg]

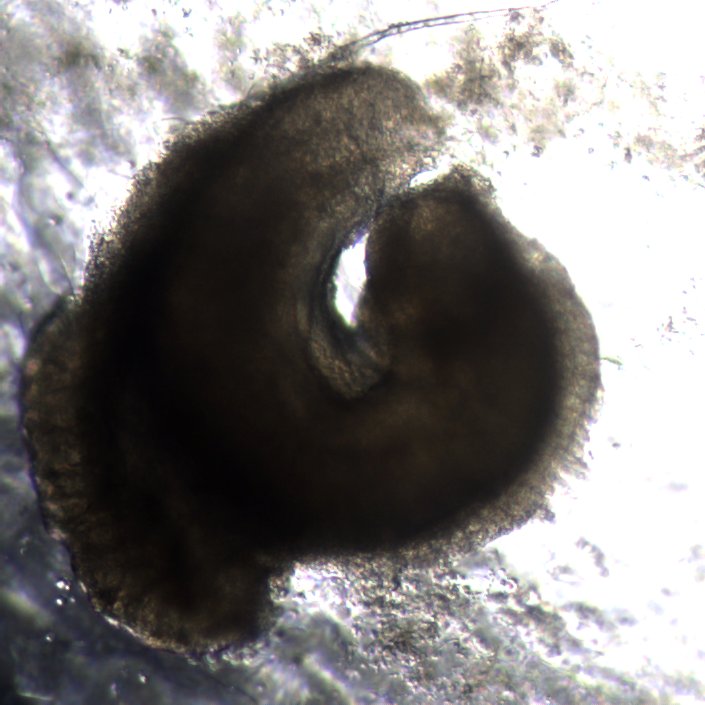

Supplement: Supplementary file 1 [file cells-11-00967-s001.zip › supplemetal videos/figure 3B mouse gut explant contraction JPEG time-lapse images/lobsterClaw334.jpg]

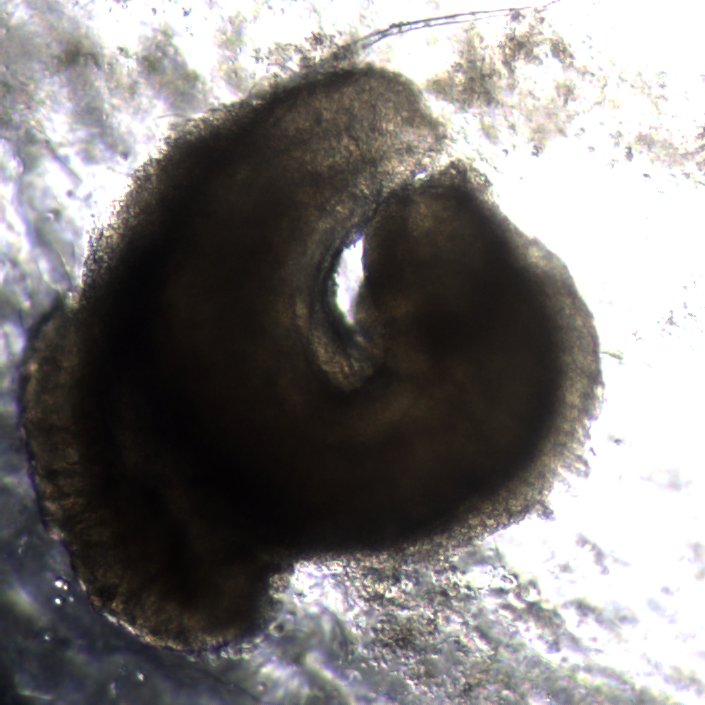

Supplement: Supplementary file 1 [file cells-11-00967-s001.zip › supplemetal videos/figure 3B mouse gut explant contraction JPEG time-lapse images/lobsterClaw452.jpg]

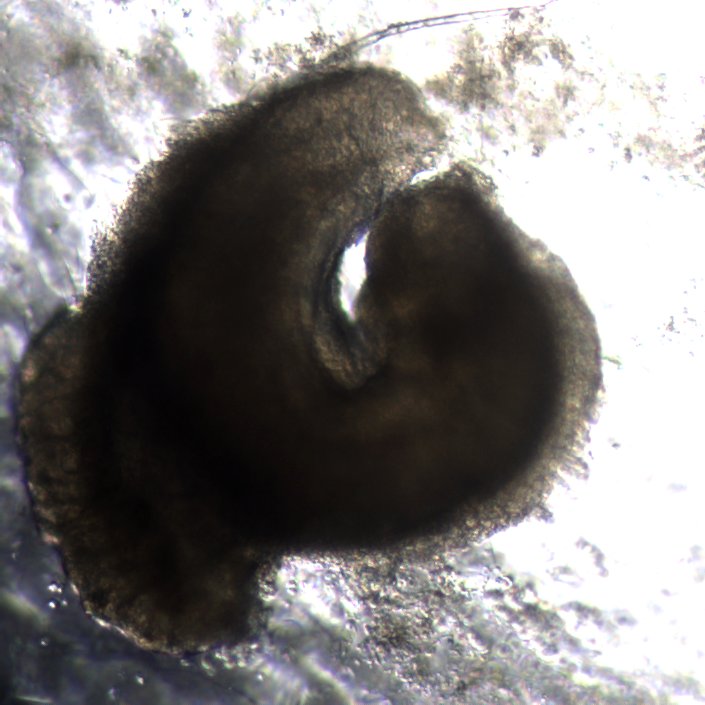

Supplement: Supplementary file 1 [file cells-11-00967-s001.zip › supplemetal videos/figure 3B mouse gut explant contraction JPEG time-lapse images/lobsterClaw308.jpg]

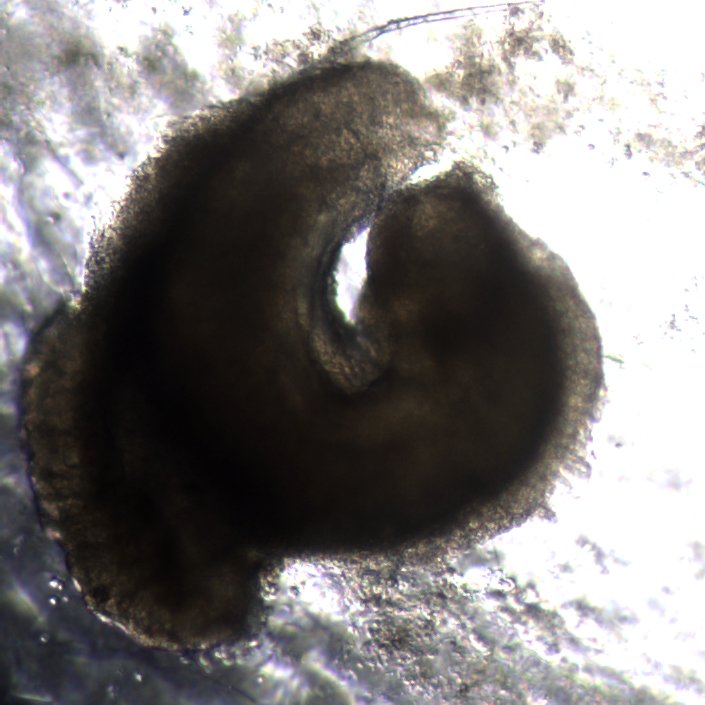

Supplement: Supplementary file 1 [file cells-11-00967-s001.zip › supplemetal videos/figure 3B mouse gut explant contraction JPEG time-lapse images/lobsterClaw478.jpg]

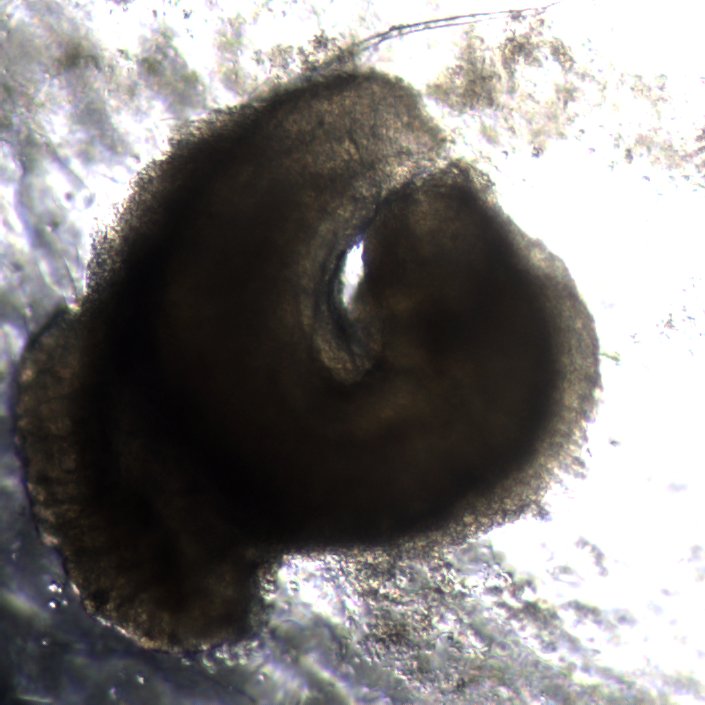

Supplement: Supplementary file 1 [file cells-11-00967-s001.zip › supplemetal videos/figure 3B mouse gut explant contraction JPEG time-lapse images/lobsterClaw322.jpg]

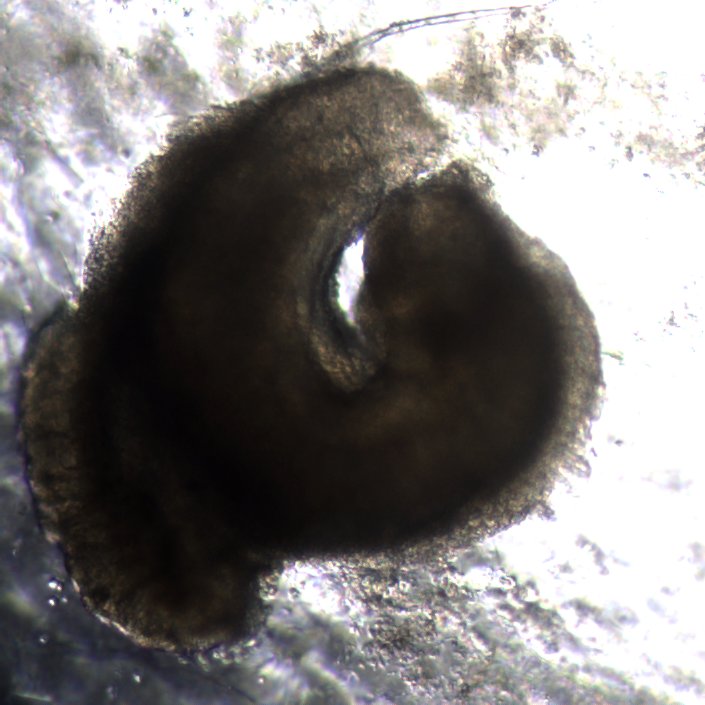

Supplement: Supplementary file 1 [file cells-11-00967-s001.zip › supplemetal videos/figure 3B mouse gut explant contraction JPEG time-lapse images/lobsterClaw444.jpg]

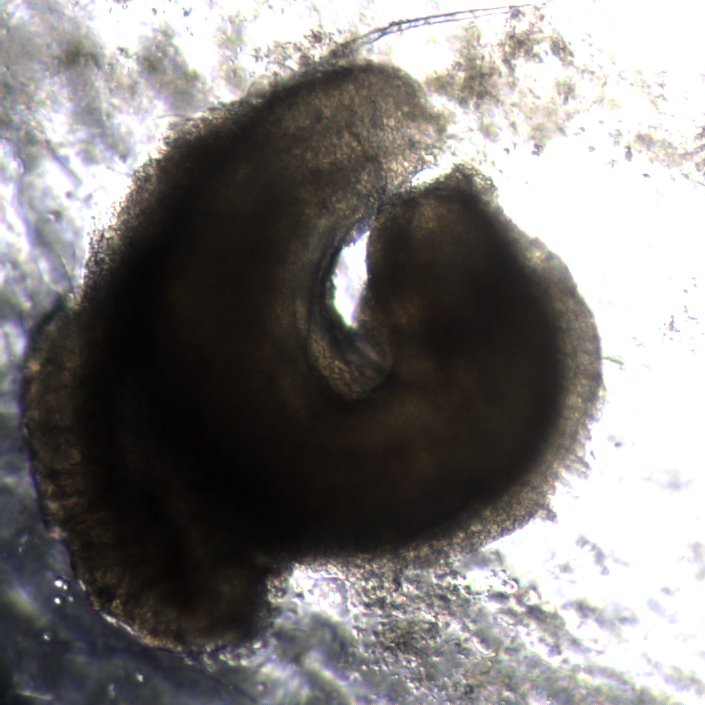

Supplement: Supplementary file 1 [file cells-11-00967-s001.zip › supplemetal videos/figure 3B mouse gut explant contraction JPEG time-lapse images/lobsterClaw450.jpg]

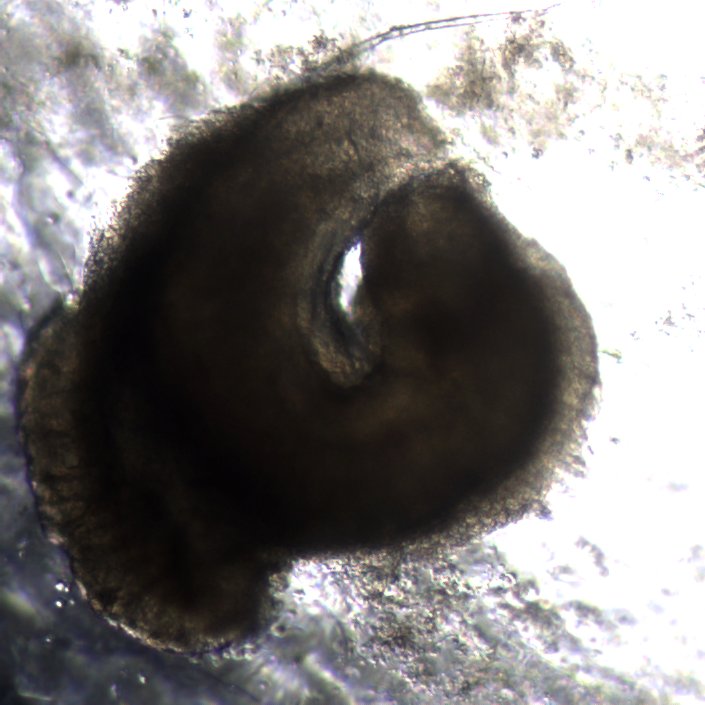

Supplement: Supplementary file 1 [file cells-11-00967-s001.zip › supplemetal videos/figure 3B mouse gut explant contraction JPEG time-lapse images/lobsterClaw336.jpg]

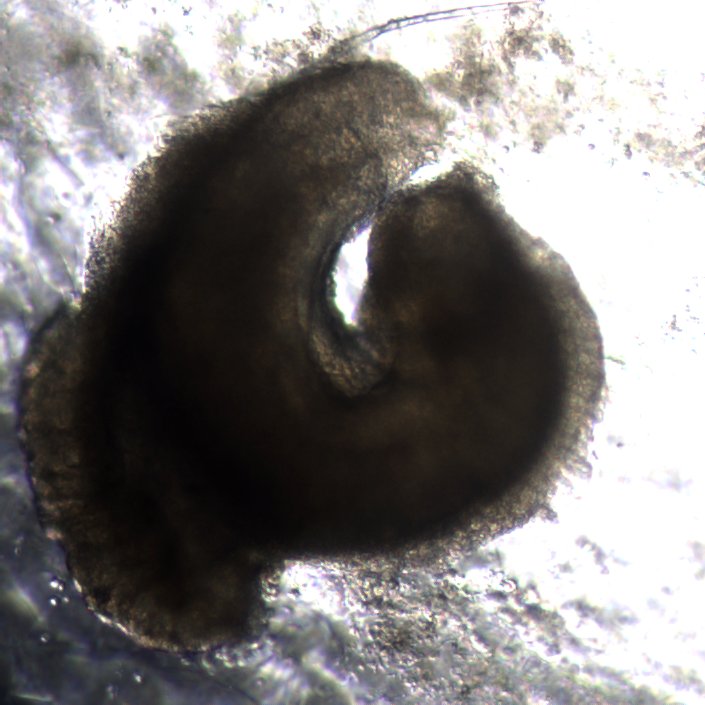

Supplement: Supplementary file 1 [file cells-11-00967-s001.zip › supplemetal videos/figure 3B mouse gut explant contraction JPEG time-lapse images/lobsterClaw487.jpg]

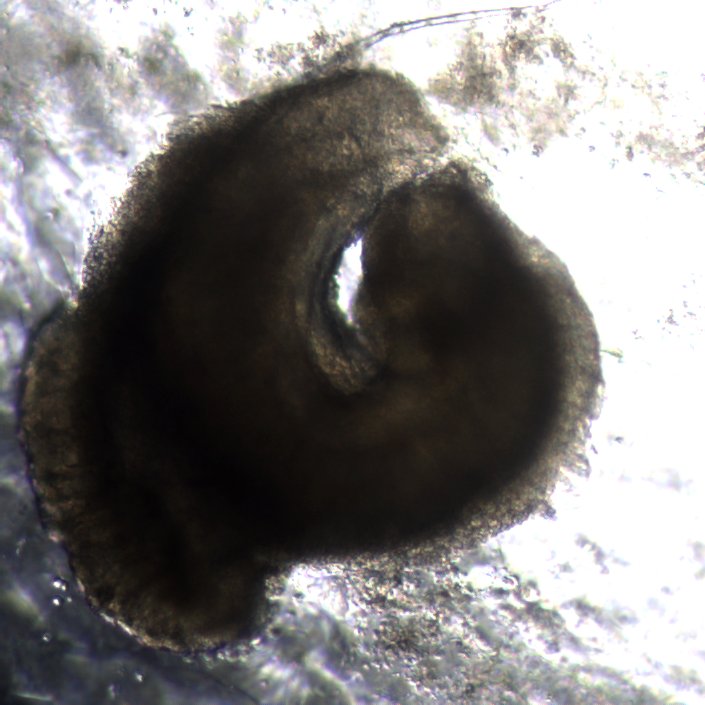

Supplement: Supplementary file 1 [file cells-11-00967-s001.zip › supplemetal videos/figure 3B mouse gut explant contraction JPEG time-lapse images/lobsterClaw493.jpg]

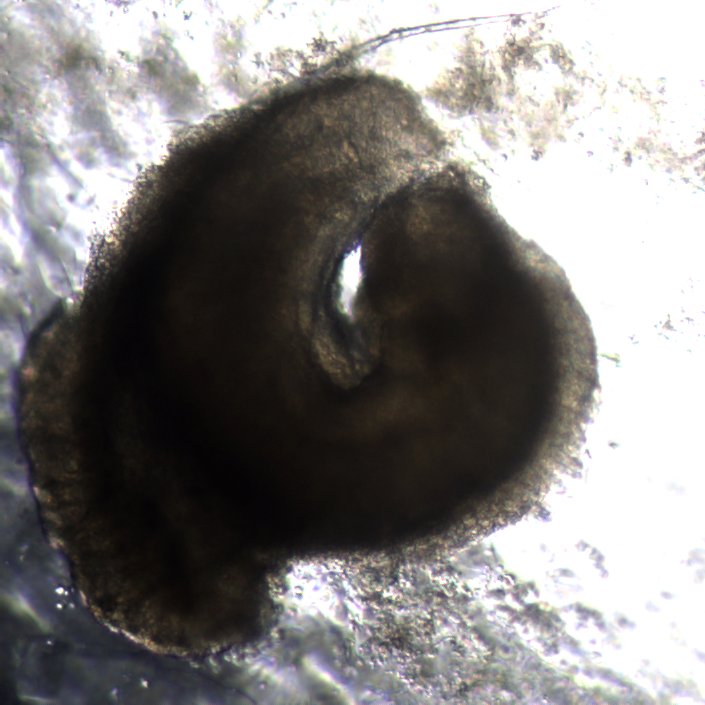

Supplement: Supplementary file 1 [file cells-11-00967-s001.zip › supplemetal videos/figure 3B mouse gut explant contraction JPEG time-lapse images/lobsterClaw108.jpg]

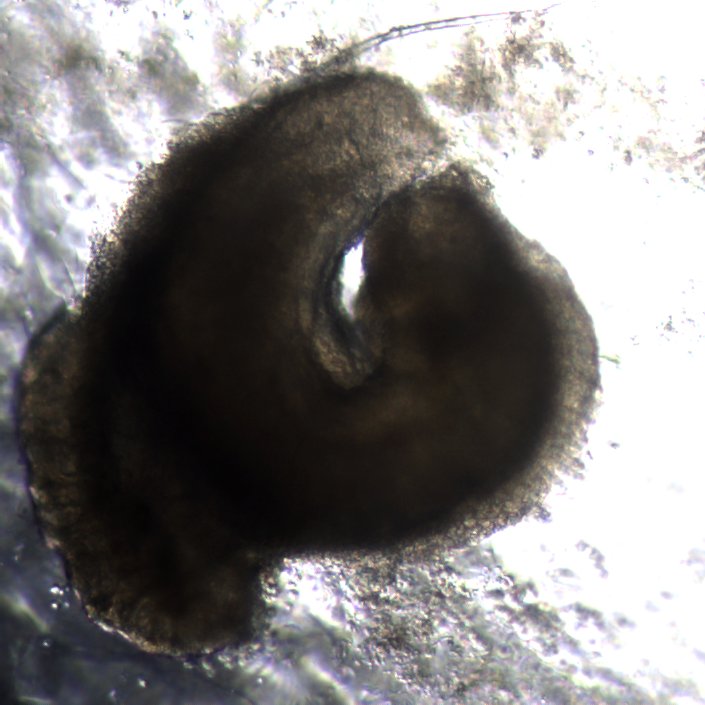

Supplement: Supplementary file 1 [file cells-11-00967-s001.zip › supplemetal videos/figure 3B mouse gut explant contraction JPEG time-lapse images/lobsterClaw120.jpg]

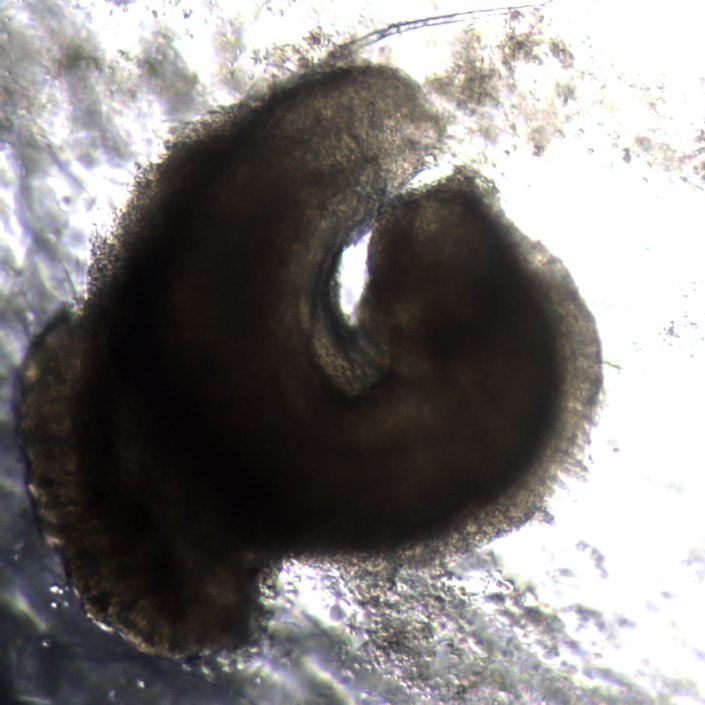

Supplement: Supplementary file 1 [file cells-11-00967-s001.zip › supplemetal videos/figure 3B mouse gut explant contraction JPEG time-lapse images/lobsterClaw134.jpg]

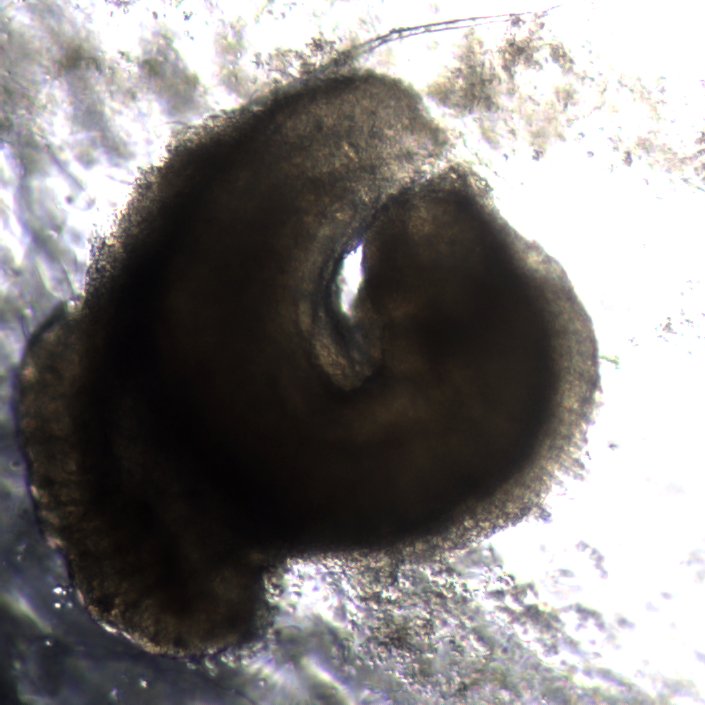

Supplement: Supplementary file 1 [file cells-11-00967-s001.zip › supplemetal videos/figure 3B mouse gut explant contraction JPEG time-lapse images/lobsterClaw068.jpg]

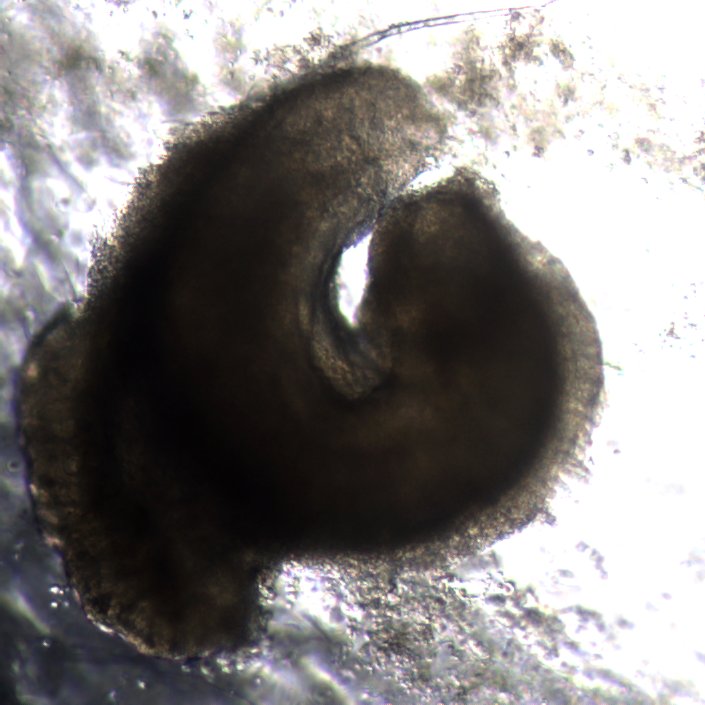

Supplement: Supplementary file 1 [file cells-11-00967-s001.zip › supplemetal videos/figure 3B mouse gut explant contraction JPEG time-lapse images/lobsterClaw054.jpg]

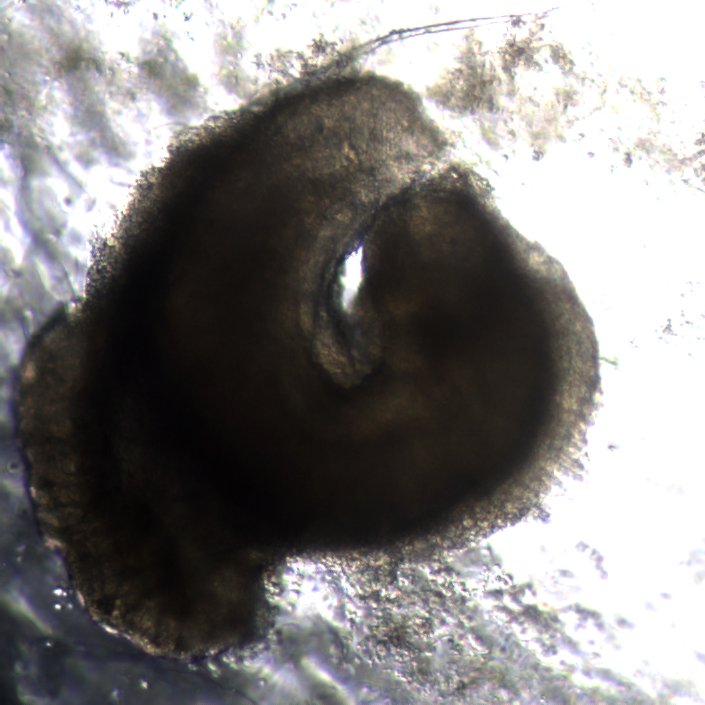

Supplement: Supplementary file 1 [file cells-11-00967-s001.zip › supplemetal videos/figure 3B mouse gut explant contraction JPEG time-lapse images/lobsterClaw040.jpg]

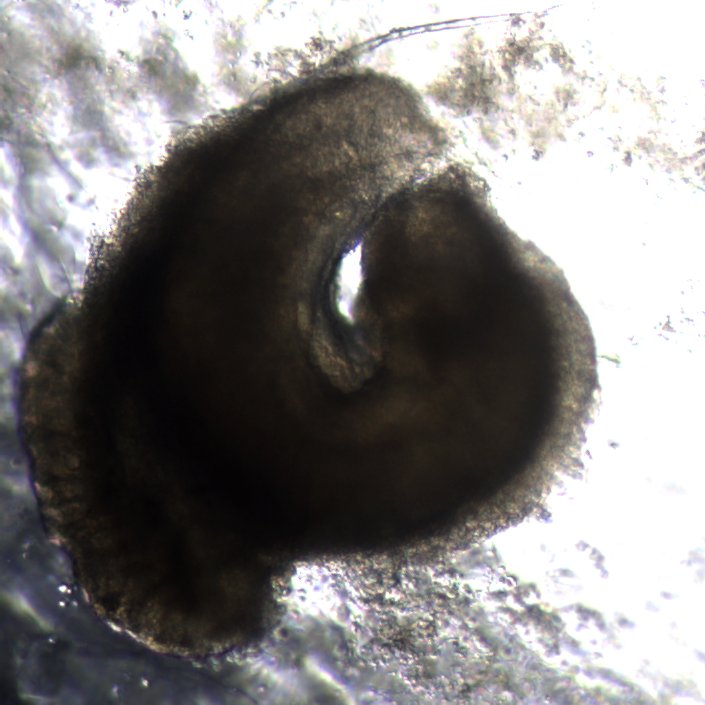

Supplement: Supplementary file 1 [file cells-11-00967-s001.zip › supplemetal videos/figure 3B mouse gut explant contraction JPEG time-lapse images/lobsterClaw097.jpg]

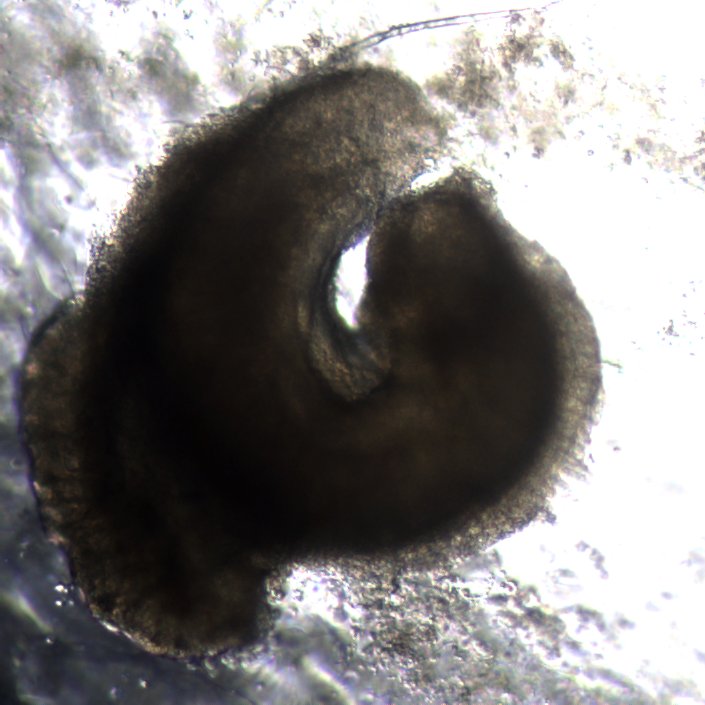

Supplement: Supplementary file 1 [file cells-11-00967-s001.zip › supplemetal videos/figure 3B mouse gut explant contraction JPEG time-lapse images/lobsterClaw083.jpg]

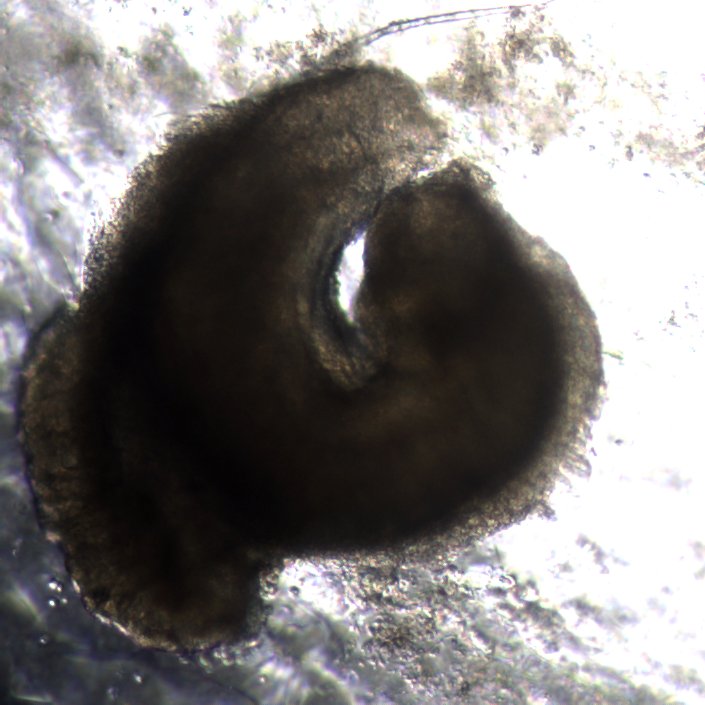

Supplement: Supplementary file 1 [file cells-11-00967-s001.zip › supplemetal videos/figure 3B mouse gut explant contraction JPEG time-lapse images/lobsterClaw518.jpg]

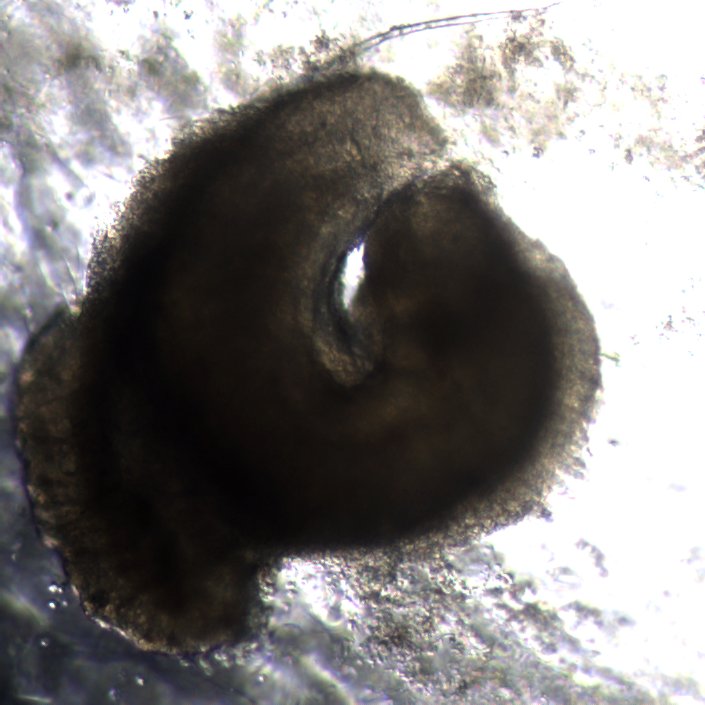

Supplement: Supplementary file 1 [file cells-11-00967-s001.zip › supplemetal videos/figure 3B mouse gut explant contraction JPEG time-lapse images/lobsterClaw256.jpg]

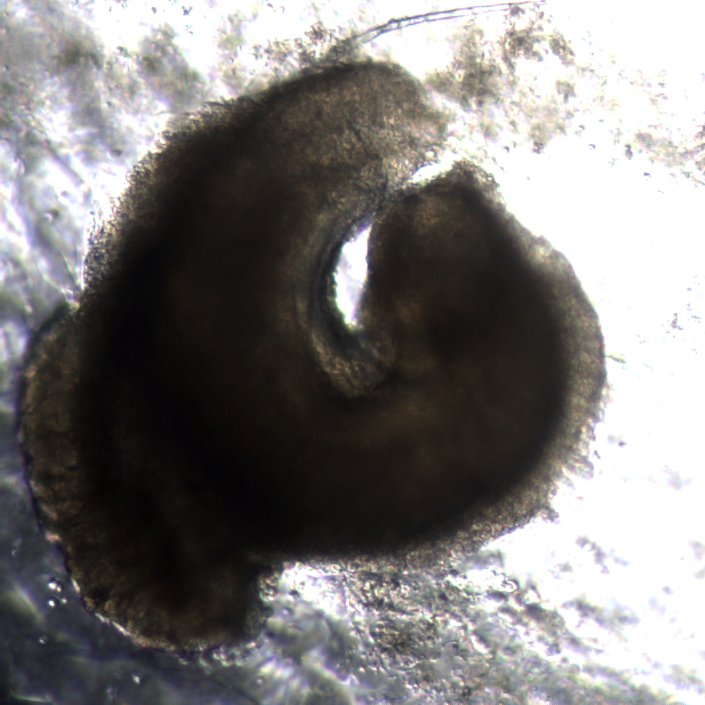

Supplement: Supplementary file 1 [file cells-11-00967-s001.zip › supplemetal videos/figure 3B mouse gut explant contraction JPEG time-lapse images/lobsterClaw530.jpg]

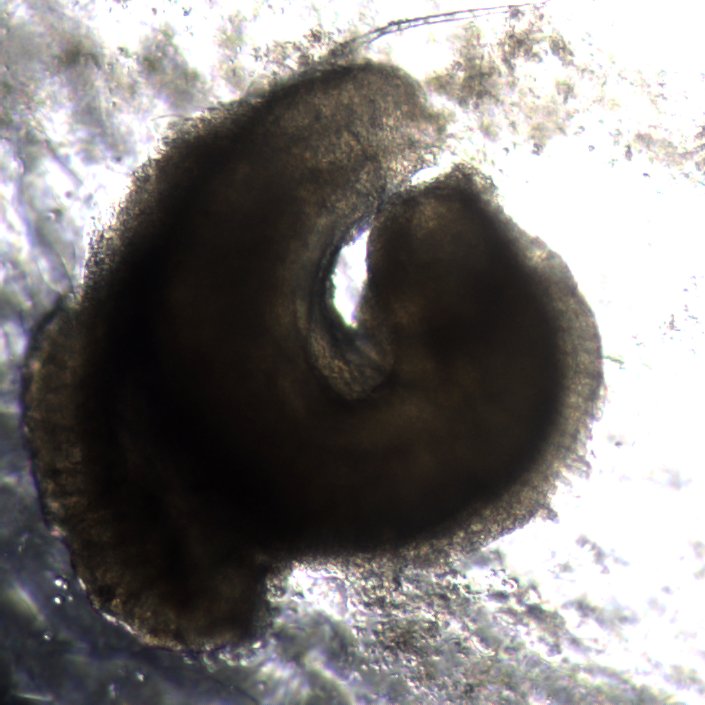

Supplement: Supplementary file 1 [file cells-11-00967-s001.zip › supplemetal videos/figure 3B mouse gut explant contraction JPEG time-lapse images/lobsterClaw524.jpg]

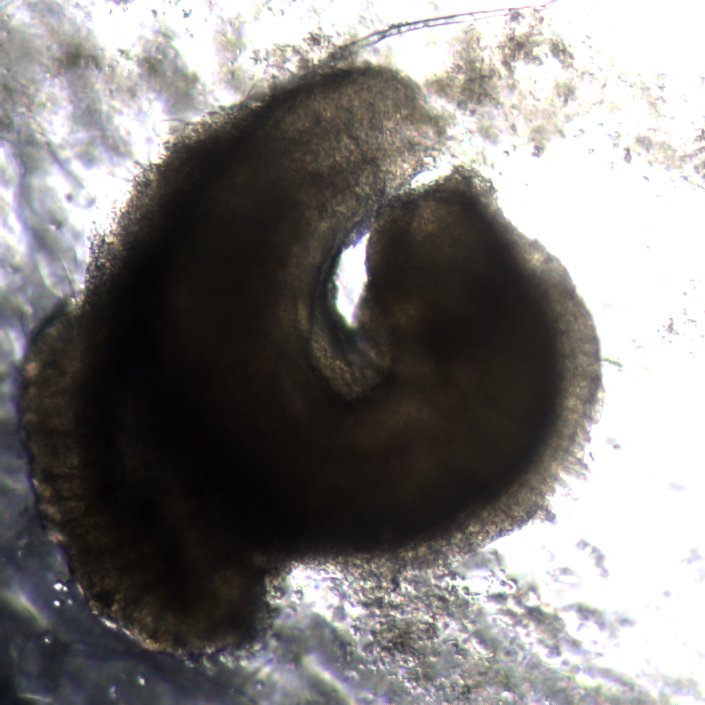

Supplement: Supplementary file 1 [file cells-11-00967-s001.zip › supplemetal videos/figure 3B mouse gut explant contraction JPEG time-lapse images/lobsterClaw242.jpg]

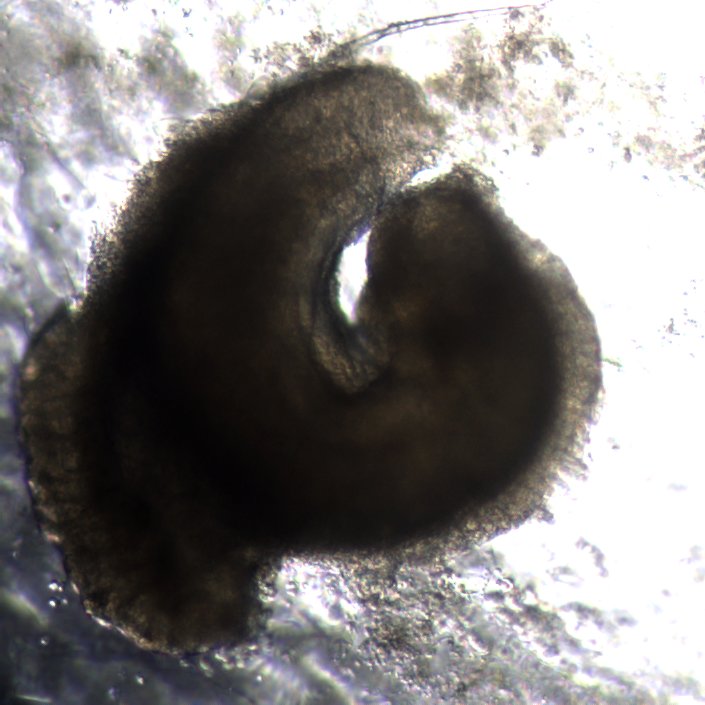

Supplement: Supplementary file 1 [file cells-11-00967-s001.zip › supplemetal videos/figure 3B mouse gut explant contraction JPEG time-lapse images/lobsterClaw295.jpg]

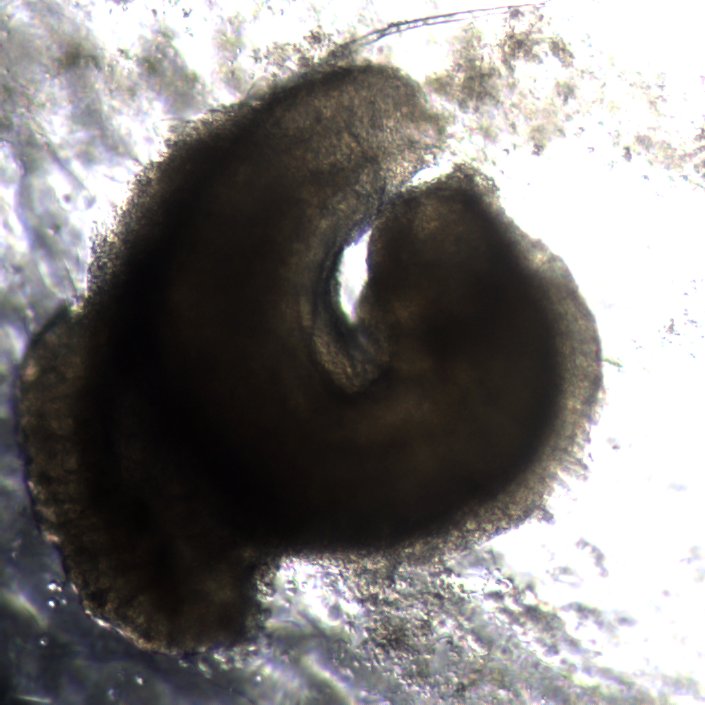

Supplement: Supplementary file 1 [file cells-11-00967-s001.zip › supplemetal videos/figure 3B mouse gut explant contraction JPEG time-lapse images/lobsterClaw281.jpg]

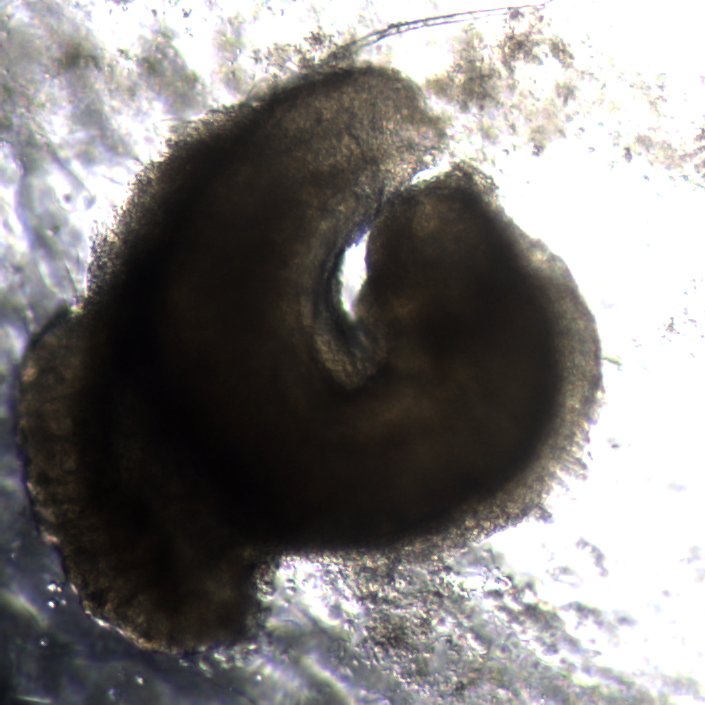

Supplement: Supplementary file 1 [file cells-11-00967-s001.zip › supplemetal videos/figure 3B mouse gut explant contraction JPEG time-lapse images/lobsterClaw280.jpg]

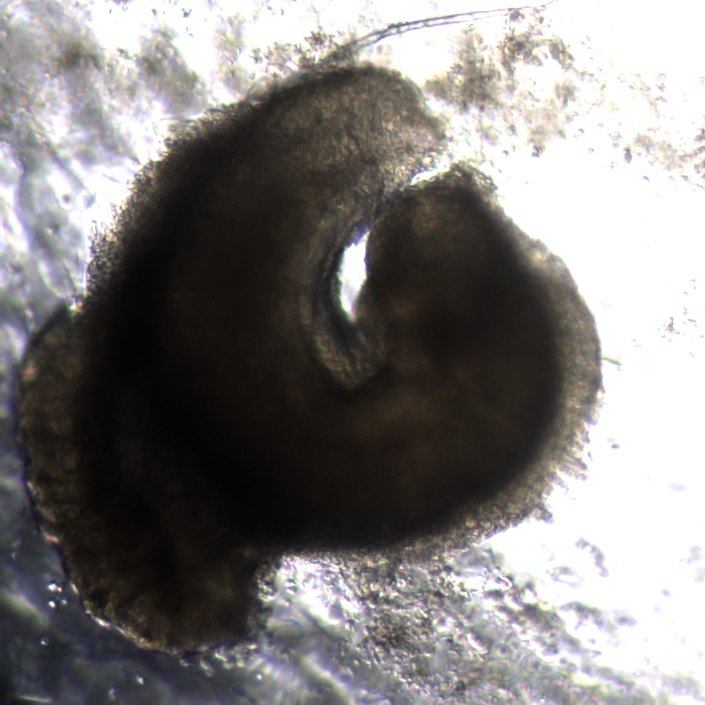

Supplement: Supplementary file 1 [file cells-11-00967-s001.zip › supplemetal videos/figure 3B mouse gut explant contraction JPEG time-lapse images/lobsterClaw294.jpg]

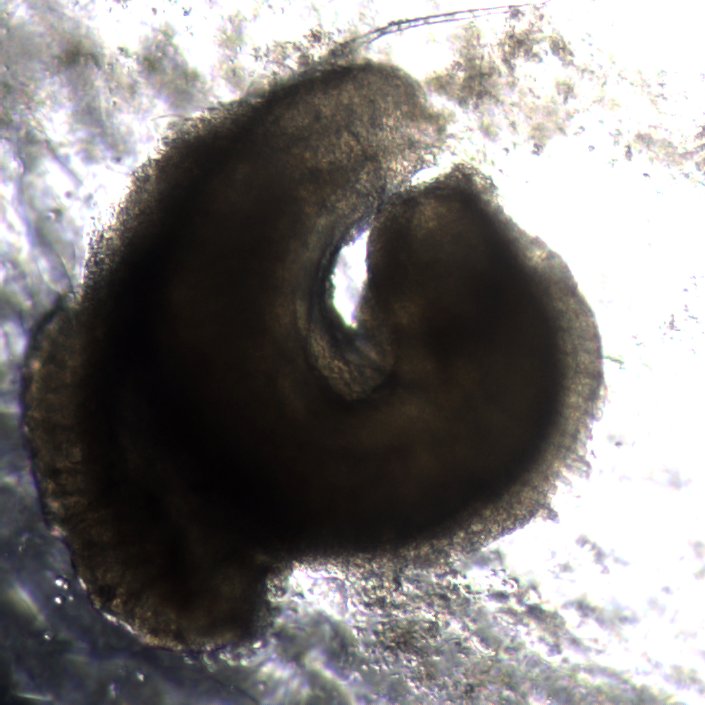

Supplement: Supplementary file 1 [file cells-11-00967-s001.zip › supplemetal videos/figure 3B mouse gut explant contraction JPEG time-lapse images/lobsterClaw525.jpg]

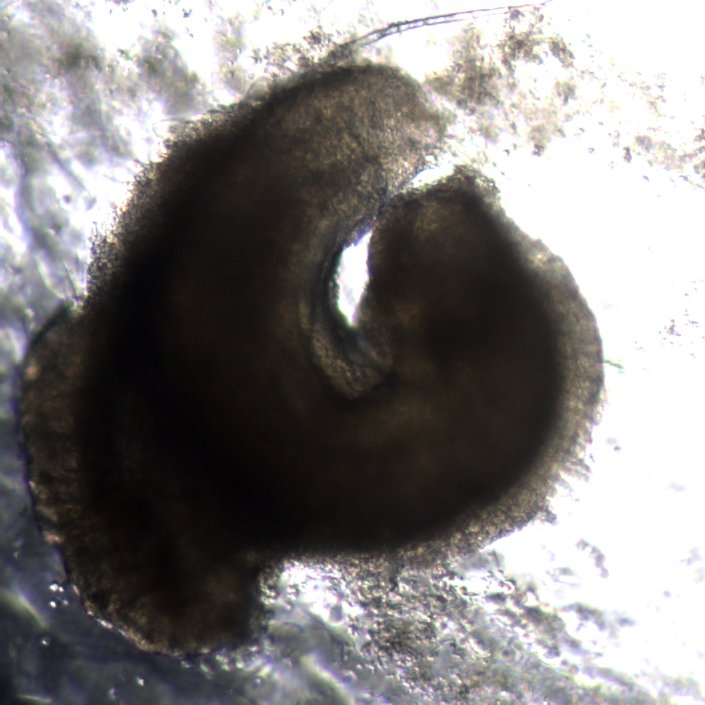

Supplement: Supplementary file 1 [file cells-11-00967-s001.zip › supplemetal videos/figure 3B mouse gut explant contraction JPEG time-lapse images/lobsterClaw243.jpg]

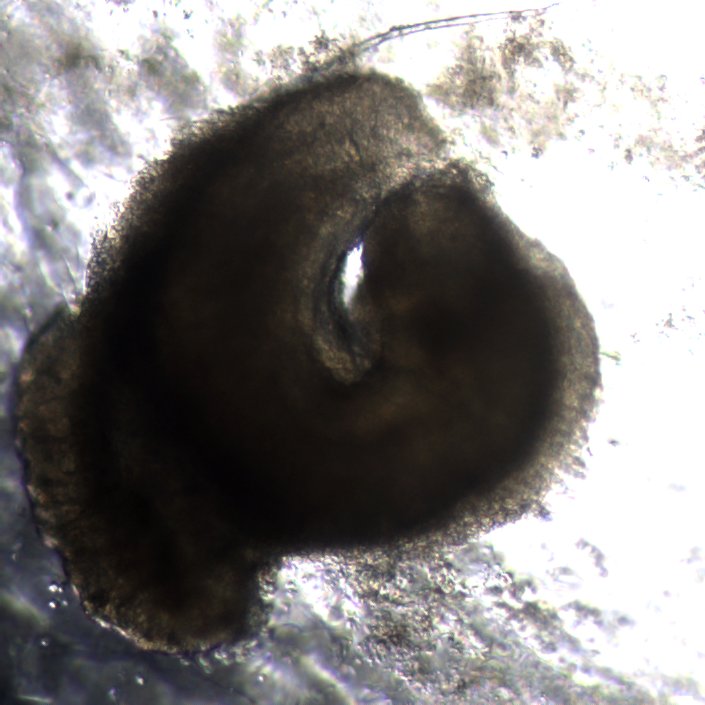

Supplement: Supplementary file 1 [file cells-11-00967-s001.zip › supplemetal videos/figure 3B mouse gut explant contraction JPEG time-lapse images/lobsterClaw257.jpg]

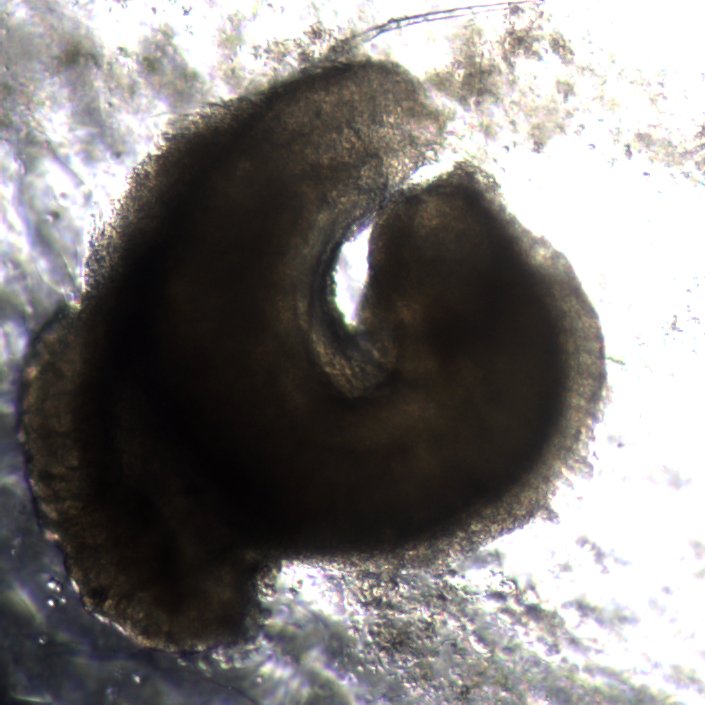

Supplement: Supplementary file 1 [file cells-11-00967-s001.zip › supplemetal videos/figure 3B mouse gut explant contraction JPEG time-lapse images/lobsterClaw531.jpg]

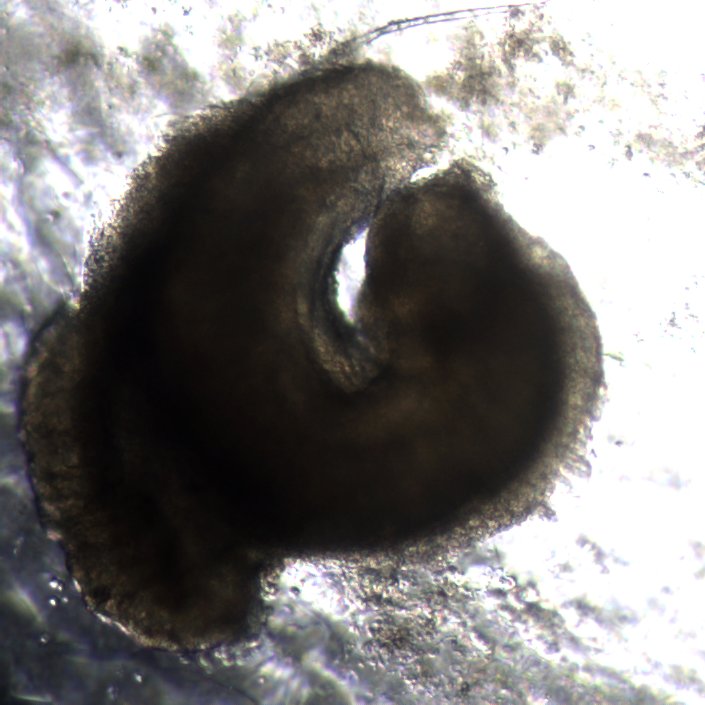

Supplement: Supplementary file 1 [file cells-11-00967-s001.zip › supplemetal videos/figure 3B mouse gut explant contraction JPEG time-lapse images/lobsterClaw519.jpg]

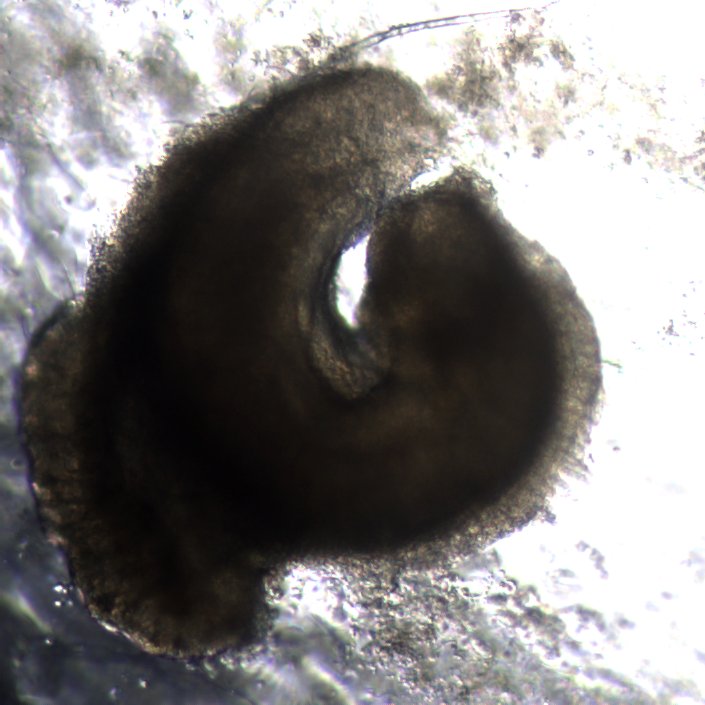

Supplement: Supplementary file 1 [file cells-11-00967-s001.zip › supplemetal videos/figure 3B mouse gut explant contraction JPEG time-lapse images/lobsterClaw082.jpg]

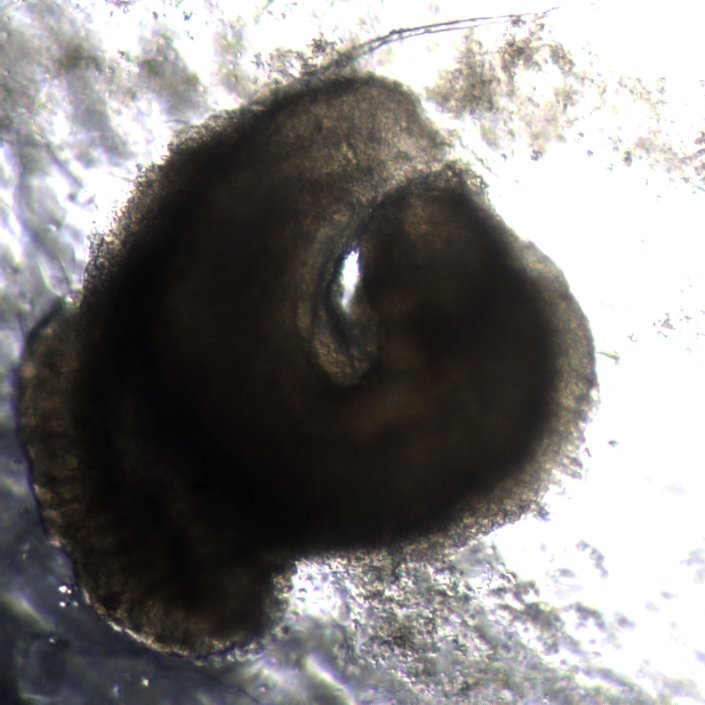

Supplement: Supplementary file 1 [file cells-11-00967-s001.zip › supplemetal videos/figure 3B mouse gut explant contraction JPEG time-lapse images/lobsterClaw096.jpg]

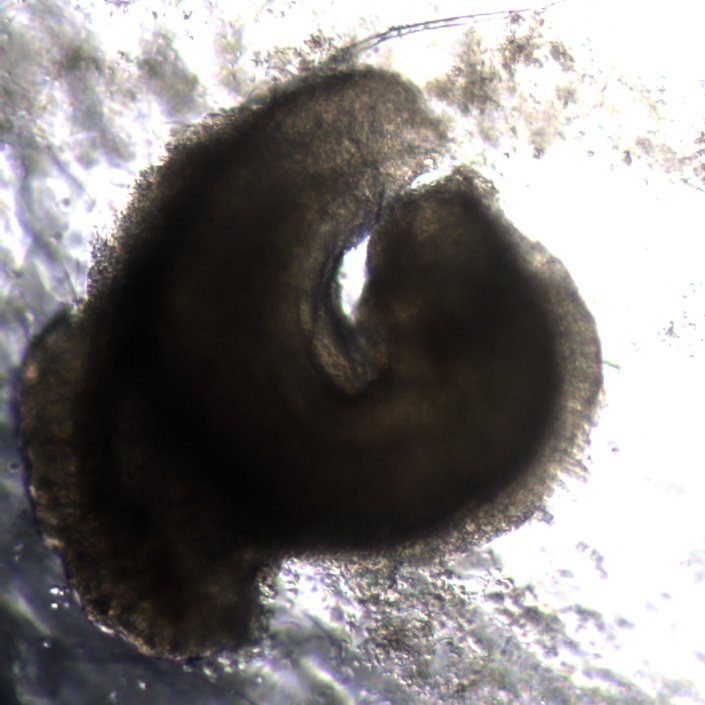

Supplement: Supplementary file 1 [file cells-11-00967-s001.zip › supplemetal videos/figure 3B mouse gut explant contraction JPEG time-lapse images/lobsterClaw041.jpg]

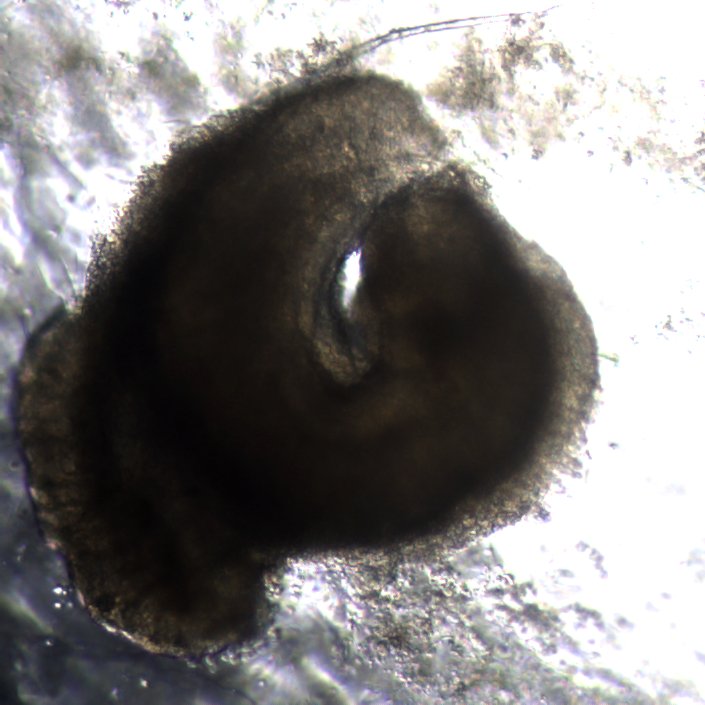

Supplement: Supplementary file 1 [file cells-11-00967-s001.zip › supplemetal videos/figure 3B mouse gut explant contraction JPEG time-lapse images/lobsterClaw055.jpg]

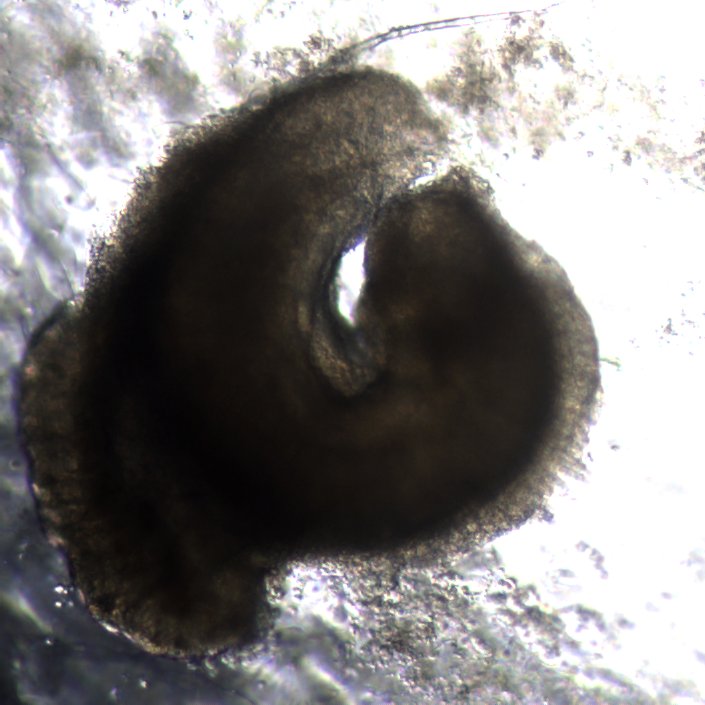

Supplement: Supplementary file 1 [file cells-11-00967-s001.zip › supplemetal videos/figure 3B mouse gut explant contraction JPEG time-lapse images/lobsterClaw069.jpg]

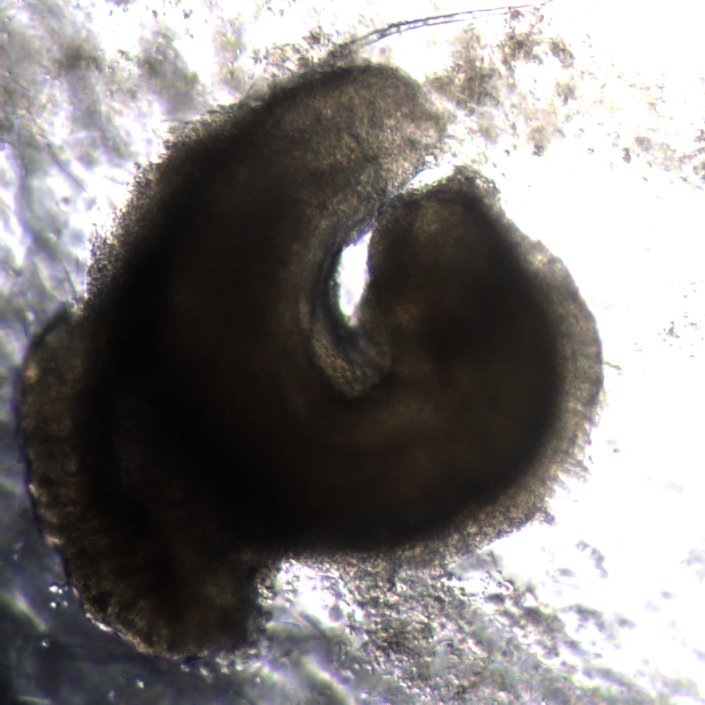

Supplement: Supplementary file 1 [file cells-11-00967-s001.zip › supplemetal videos/figure 3B mouse gut explant contraction JPEG time-lapse images/lobsterClaw135.jpg]

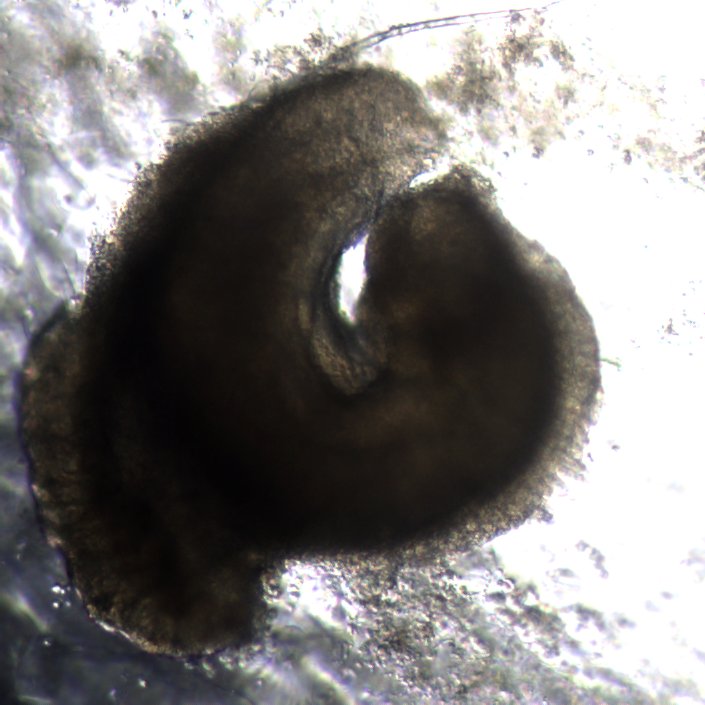

Supplement: Supplementary file 1 [file cells-11-00967-s001.zip › supplemetal videos/figure 3B mouse gut explant contraction JPEG time-lapse images/lobsterClaw121.jpg]

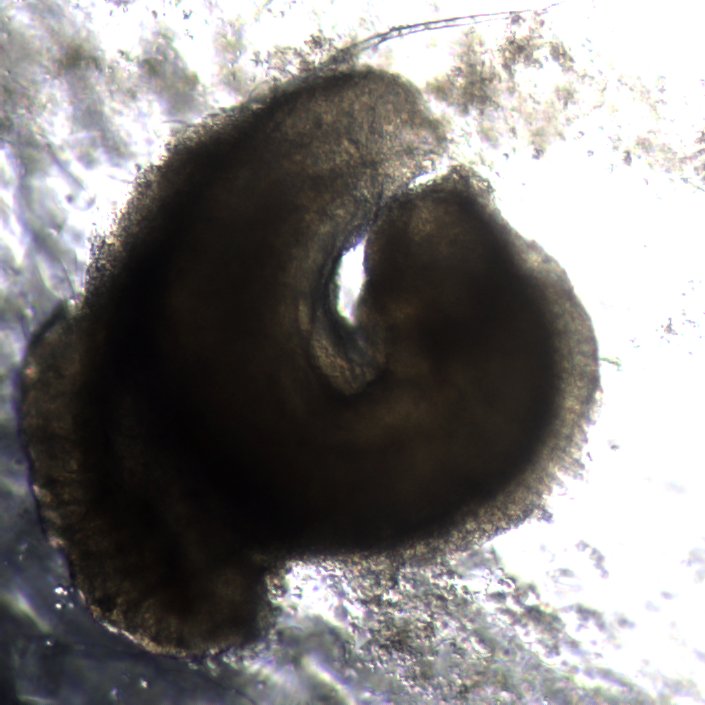

Supplement: Supplementary file 1 [file cells-11-00967-s001.zip › supplemetal videos/figure 3B mouse gut explant contraction JPEG time-lapse images/lobsterClaw109.jpg]

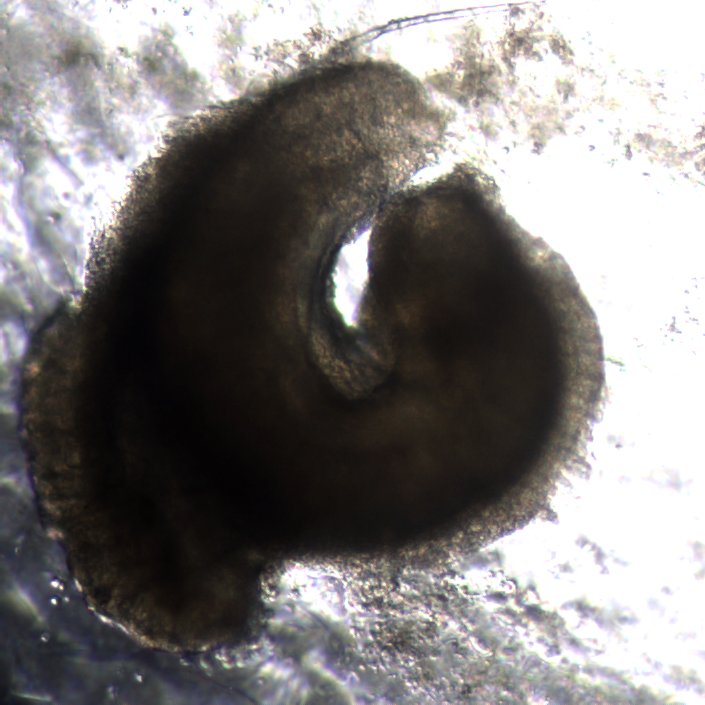

Supplement: Supplementary file 1 [file cells-11-00967-s001.zip › supplemetal videos/figure 3B mouse gut explant contraction JPEG time-lapse images/lobsterClaw492.jpg]

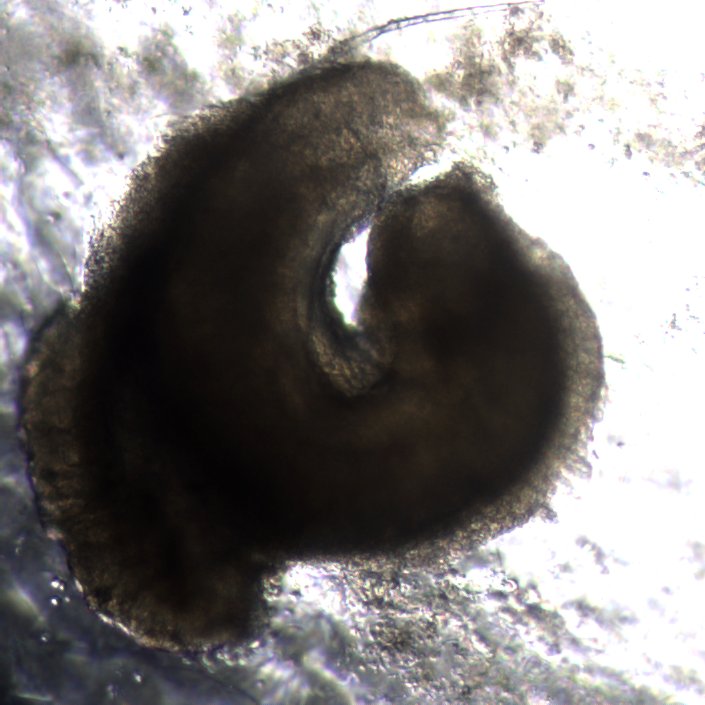

Supplement: Supplementary file 1 [file cells-11-00967-s001.zip › supplemetal videos/figure 3B mouse gut explant contraction JPEG time-lapse images/lobsterClaw486.jpg]

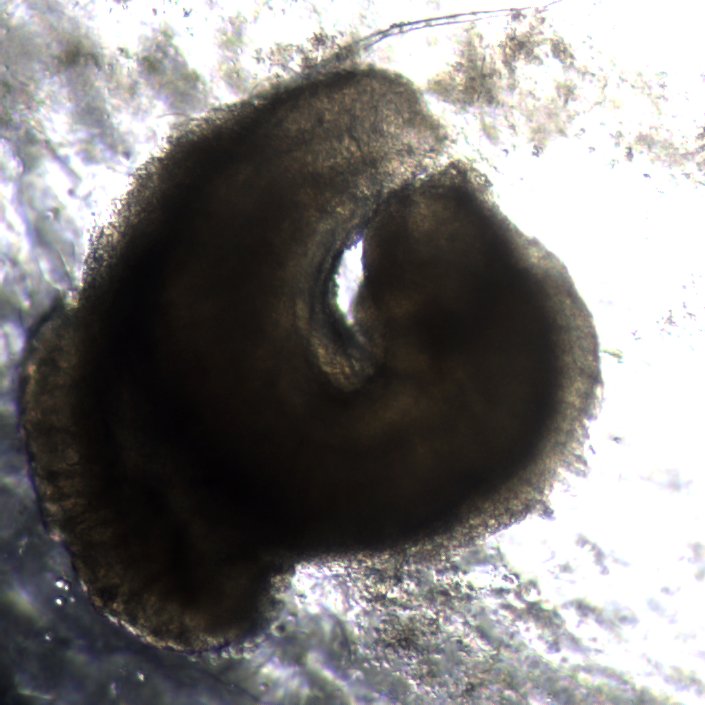

Supplement: Supplementary file 1 [file cells-11-00967-s001.zip › supplemetal videos/figure 3B mouse gut explant contraction JPEG time-lapse images/lobsterClaw451.jpg]

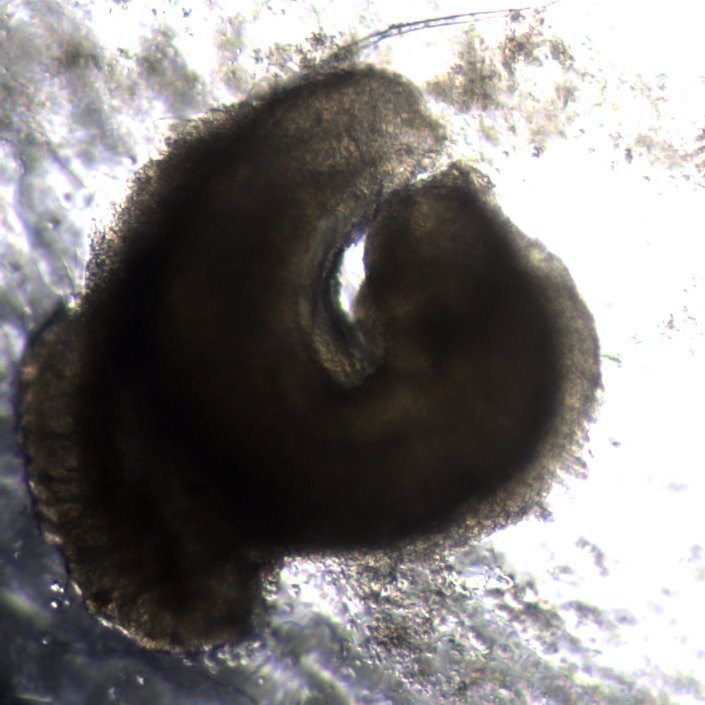

Supplement: Supplementary file 1 [file cells-11-00967-s001.zip › supplemetal videos/figure 3B mouse gut explant contraction JPEG time-lapse images/lobsterClaw337.jpg]

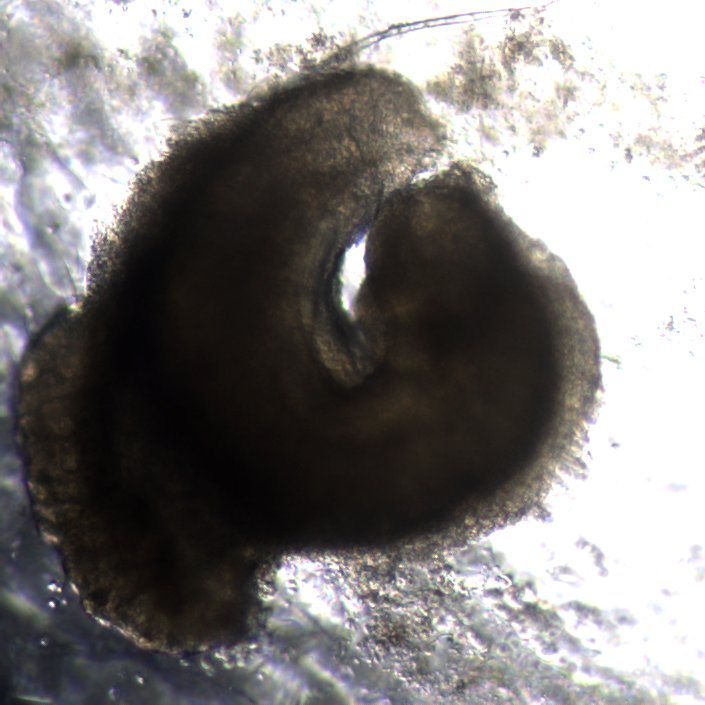

Supplement: Supplementary file 1 [file cells-11-00967-s001.zip › supplemetal videos/figure 3B mouse gut explant contraction JPEG time-lapse images/lobsterClaw323.jpg]

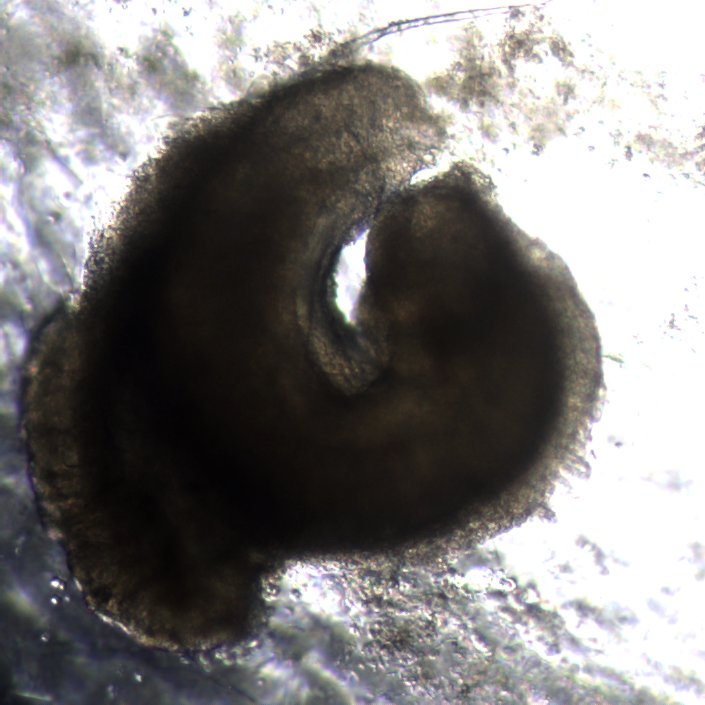

Supplement: Supplementary file 1 [file cells-11-00967-s001.zip › supplemetal videos/figure 3B mouse gut explant contraction JPEG time-lapse images/lobsterClaw445.jpg]

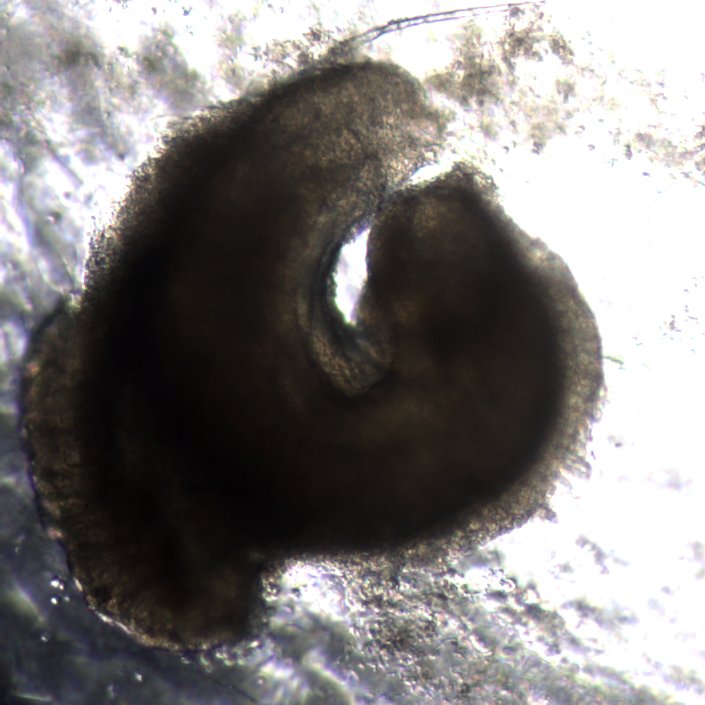

Supplement: Supplementary file 1 [file cells-11-00967-s001.zip › supplemetal videos/figure 3B mouse gut explant contraction JPEG time-lapse images/lobsterClaw479.jpg]

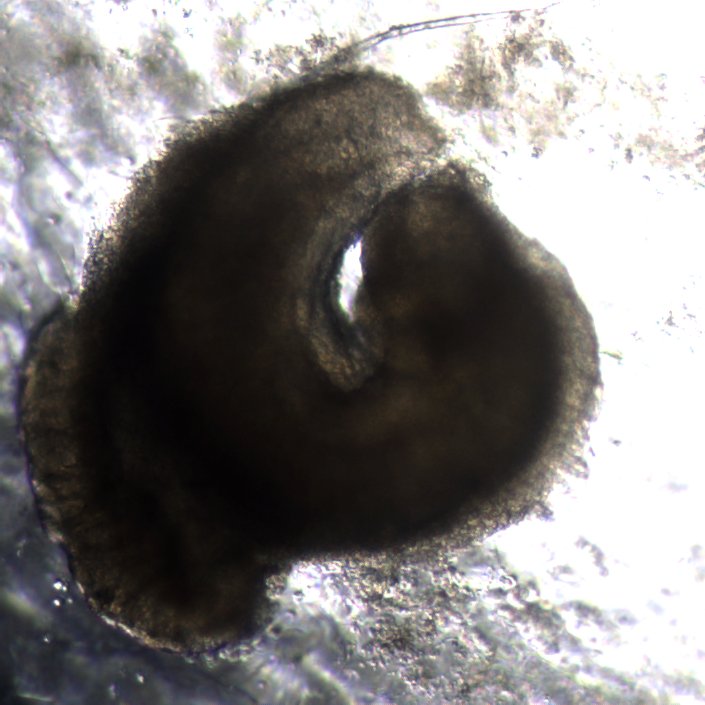

Supplement: Supplementary file 1 [file cells-11-00967-s001.zip › supplemetal videos/figure 3B mouse gut explant contraction JPEG time-lapse images/lobsterClaw344.jpg]

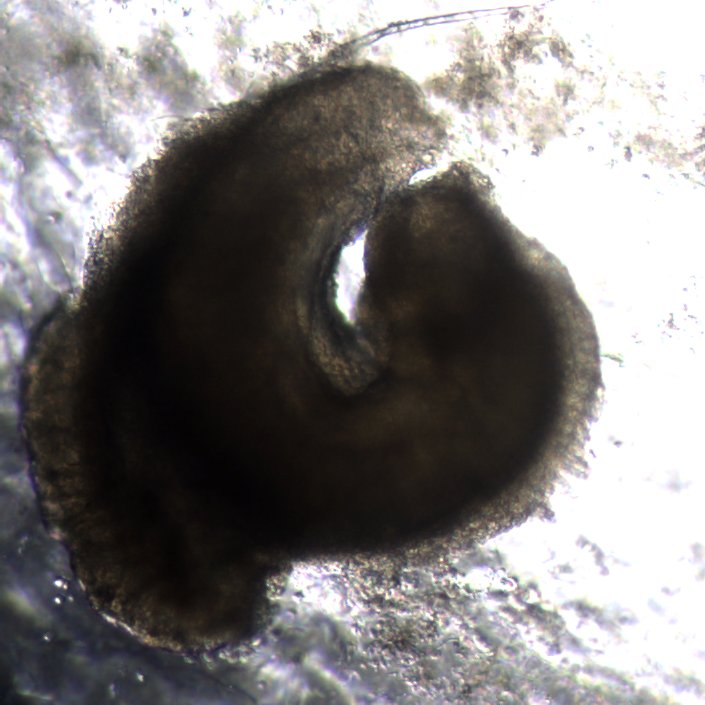

Supplement: Supplementary file 1 [file cells-11-00967-s001.zip › supplemetal videos/figure 3B mouse gut explant contraction JPEG time-lapse images/lobsterClaw422.jpg]

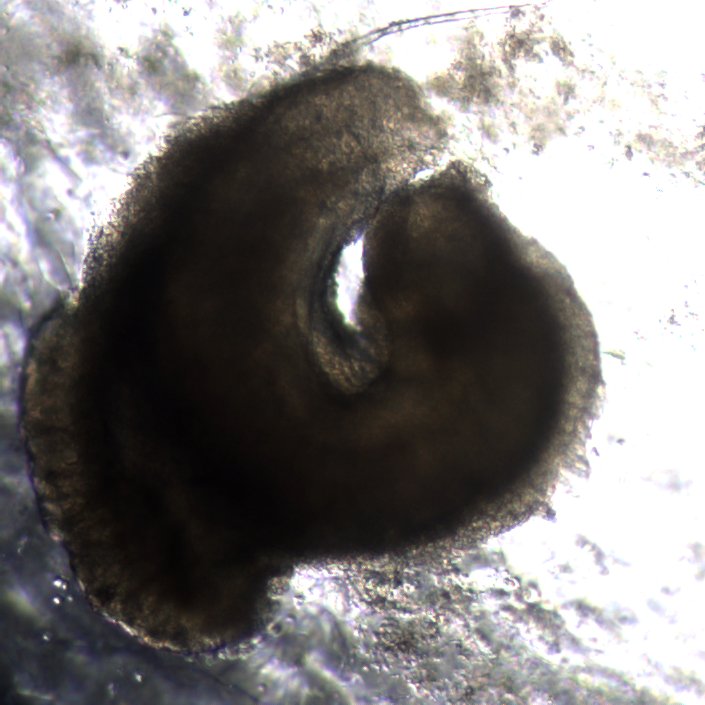

Supplement: Supplementary file 1 [file cells-11-00967-s001.zip › supplemetal videos/figure 3B mouse gut explant contraction JPEG time-lapse images/lobsterClaw436.jpg]

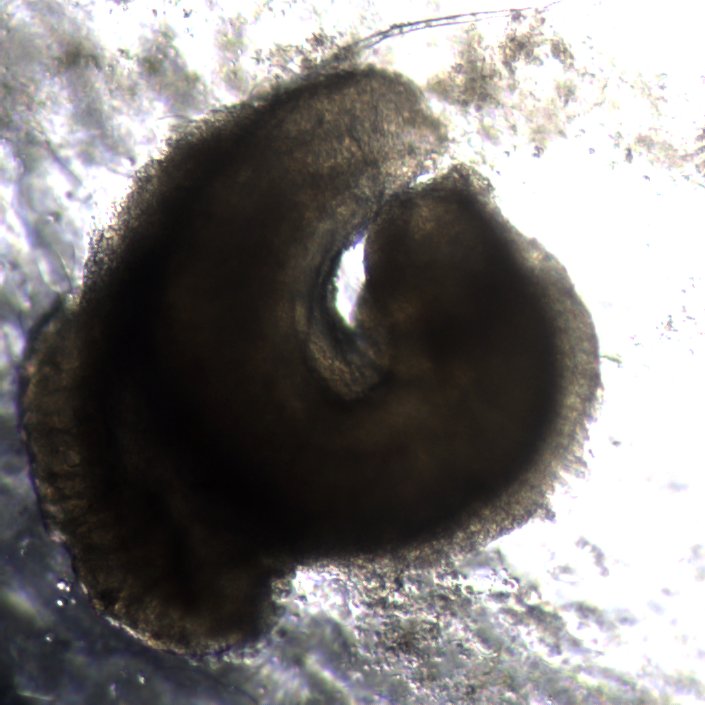

Supplement: Supplementary file 1 [file cells-11-00967-s001.zip › supplemetal videos/figure 3B mouse gut explant contraction JPEG time-lapse images/lobsterClaw350.jpg]

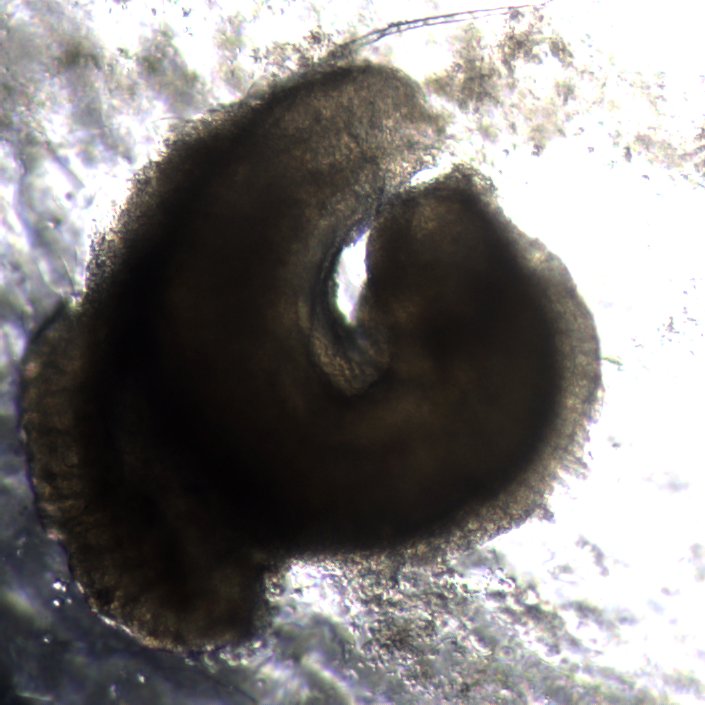

Supplement: Supplementary file 1 [file cells-11-00967-s001.zip › supplemetal videos/figure 3B mouse gut explant contraction JPEG time-lapse images/lobsterClaw378.jpg]

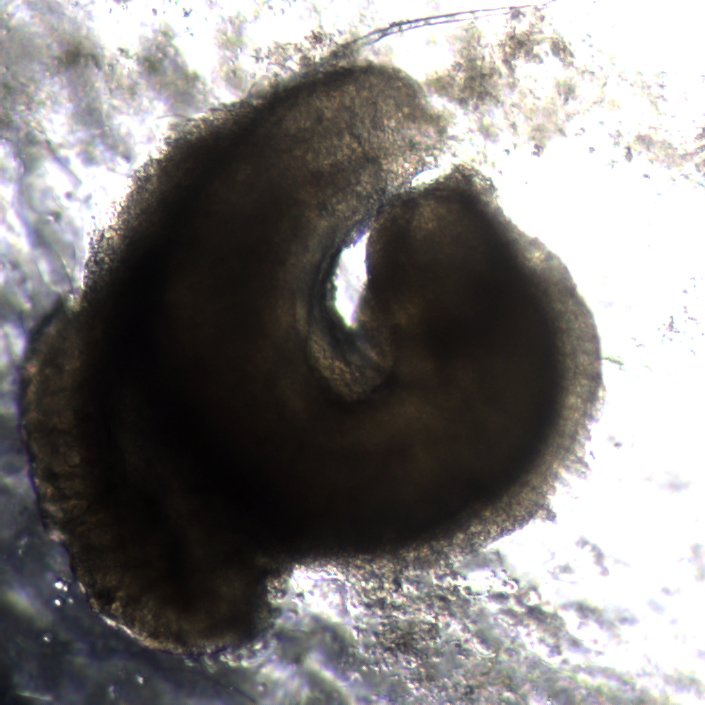

Supplement: Supplementary file 1 [file cells-11-00967-s001.zip › supplemetal videos/figure 3B mouse gut explant contraction JPEG time-lapse images/lobsterClaw387.jpg]

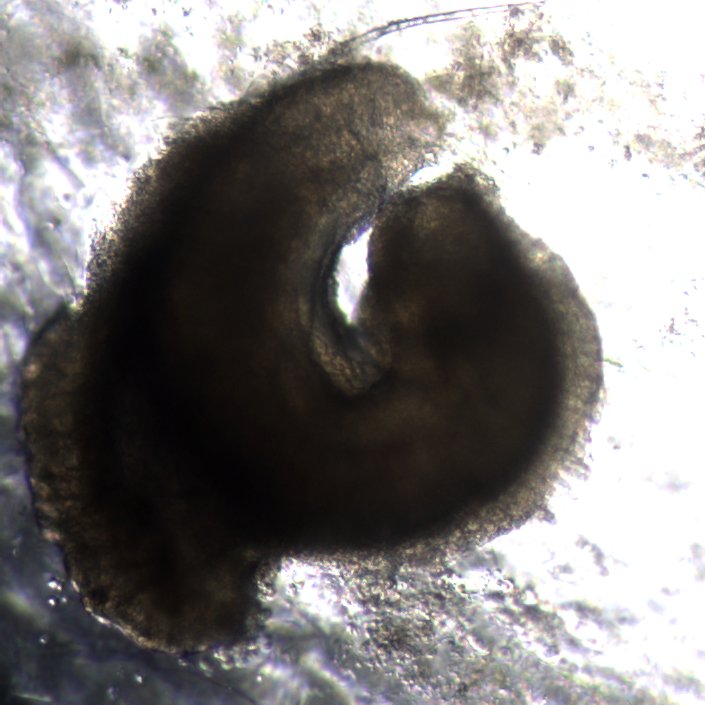

Supplement: Supplementary file 1 [file cells-11-00967-s001.zip › supplemetal videos/figure 3B mouse gut explant contraction JPEG time-lapse images/lobsterClaw393.jpg]

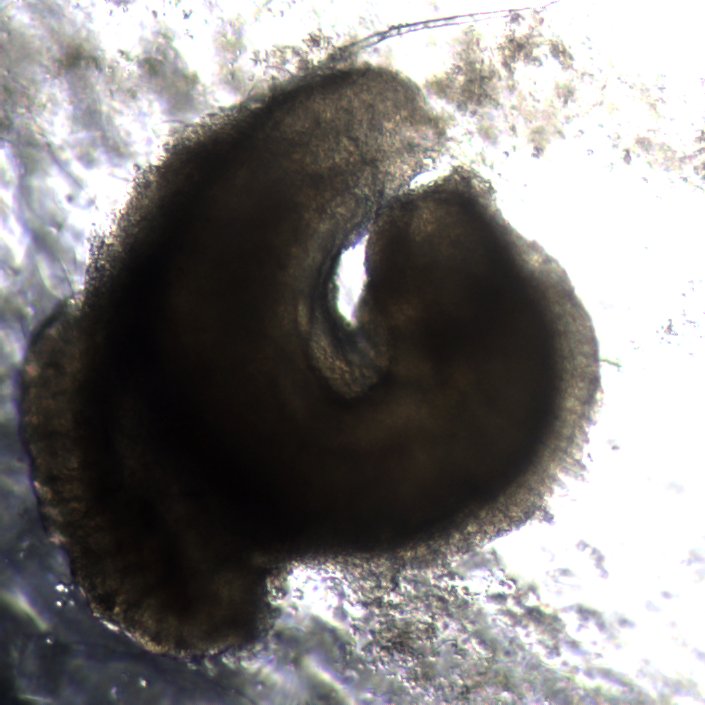

Supplement: Supplementary file 1 [file cells-11-00967-s001.zip › supplemetal videos/figure 3B mouse gut explant contraction JPEG time-lapse images/lobsterClaw146.jpg]

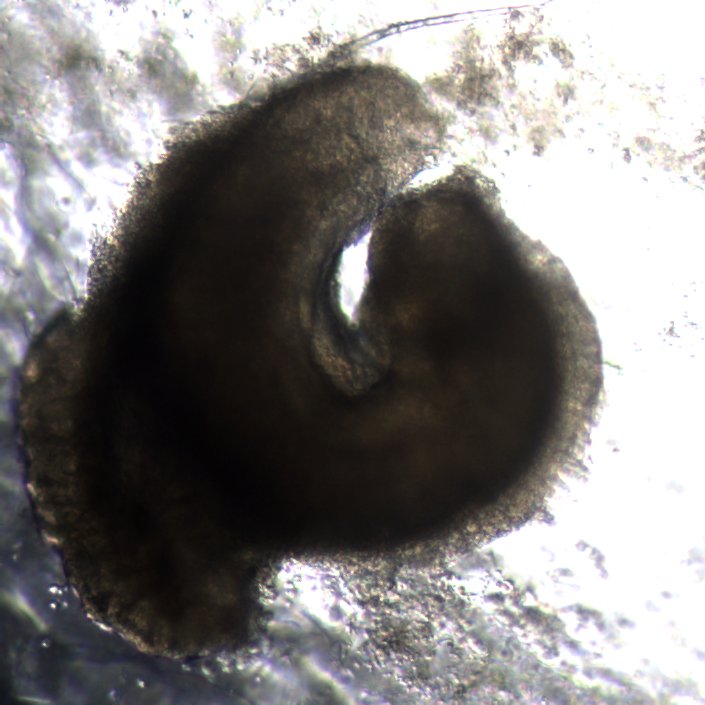

Supplement: Supplementary file 1 [file cells-11-00967-s001.zip › supplemetal videos/figure 3B mouse gut explant contraction JPEG time-lapse images/lobsterClaw152.jpg]

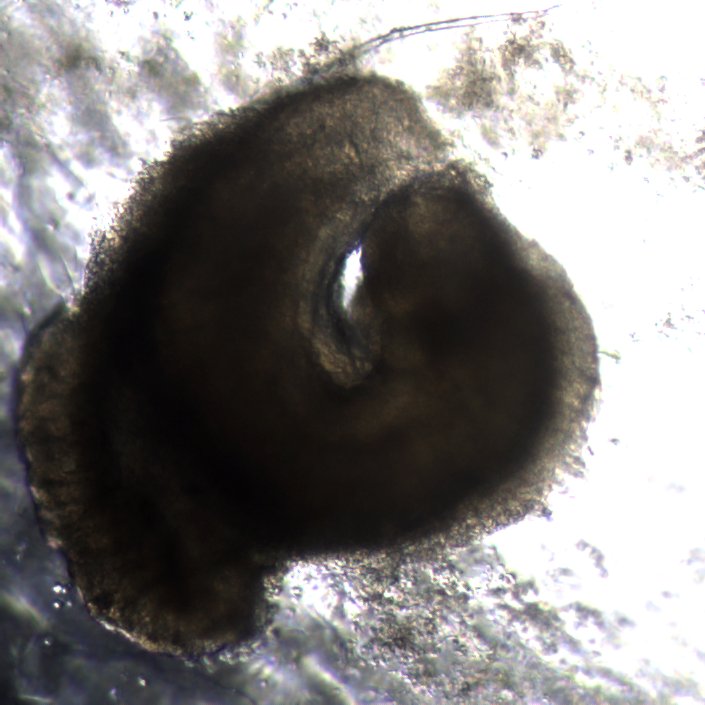

Supplement: Supplementary file 1 [file cells-11-00967-s001.zip › supplemetal videos/figure 3B mouse gut explant contraction JPEG time-lapse images/lobsterClaw185.jpg]

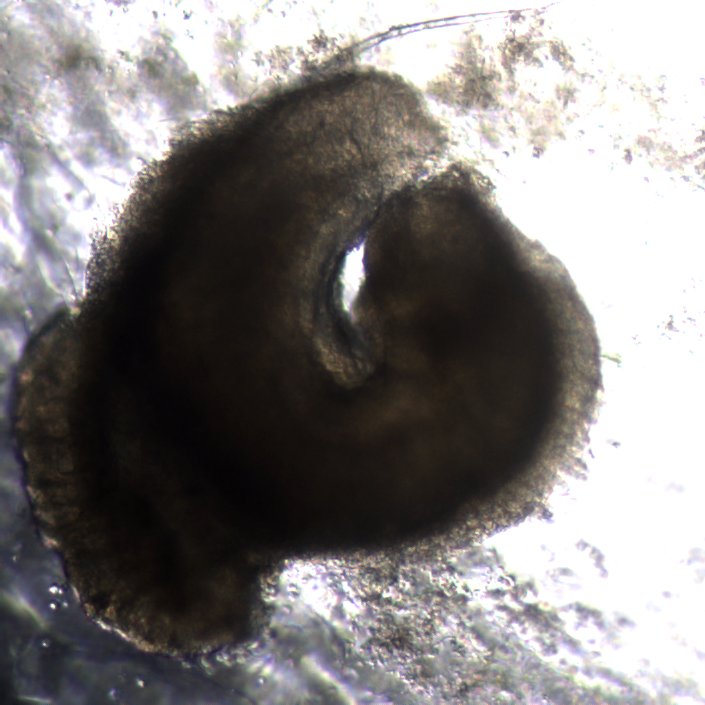

Supplement: Supplementary file 1 [file cells-11-00967-s001.zip › supplemetal videos/figure 3B mouse gut explant contraction JPEG time-lapse images/lobsterClaw191.jpg]

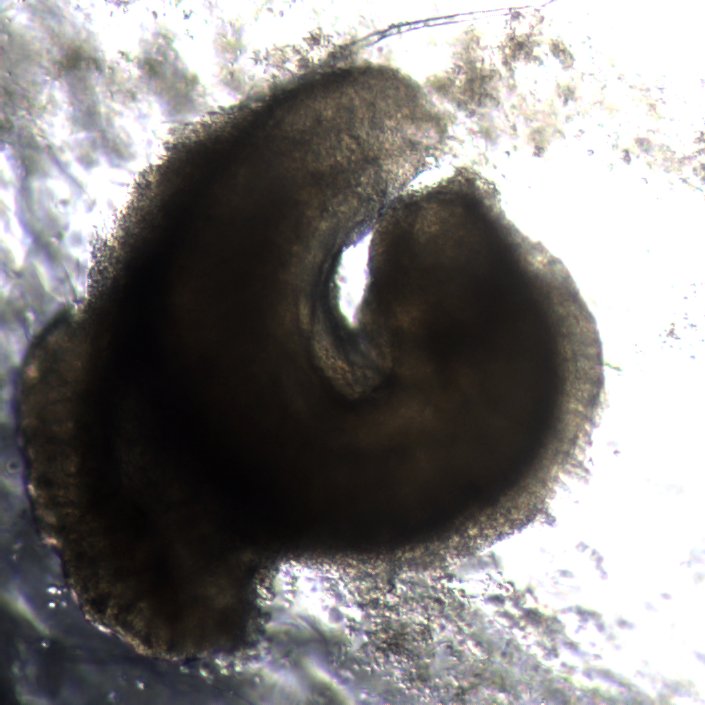

Supplement: Supplementary file 1 [file cells-11-00967-s001.zip › supplemetal videos/figure 3B mouse gut explant contraction JPEG time-lapse images/lobsterClaw032.jpg]

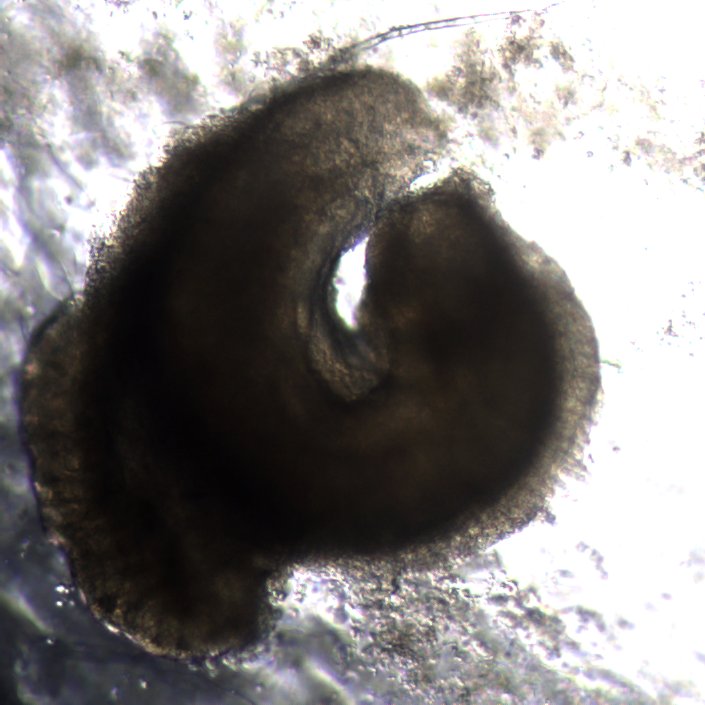

Supplement: Supplementary file 1 [file cells-11-00967-s001.zip › supplemetal videos/figure 3B mouse gut explant contraction JPEG time-lapse images/lobsterClaw026.jpg]

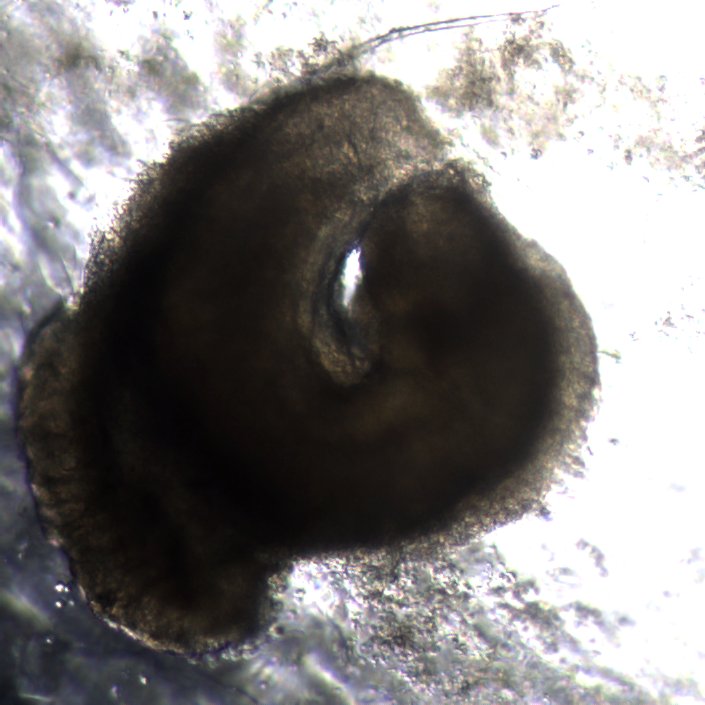

Supplement: Supplementary file 1 [file cells-11-00967-s001.zip › supplemetal videos/figure 3B mouse gut explant contraction JPEG time-lapse images/lobsterClaw230.jpg]

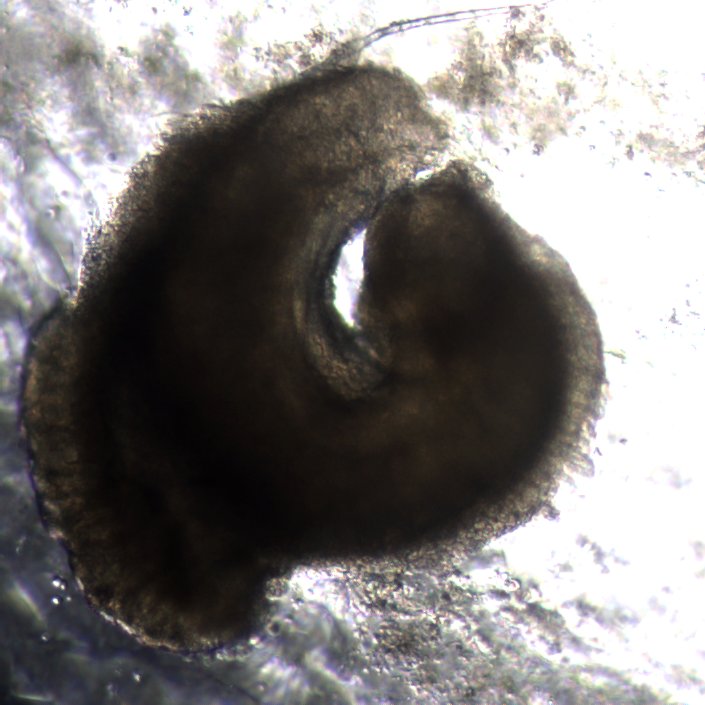

Supplement: Supplementary file 1 [file cells-11-00967-s001.zip › supplemetal videos/figure 3B mouse gut explant contraction JPEG time-lapse images/lobsterClaw556.jpg]

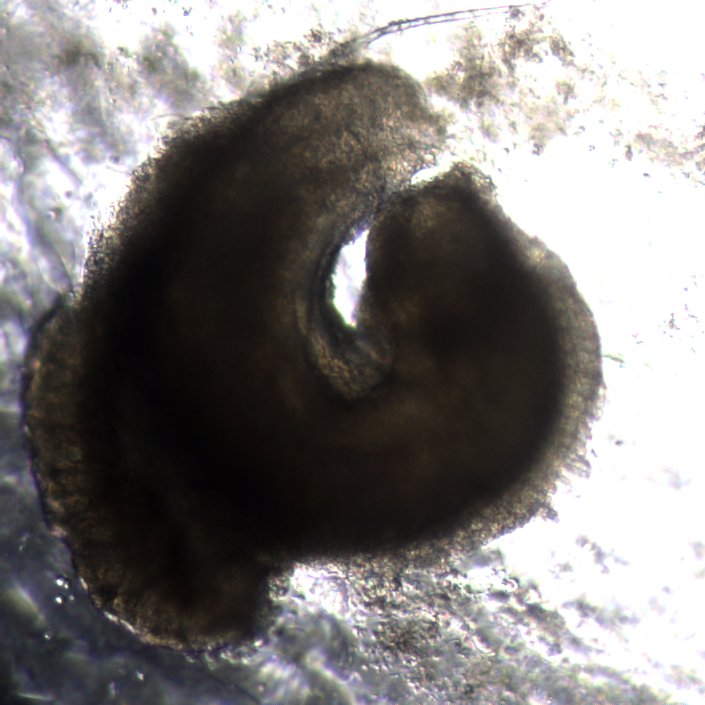

Supplement: Supplementary file 1 [file cells-11-00967-s001.zip › supplemetal videos/figure 3B mouse gut explant contraction JPEG time-lapse images/lobsterClaw542.jpg]

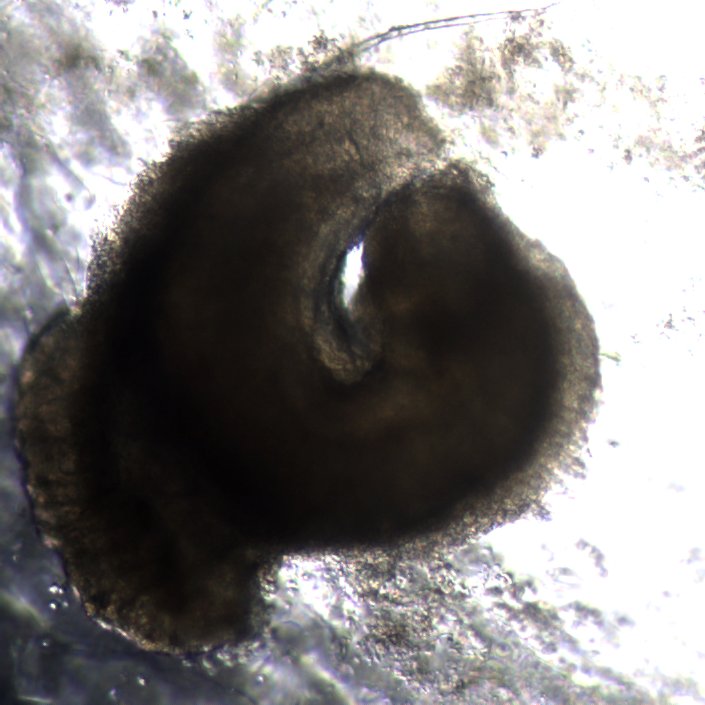

Supplement: Supplementary file 1 [file cells-11-00967-s001.zip › supplemetal videos/figure 3B mouse gut explant contraction JPEG time-lapse images/lobsterClaw224.jpg]

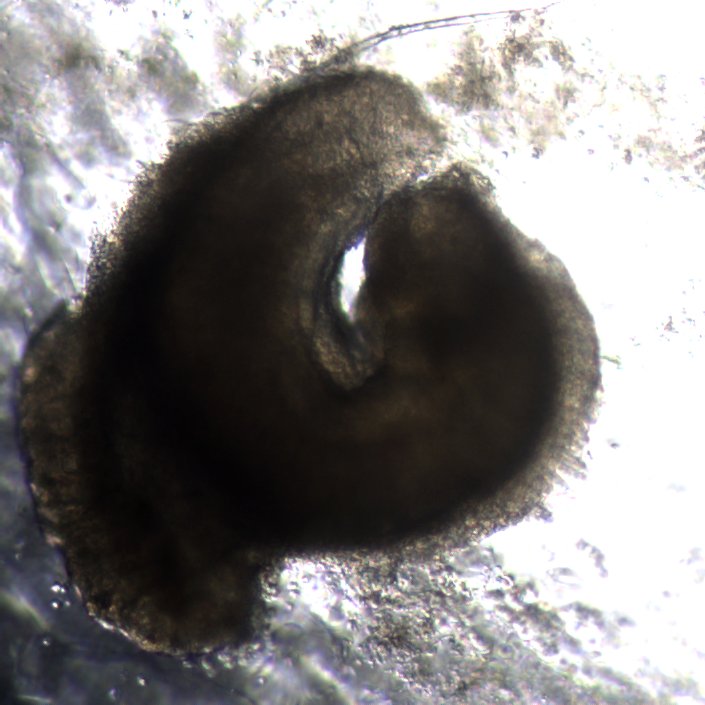

Supplement: Supplementary file 1 [file cells-11-00967-s001.zip › supplemetal videos/figure 3B mouse gut explant contraction JPEG time-lapse images/lobsterClaw218.jpg]

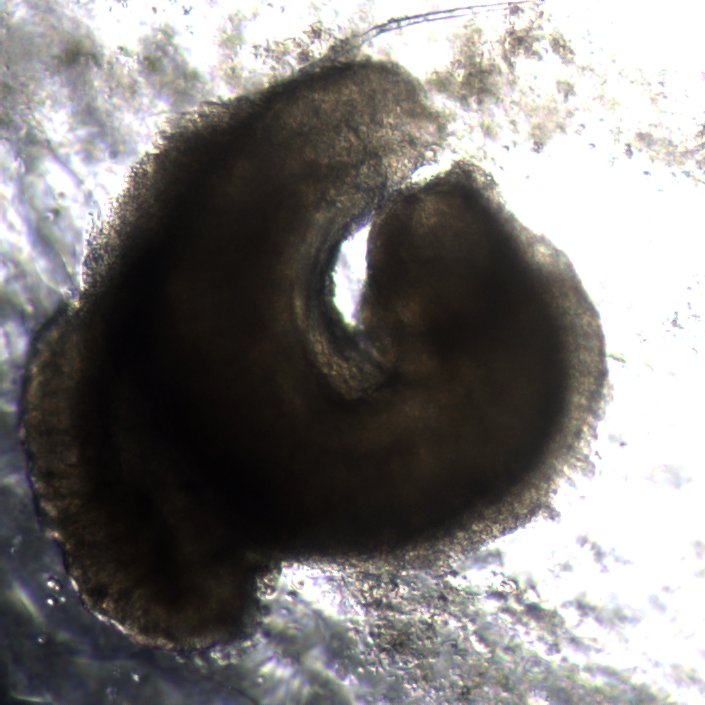

Supplement: Supplementary file 1 [file cells-11-00967-s001.zip › supplemetal videos/figure 3B mouse gut explant contraction JPEG time-lapse images/lobsterClaw595.jpg]

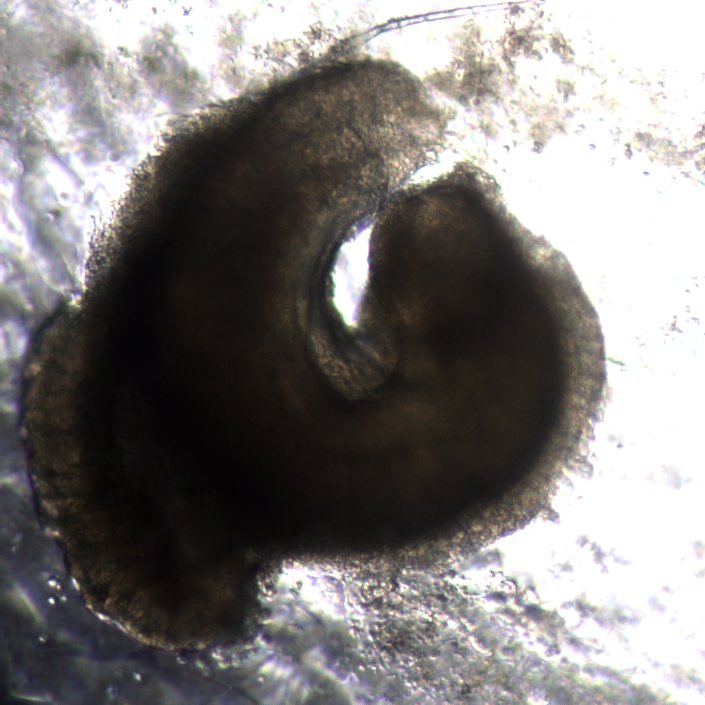

Supplement: Supplementary file 1 [file cells-11-00967-s001.zip › supplemetal videos/figure 3B mouse gut explant contraction JPEG time-lapse images/lobsterClaw581.jpg]

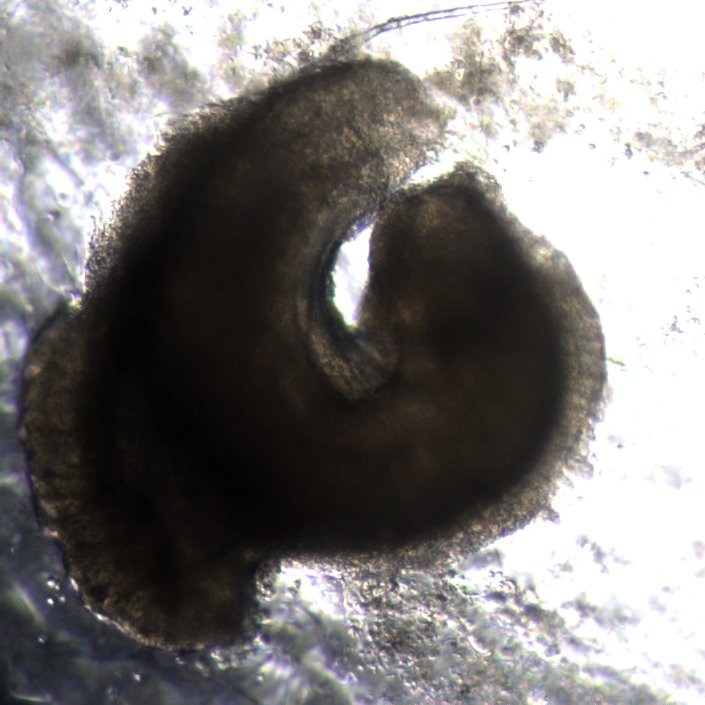

Supplement: Supplementary file 1 [file cells-11-00967-s001.zip › supplemetal videos/figure 3B mouse gut explant contraction JPEG time-lapse images/lobsterClaw580.jpg]

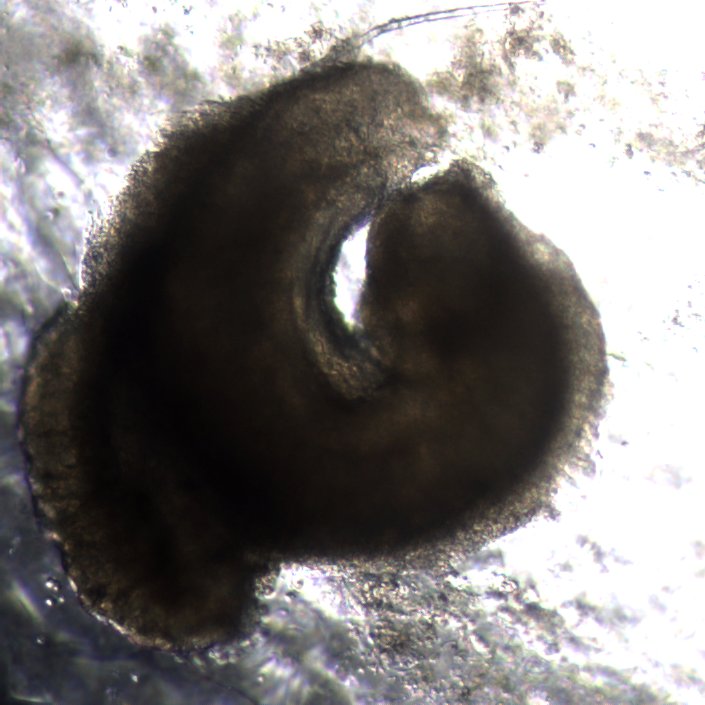

Supplement: Supplementary file 1 [file cells-11-00967-s001.zip › supplemetal videos/figure 3B mouse gut explant contraction JPEG time-lapse images/lobsterClaw594.jpg]

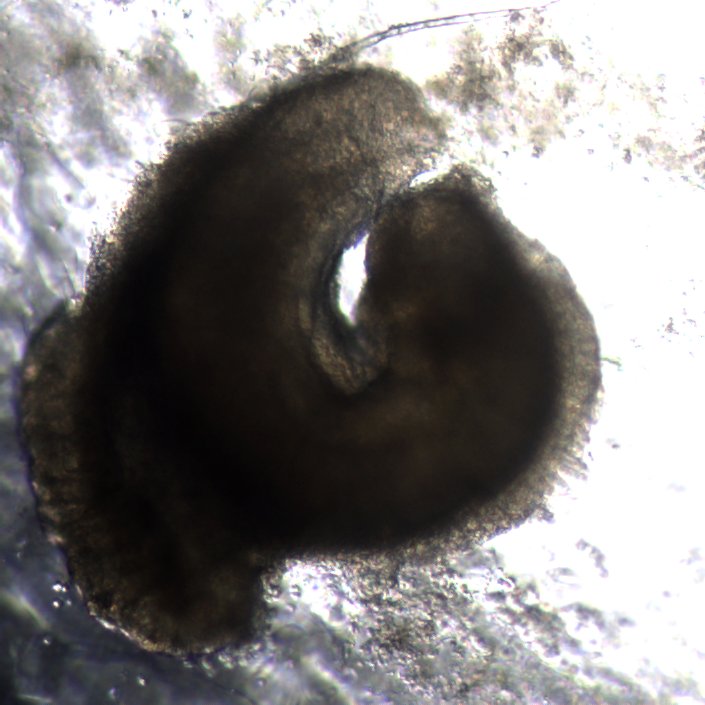

Supplement: Supplementary file 1 [file cells-11-00967-s001.zip › supplemetal videos/figure 3B mouse gut explant contraction JPEG time-lapse images/lobsterClaw219.jpg]

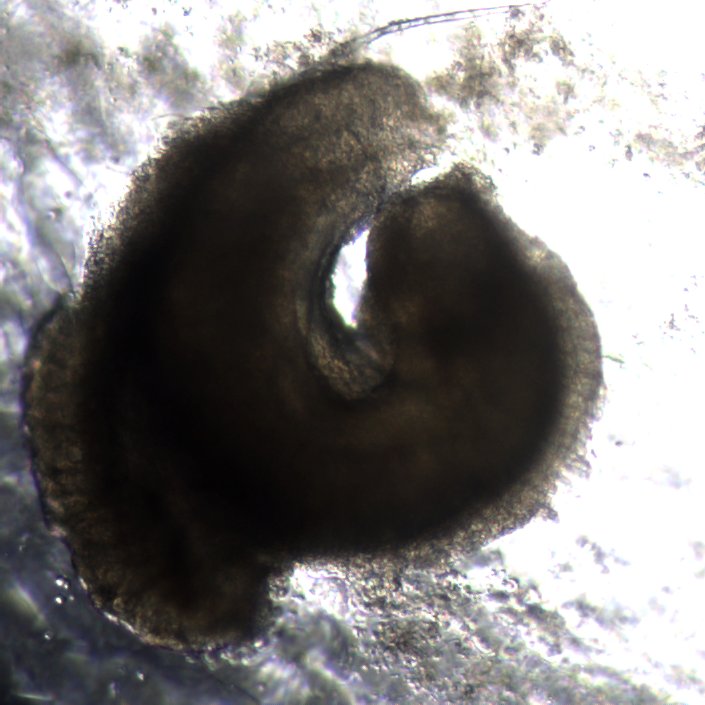

Supplement: Supplementary file 1 [file cells-11-00967-s001.zip › supplemetal videos/figure 3B mouse gut explant contraction JPEG time-lapse images/lobsterClaw543.jpg]

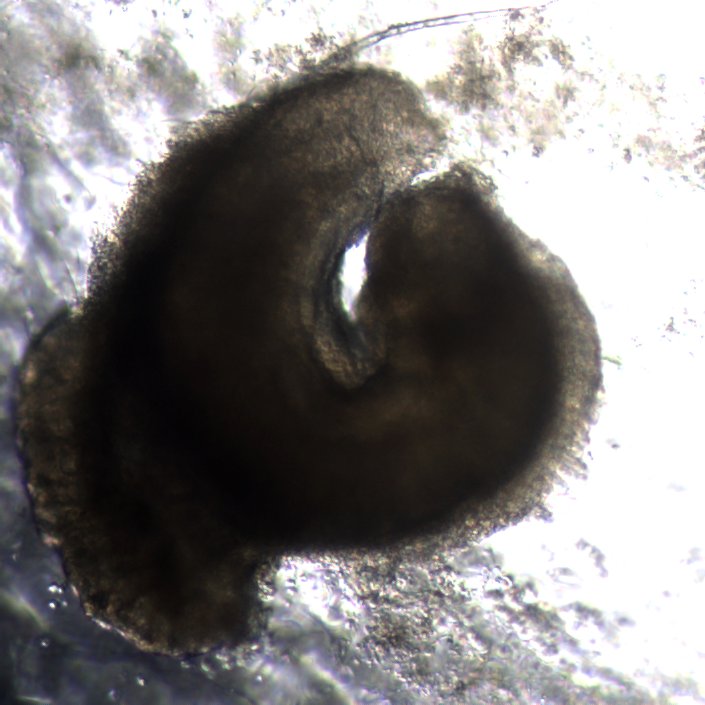

Supplement: Supplementary file 1 [file cells-11-00967-s001.zip › supplemetal videos/figure 3B mouse gut explant contraction JPEG time-lapse images/lobsterClaw225.jpg]

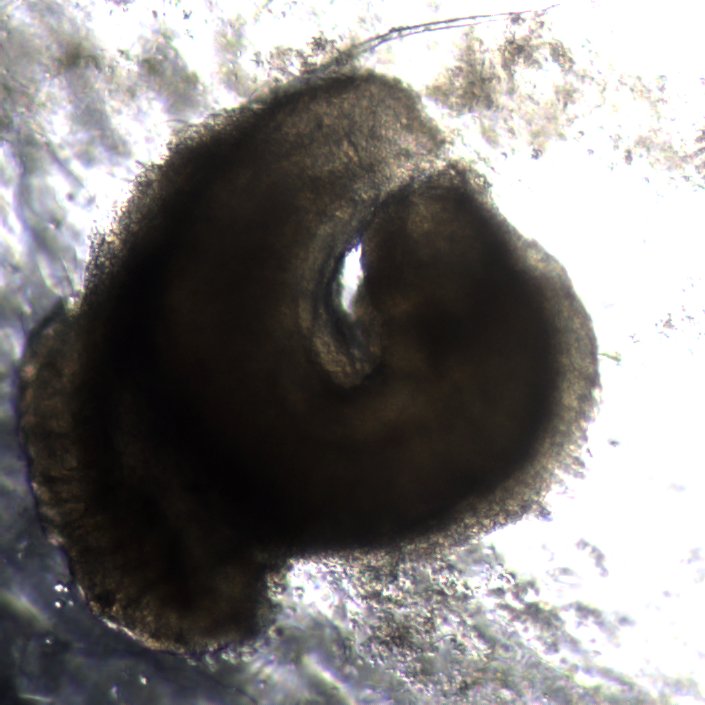

Supplement: Supplementary file 1 [file cells-11-00967-s001.zip › supplemetal videos/figure 3B mouse gut explant contraction JPEG time-lapse images/lobsterClaw231.jpg]

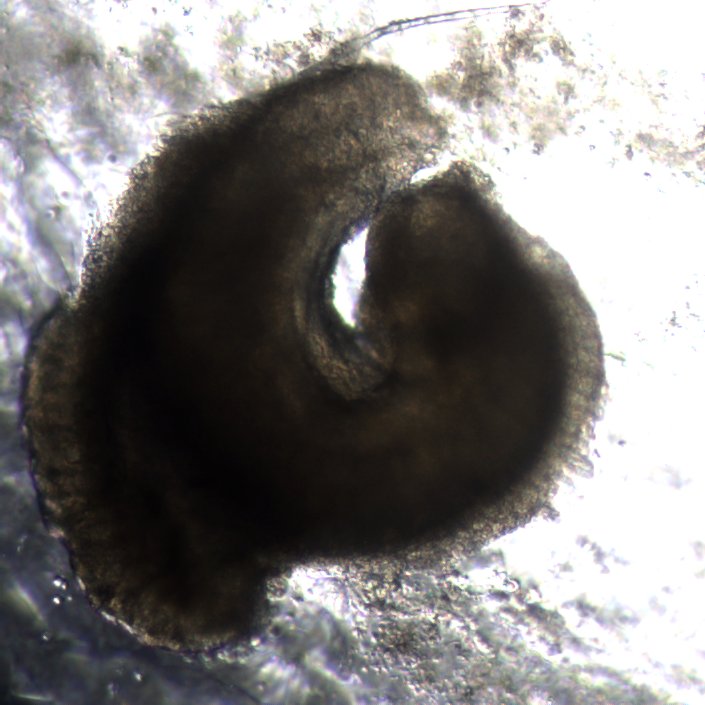

Supplement: Supplementary file 1 [file cells-11-00967-s001.zip › supplemetal videos/figure 3B mouse gut explant contraction JPEG time-lapse images/lobsterClaw557.jpg]

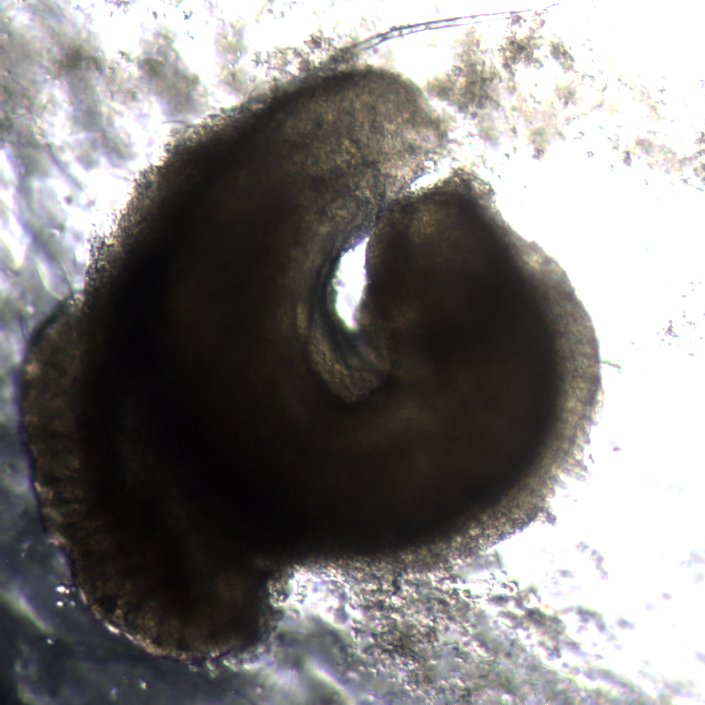

Supplement: Supplementary file 1 [file cells-11-00967-s001.zip › supplemetal videos/figure 3B mouse gut explant contraction JPEG time-lapse images/lobsterClaw027.jpg]

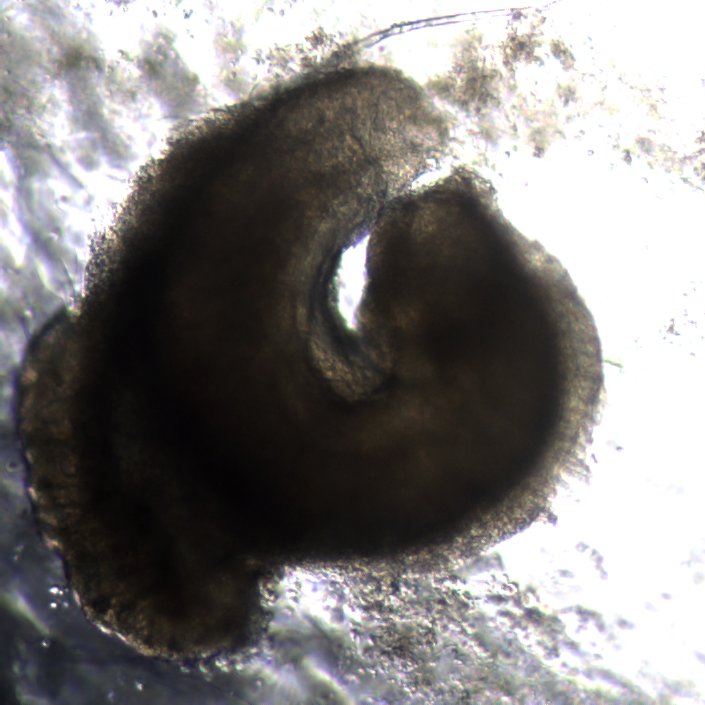

Supplement: Supplementary file 1 [file cells-11-00967-s001.zip › supplemetal videos/figure 3B mouse gut explant contraction JPEG time-lapse images/lobsterClaw033.jpg]

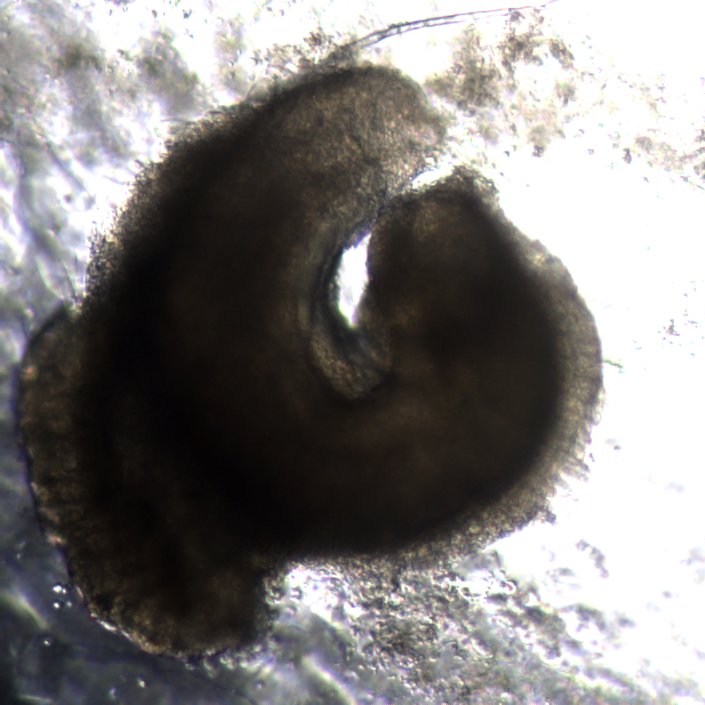

Supplement: Supplementary file 1 [file cells-11-00967-s001.zip › supplemetal videos/figure 3B mouse gut explant contraction JPEG time-lapse images/lobsterClaw190.jpg]

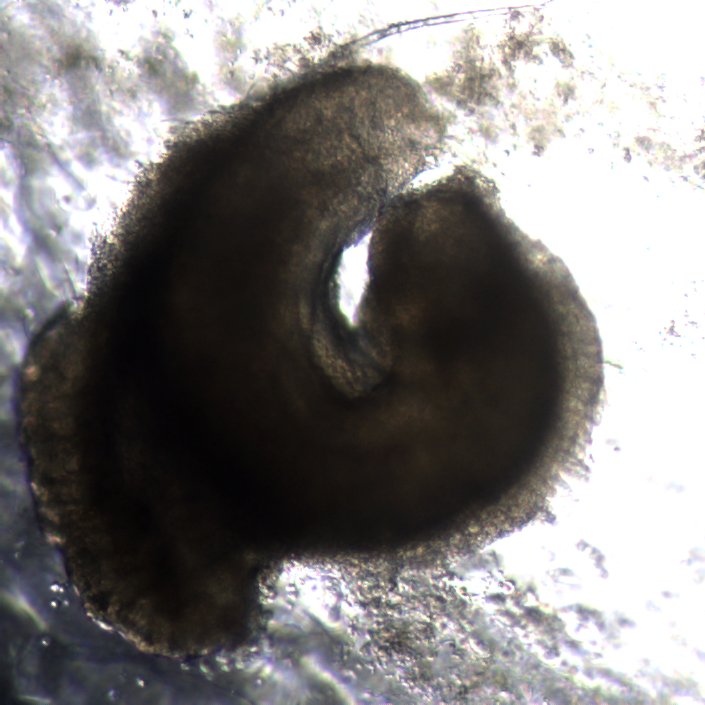

Supplement: Supplementary file 1 [file cells-11-00967-s001.zip › supplemetal videos/figure 3B mouse gut explant contraction JPEG time-lapse images/lobsterClaw184.jpg]

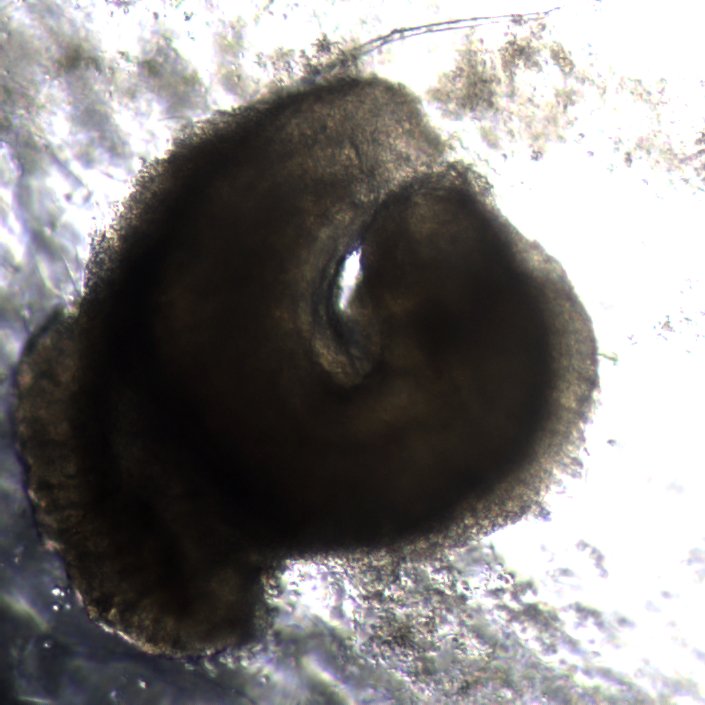

Supplement: Supplementary file 1 [file cells-11-00967-s001.zip › supplemetal videos/figure 3B mouse gut explant contraction JPEG time-lapse images/lobsterClaw153.jpg]
