# Supplementary figures and images for: 3D In Vitro Platform for Cell and Explant Culture in Liquid-like Solids (part 1 of 2)
Source: Cells. 2022 Mar 11;11(6):967. doi: 10.3390/cells11060967 (PMC8946834; doi:10.3390/cells11060967)

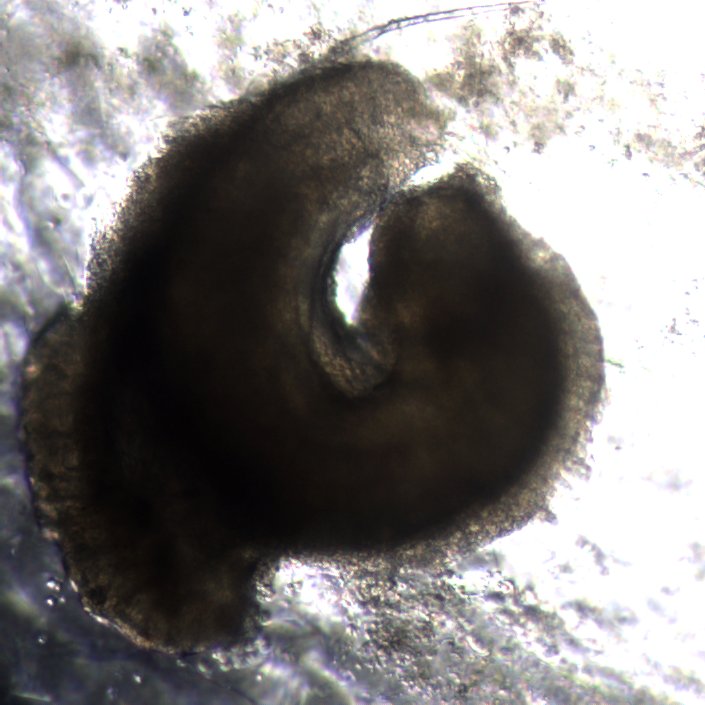

Supplement: Supplementary file 1 [file cells-11-00967-s001.zip › supplemetal videos/figure 3B mouse gut explant contraction JPEG time-lapse images/lobsterClaw441.jpg]

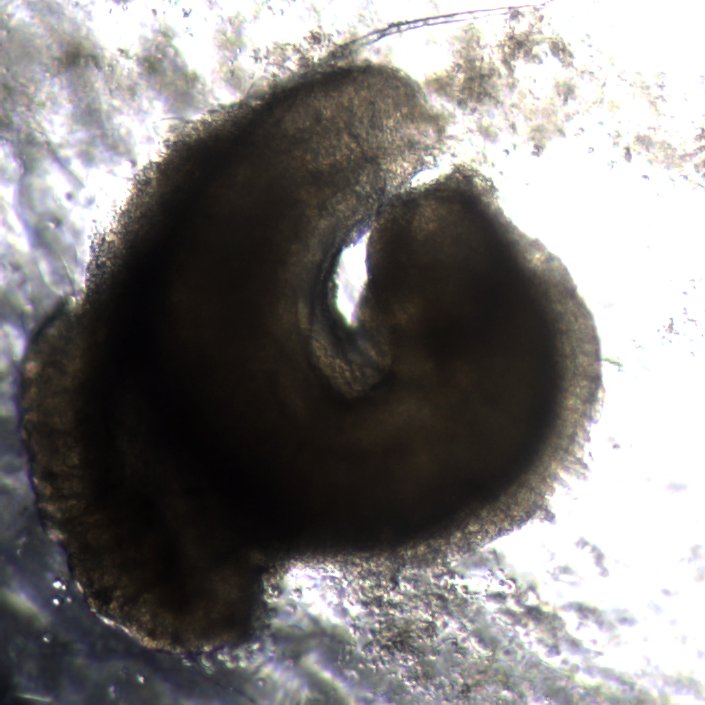

Supplement: Supplementary file 1 [file cells-11-00967-s001.zip › supplemetal videos/figure 3B mouse gut explant contraction JPEG time-lapse images/lobsterClaw327.jpg]

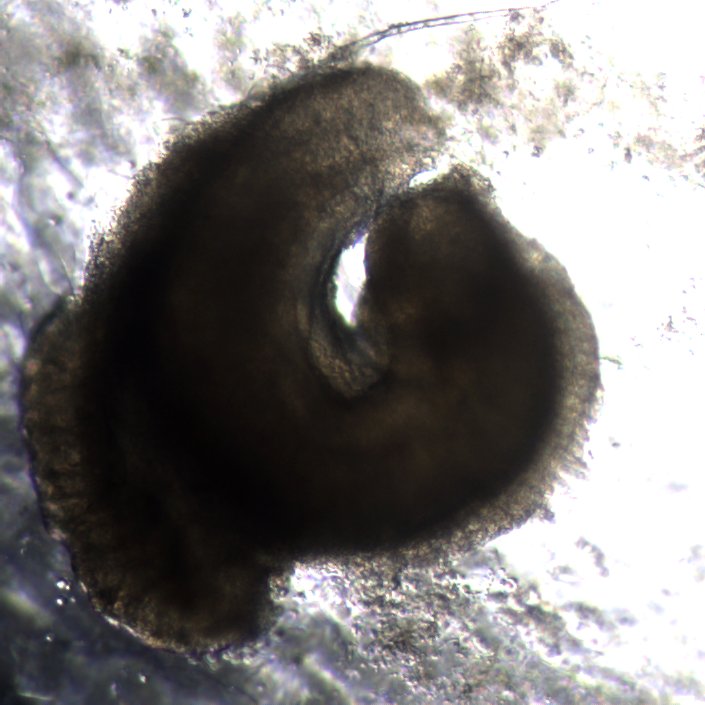

Supplement: Supplementary file 1 [file cells-11-00967-s001.zip › supplemetal videos/figure 3B mouse gut explant contraction JPEG time-lapse images/lobsterClaw333.jpg]

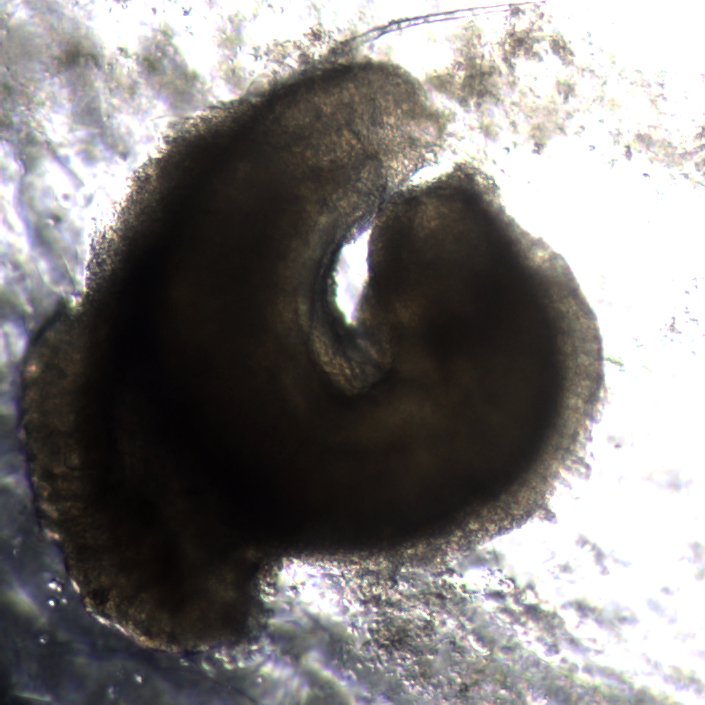

Supplement: Supplementary file 1 [file cells-11-00967-s001.zip › supplemetal videos/figure 3B mouse gut explant contraction JPEG time-lapse images/lobsterClaw455.jpg]

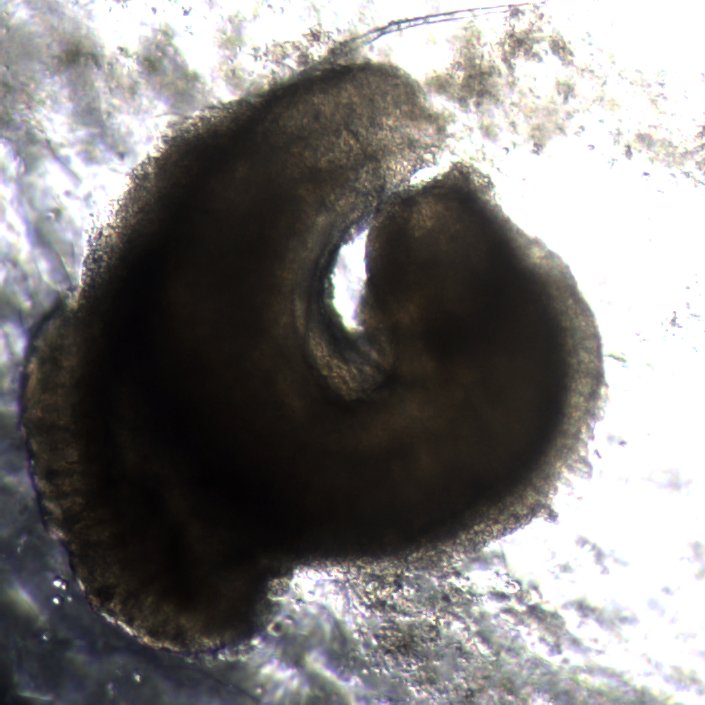

Supplement: Supplementary file 1 [file cells-11-00967-s001.zip › supplemetal videos/figure 3B mouse gut explant contraction JPEG time-lapse images/lobsterClaw469.jpg]

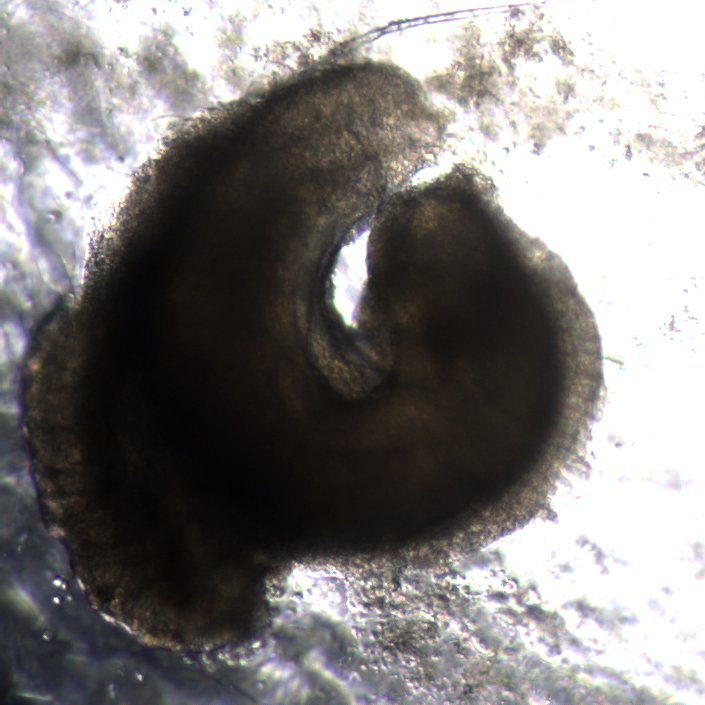

Supplement: Supplementary file 1 [file cells-11-00967-s001.zip › supplemetal videos/figure 3B mouse gut explant contraction JPEG time-lapse images/lobsterClaw482.jpg]

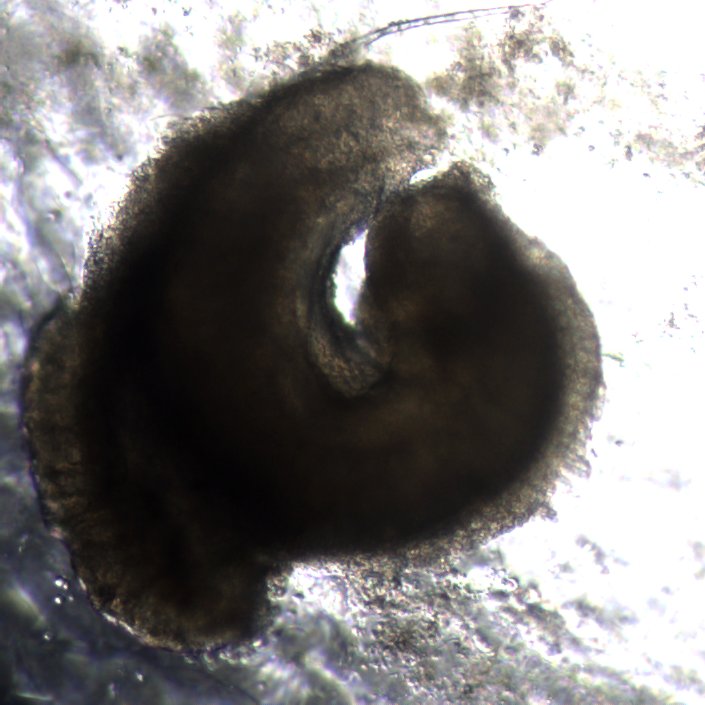

Supplement: Supplementary file 1 [file cells-11-00967-s001.zip › supplemetal videos/figure 3B mouse gut explant contraction JPEG time-lapse images/lobsterClaw496.jpg]

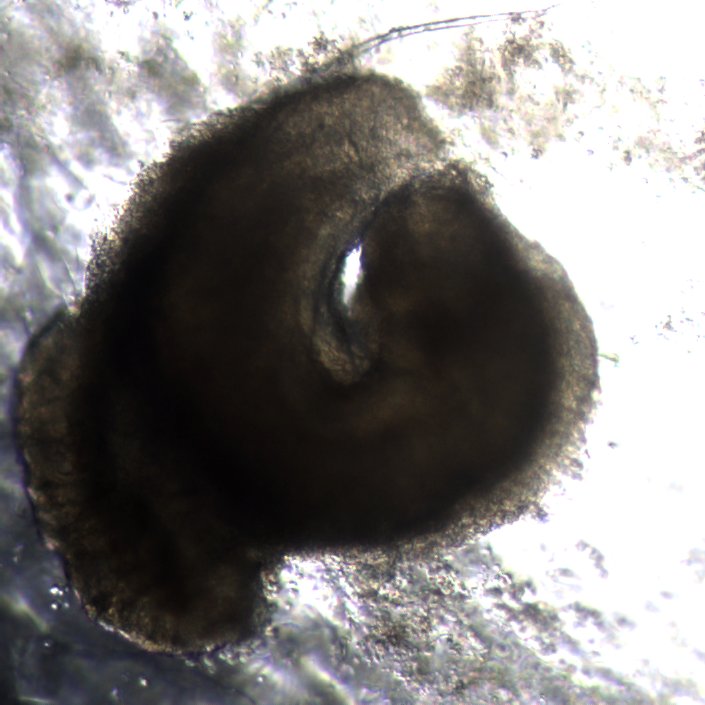

Supplement: Supplementary file 1 [file cells-11-00967-s001.zip › supplemetal videos/figure 3B mouse gut explant contraction JPEG time-lapse images/lobsterClaw125.jpg]

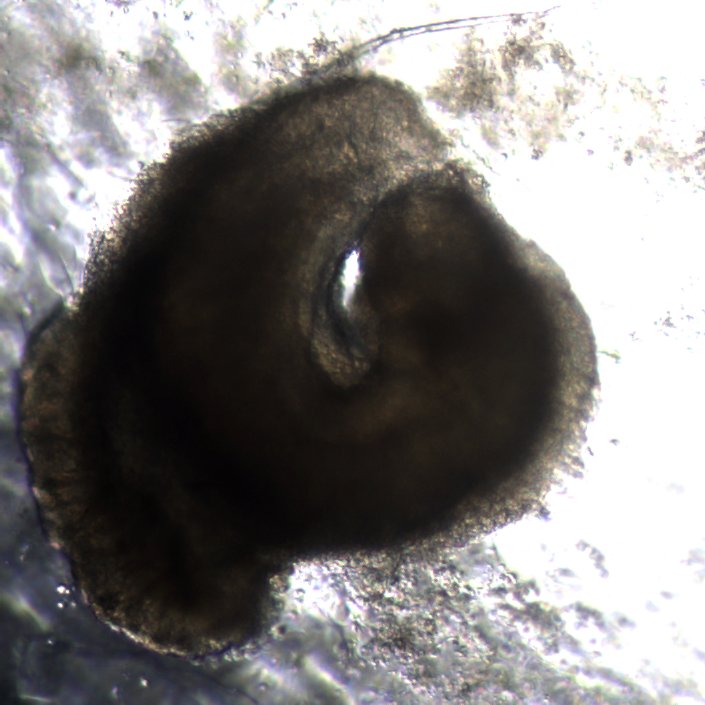

Supplement: Supplementary file 1 [file cells-11-00967-s001.zip › supplemetal videos/figure 3B mouse gut explant contraction JPEG time-lapse images/lobsterClaw131.jpg]

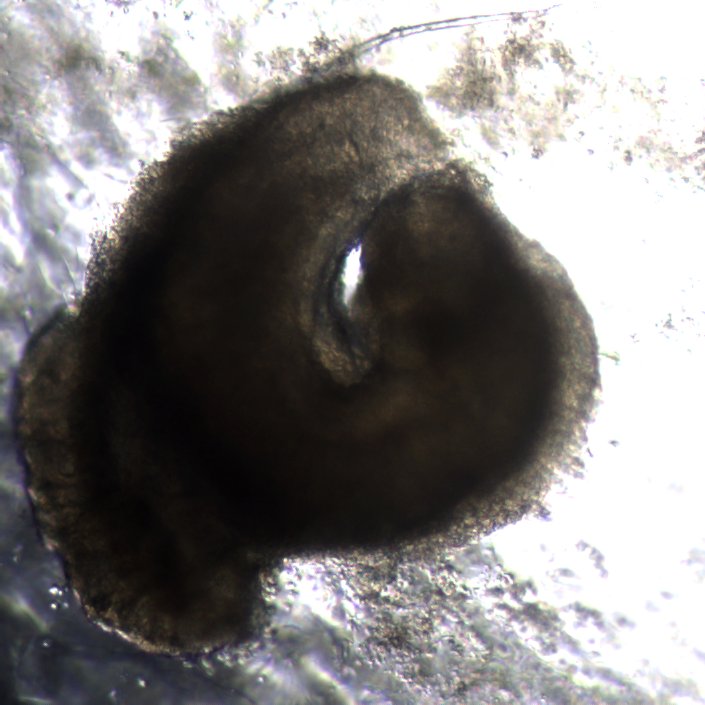

Supplement: Supplementary file 1 [file cells-11-00967-s001.zip › supplemetal videos/figure 3B mouse gut explant contraction JPEG time-lapse images/lobsterClaw119.jpg]

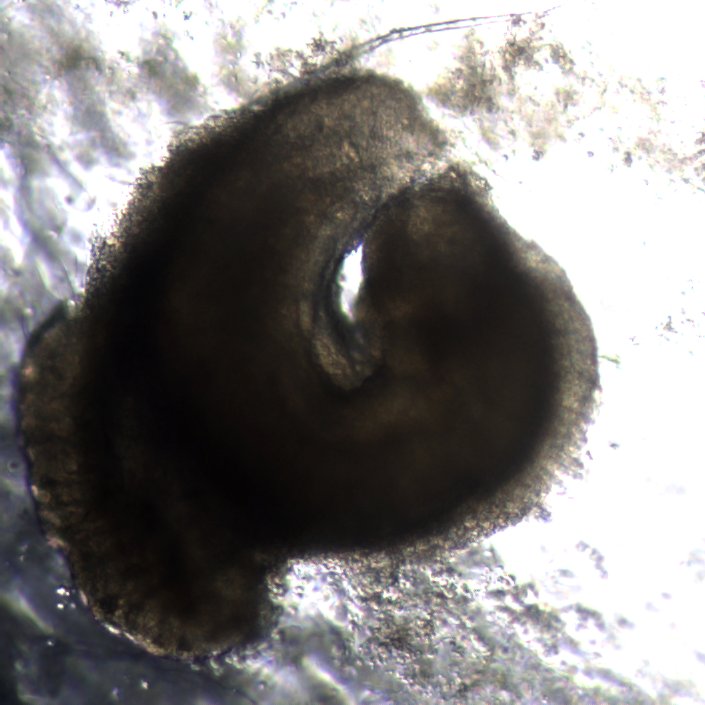

Supplement: Supplementary file 1 [file cells-11-00967-s001.zip › supplemetal videos/figure 3B mouse gut explant contraction JPEG time-lapse images/lobsterClaw051.jpg]

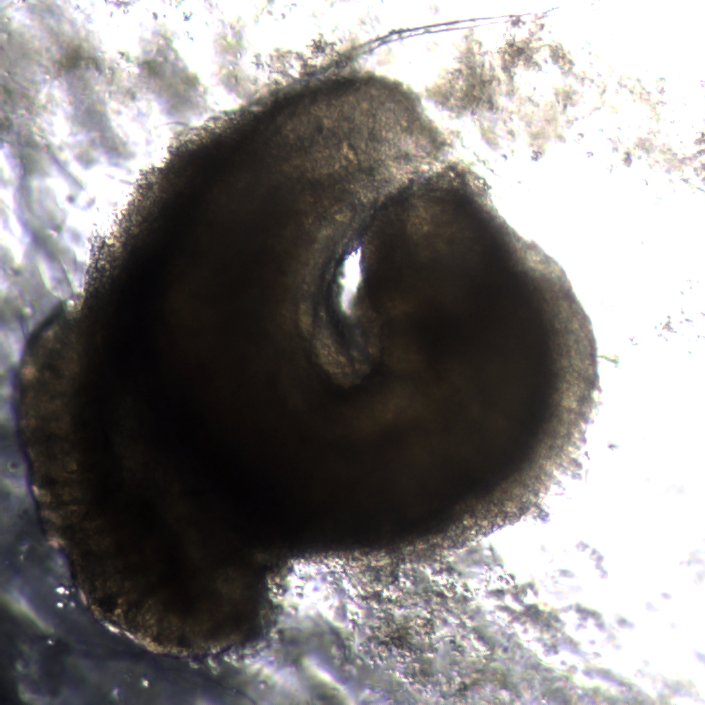

Supplement: Supplementary file 1 [file cells-11-00967-s001.zip › supplemetal videos/figure 3B mouse gut explant contraction JPEG time-lapse images/lobsterClaw045.jpg]

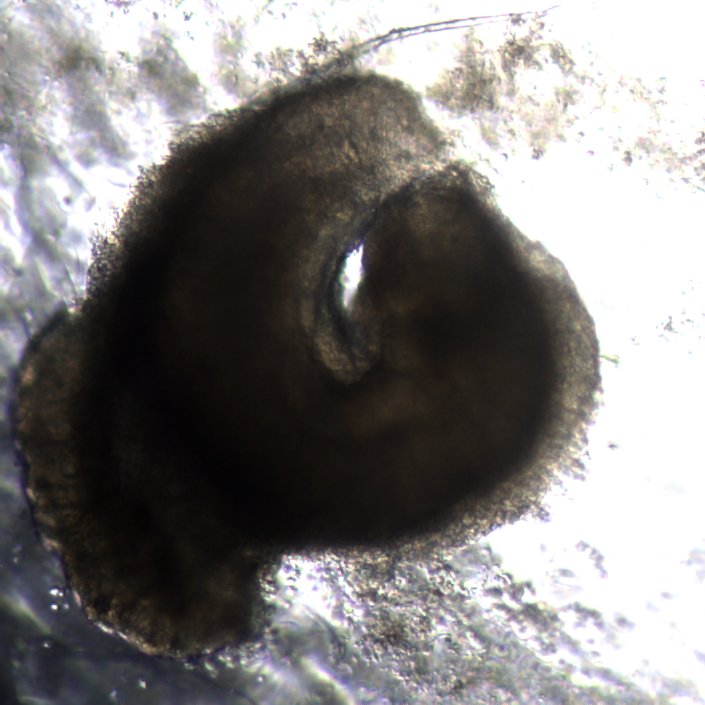

Supplement: Supplementary file 1 [file cells-11-00967-s001.zip › supplemetal videos/figure 3B mouse gut explant contraction JPEG time-lapse images/lobsterClaw079.jpg]

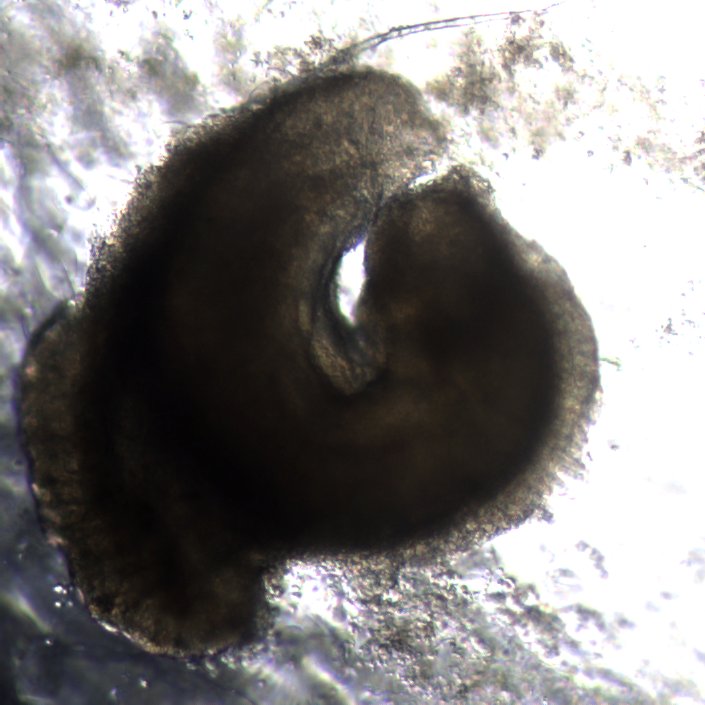

Supplement: Supplementary file 1 [file cells-11-00967-s001.zip › supplemetal videos/figure 3B mouse gut explant contraction JPEG time-lapse images/lobsterClaw092.jpg]

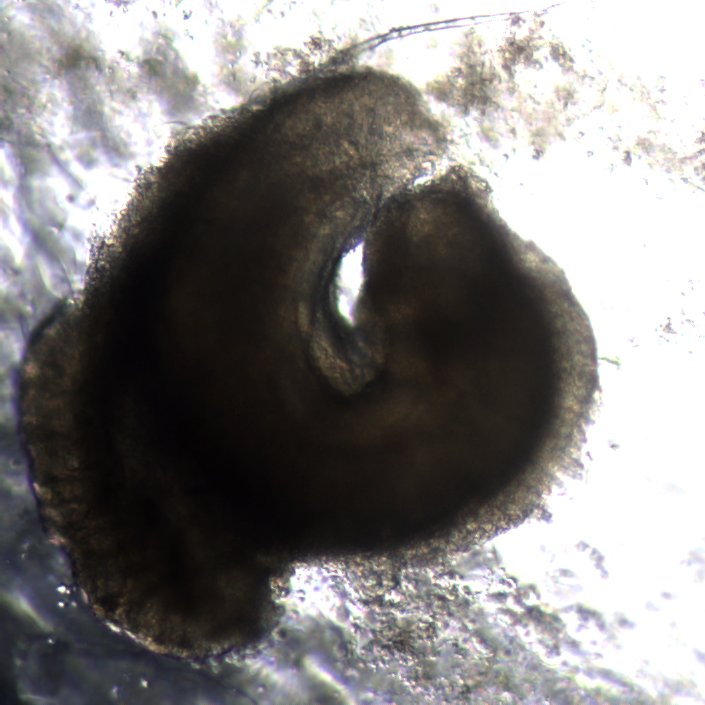

Supplement: Supplementary file 1 [file cells-11-00967-s001.zip › supplemetal videos/figure 3B mouse gut explant contraction JPEG time-lapse images/lobsterClaw086.jpg]

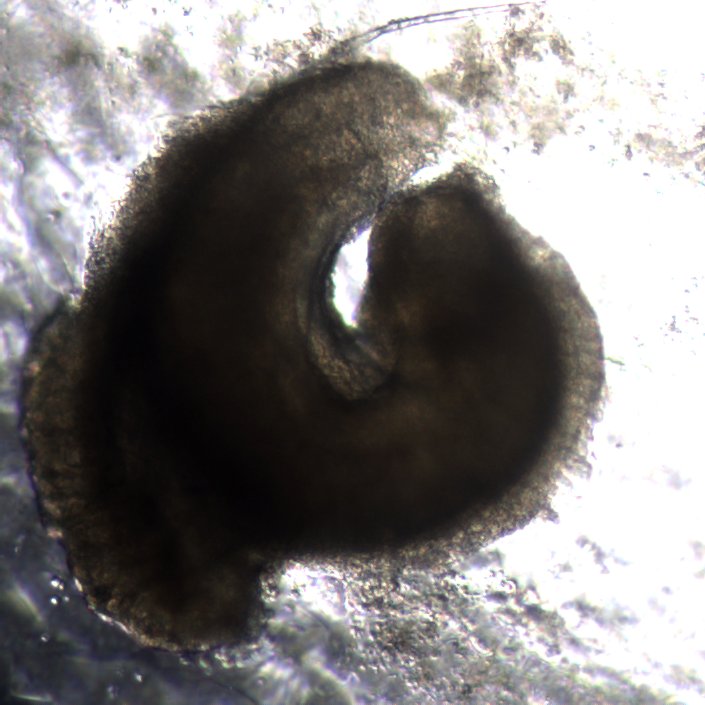

Supplement: Supplementary file 1 [file cells-11-00967-s001.zip › supplemetal videos/figure 3B mouse gut explant contraction JPEG time-lapse images/lobsterClaw535.jpg]

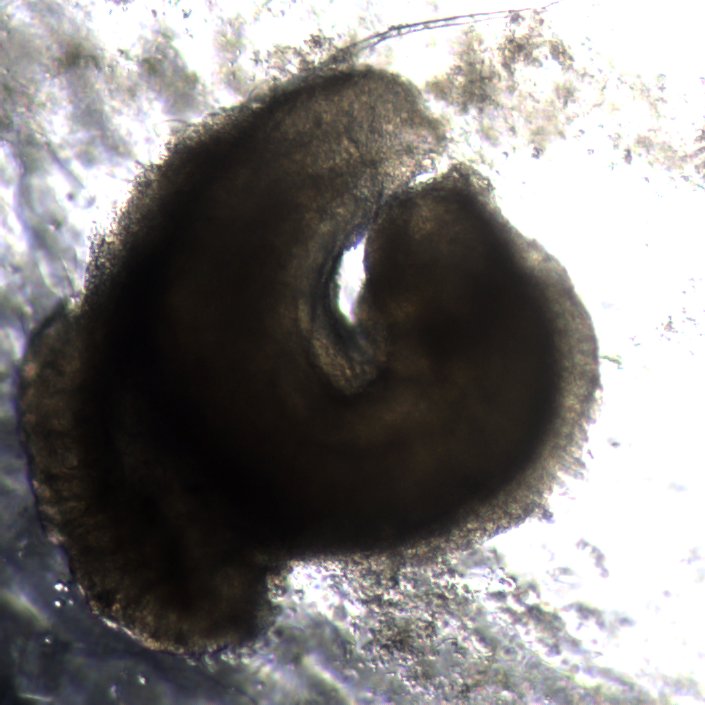

Supplement: Supplementary file 1 [file cells-11-00967-s001.zip › supplemetal videos/figure 3B mouse gut explant contraction JPEG time-lapse images/lobsterClaw253.jpg]

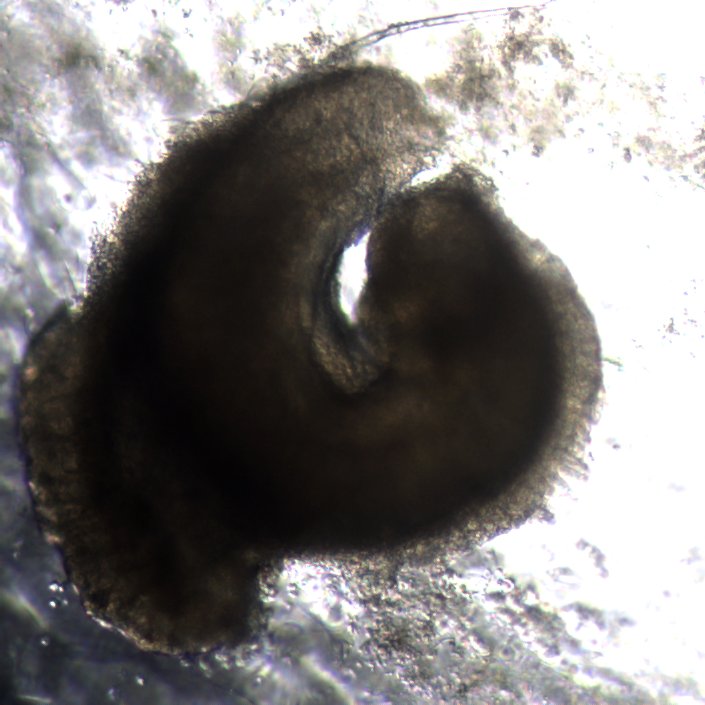

Supplement: Supplementary file 1 [file cells-11-00967-s001.zip › supplemetal videos/figure 3B mouse gut explant contraction JPEG time-lapse images/lobsterClaw247.jpg]

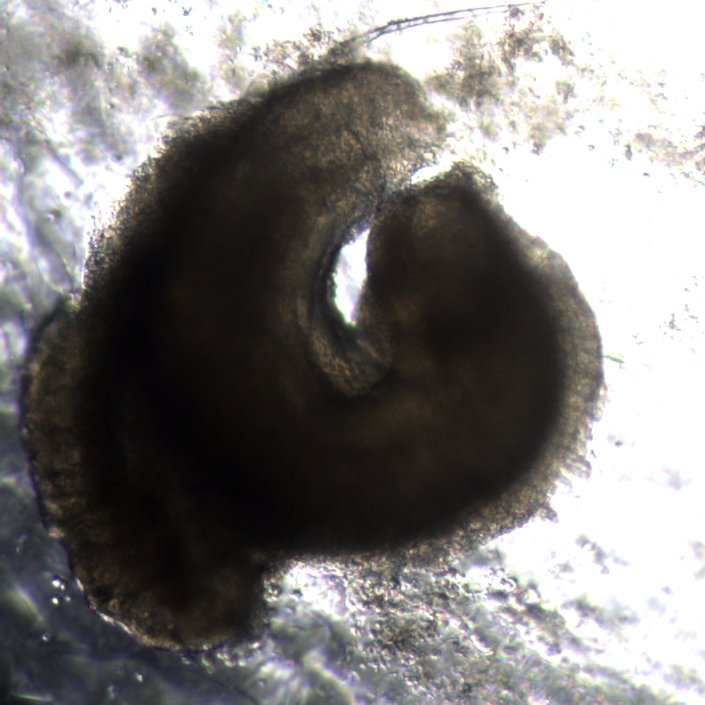

Supplement: Supplementary file 1 [file cells-11-00967-s001.zip › supplemetal videos/figure 3B mouse gut explant contraction JPEG time-lapse images/lobsterClaw521.jpg]

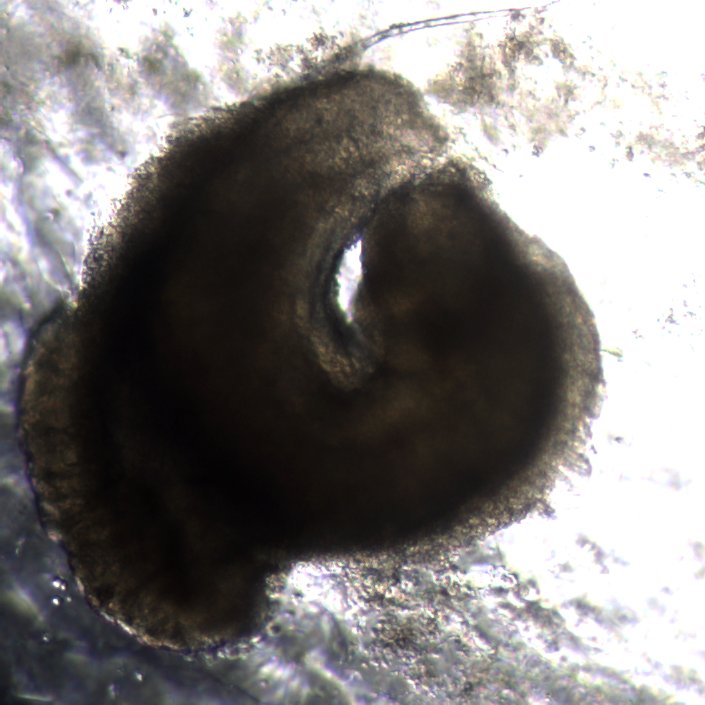

Supplement: Supplementary file 1 [file cells-11-00967-s001.zip › supplemetal videos/figure 3B mouse gut explant contraction JPEG time-lapse images/lobsterClaw509.jpg]

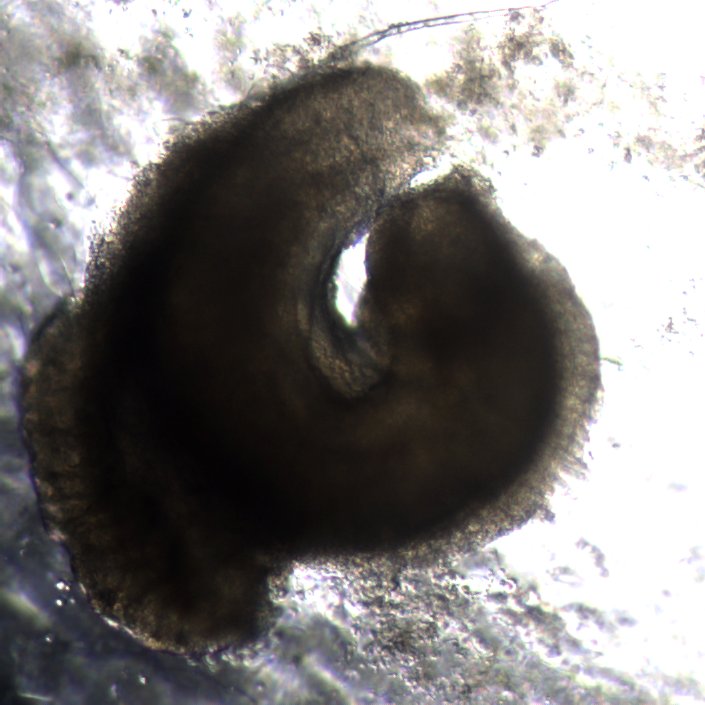

Supplement: Supplementary file 1 [file cells-11-00967-s001.zip › supplemetal videos/figure 3B mouse gut explant contraction JPEG time-lapse images/lobsterClaw290.jpg]

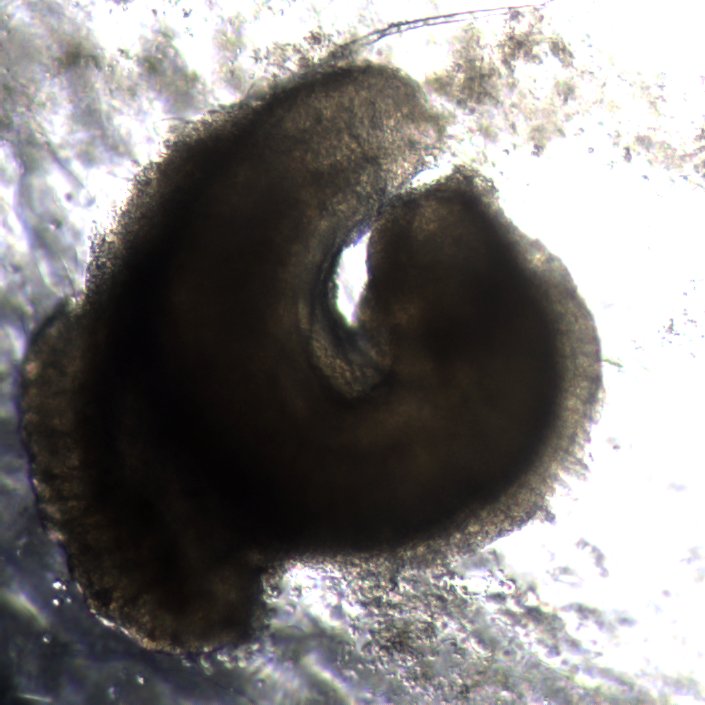

Supplement: Supplementary file 1 [file cells-11-00967-s001.zip › supplemetal videos/figure 3B mouse gut explant contraction JPEG time-lapse images/lobsterClaw284.jpg]

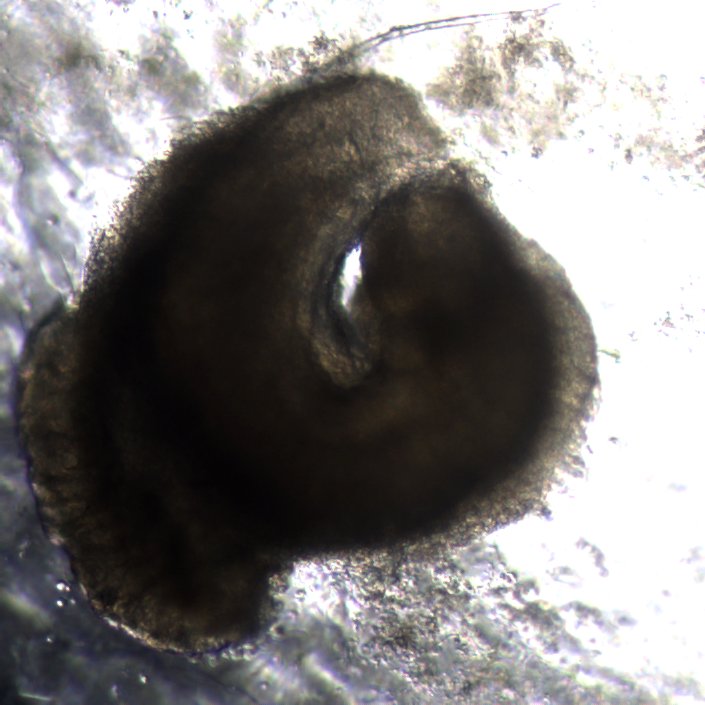

Supplement: Supplementary file 1 [file cells-11-00967-s001.zip › supplemetal videos/figure 3B mouse gut explant contraction JPEG time-lapse images/lobsterClaw285.jpg]

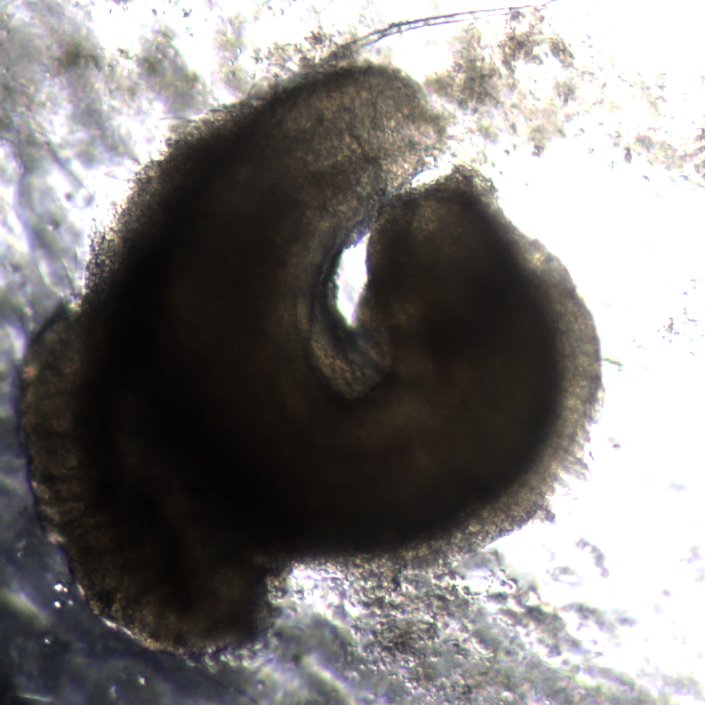

Supplement: Supplementary file 1 [file cells-11-00967-s001.zip › supplemetal videos/figure 3B mouse gut explant contraction JPEG time-lapse images/lobsterClaw291.jpg]

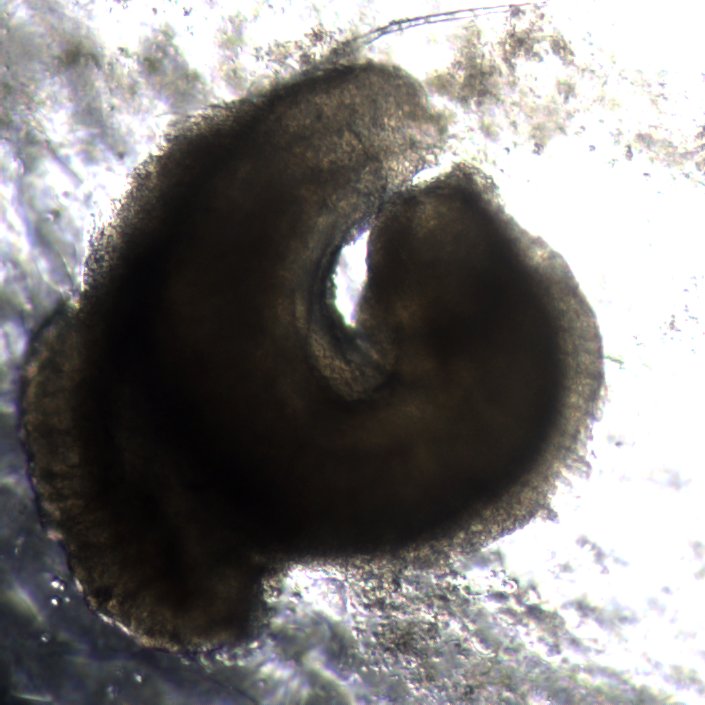

Supplement: Supplementary file 1 [file cells-11-00967-s001.zip › supplemetal videos/figure 3B mouse gut explant contraction JPEG time-lapse images/lobsterClaw508.jpg]

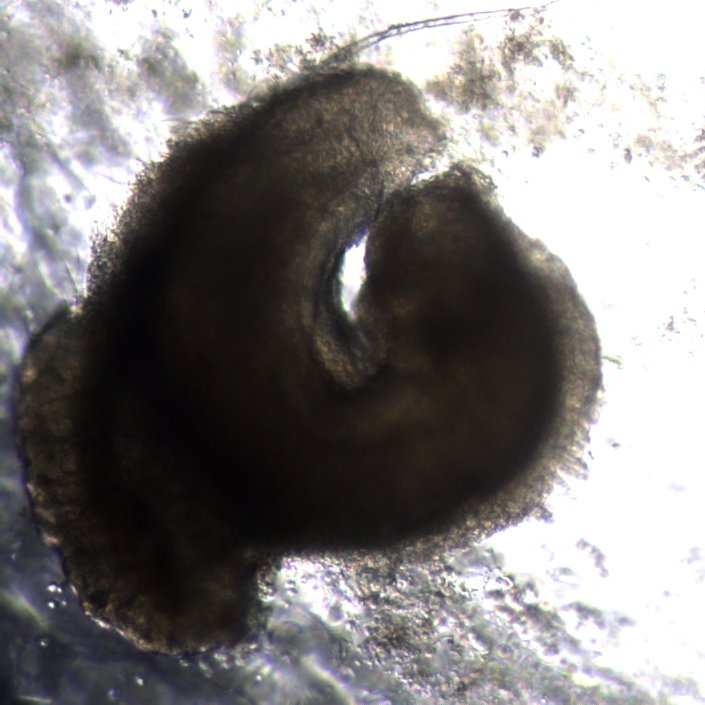

Supplement: Supplementary file 1 [file cells-11-00967-s001.zip › supplemetal videos/figure 3B mouse gut explant contraction JPEG time-lapse images/lobsterClaw246.jpg]

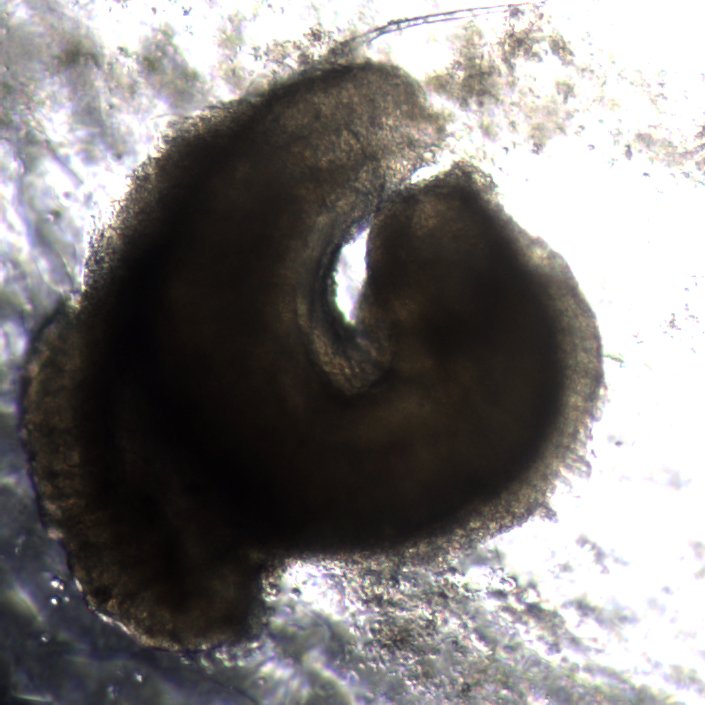

Supplement: Supplementary file 1 [file cells-11-00967-s001.zip › supplemetal videos/figure 3B mouse gut explant contraction JPEG time-lapse images/lobsterClaw520.jpg]

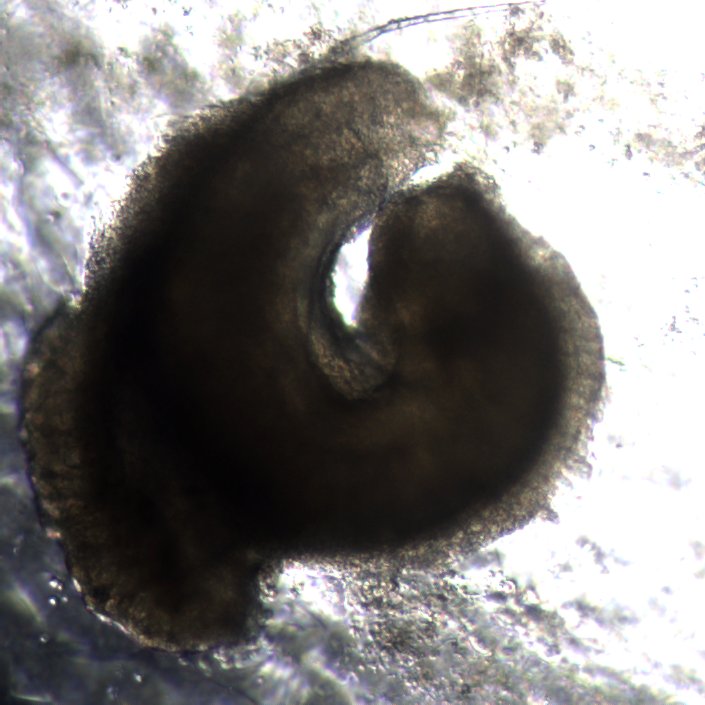

Supplement: Supplementary file 1 [file cells-11-00967-s001.zip › supplemetal videos/figure 3B mouse gut explant contraction JPEG time-lapse images/lobsterClaw534.jpg]

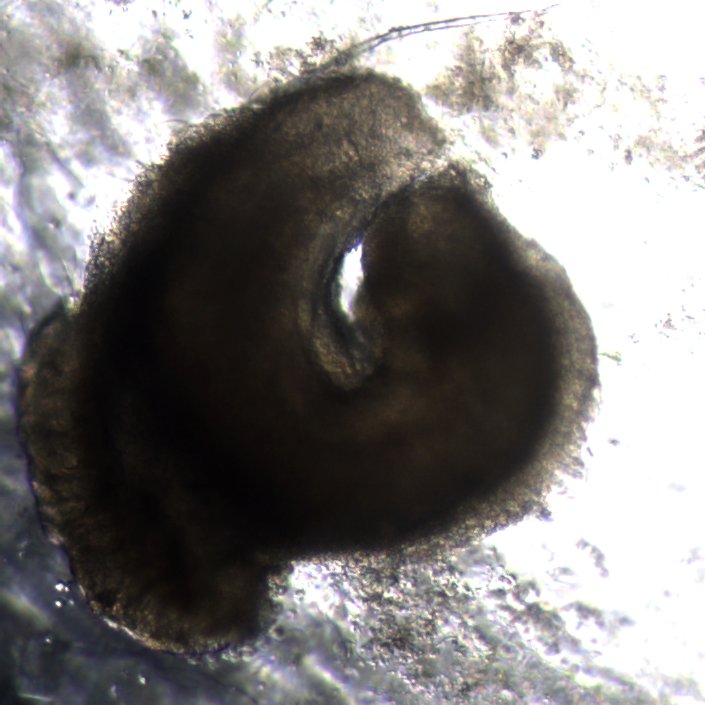

Supplement: Supplementary file 1 [file cells-11-00967-s001.zip › supplemetal videos/figure 3B mouse gut explant contraction JPEG time-lapse images/lobsterClaw252.jpg]

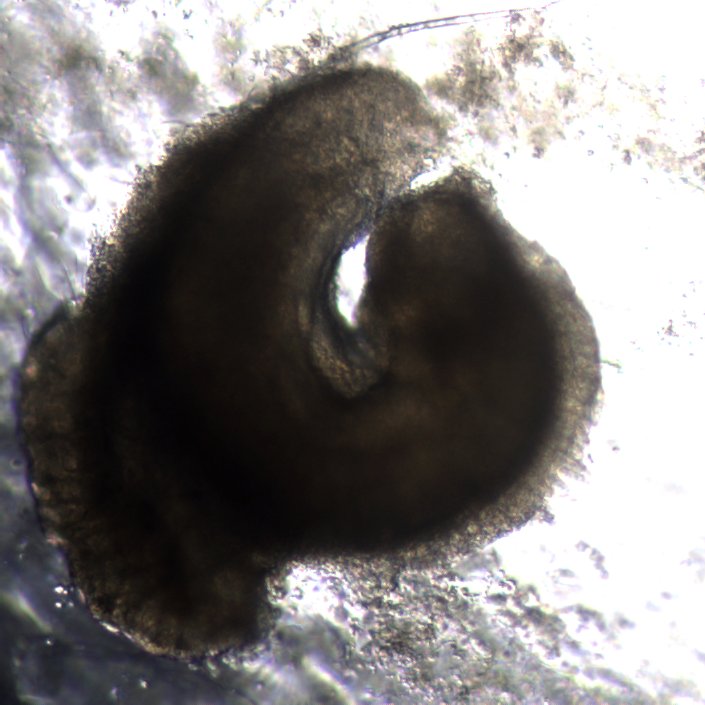

Supplement: Supplementary file 1 [file cells-11-00967-s001.zip › supplemetal videos/figure 3B mouse gut explant contraction JPEG time-lapse images/lobsterClaw087.jpg]

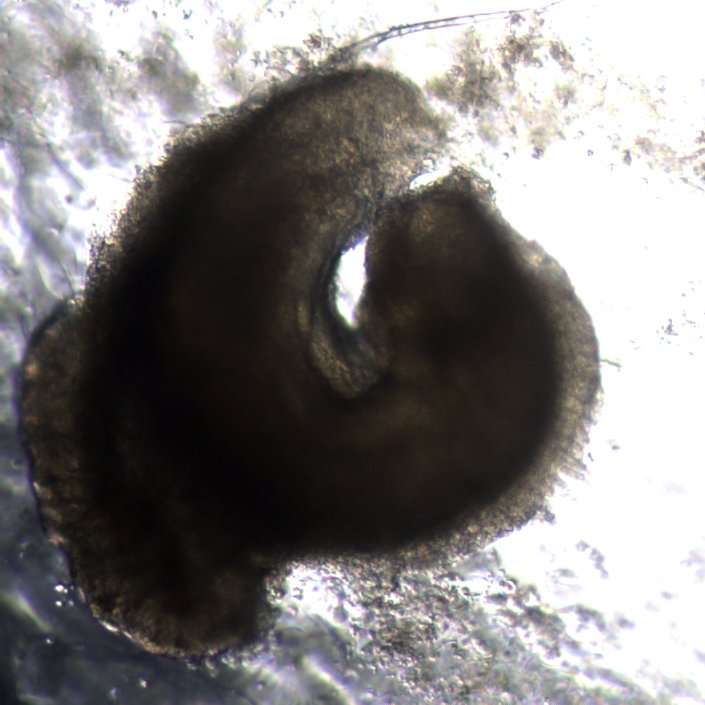

Supplement: Supplementary file 1 [file cells-11-00967-s001.zip › supplemetal videos/figure 3B mouse gut explant contraction JPEG time-lapse images/lobsterClaw093.jpg]

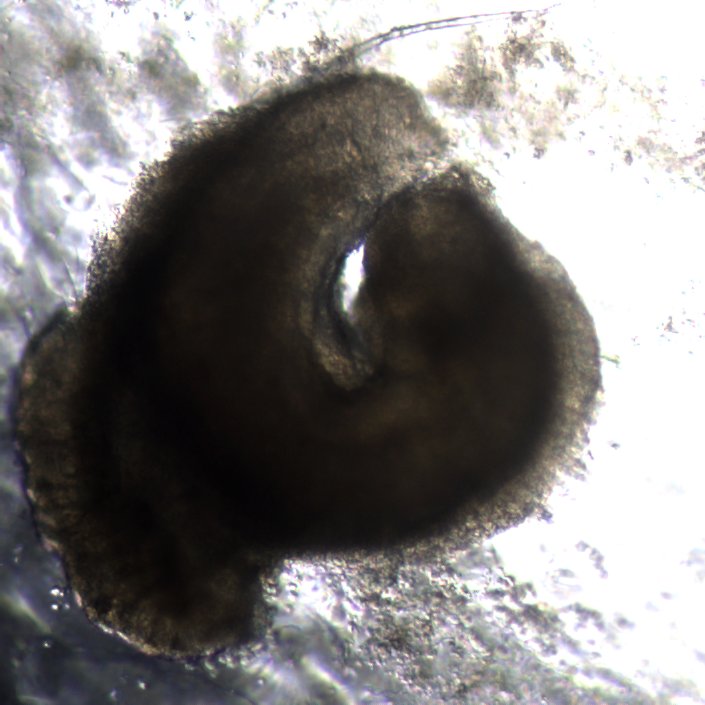

Supplement: Supplementary file 1 [file cells-11-00967-s001.zip › supplemetal videos/figure 3B mouse gut explant contraction JPEG time-lapse images/lobsterClaw078.jpg]

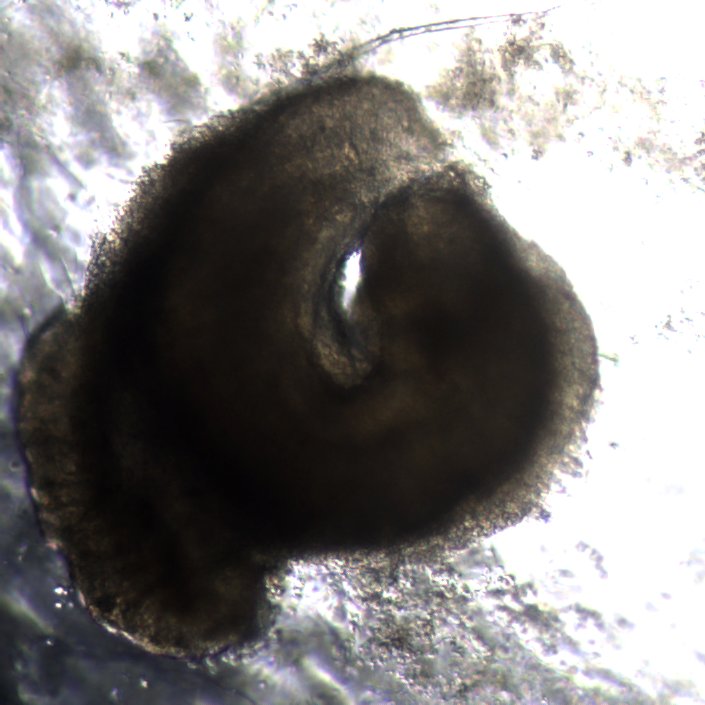

Supplement: Supplementary file 1 [file cells-11-00967-s001.zip › supplemetal videos/figure 3B mouse gut explant contraction JPEG time-lapse images/lobsterClaw044.jpg]

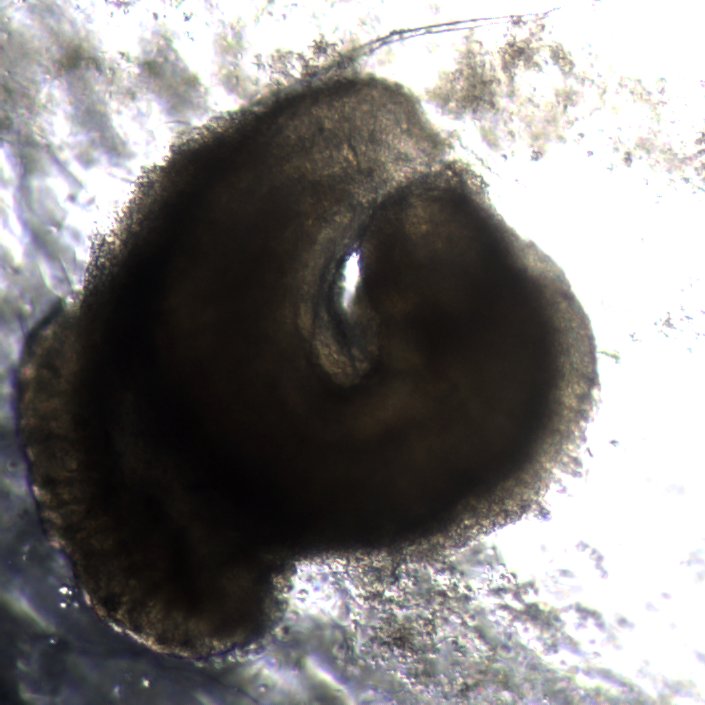

Supplement: Supplementary file 1 [file cells-11-00967-s001.zip › supplemetal videos/figure 3B mouse gut explant contraction JPEG time-lapse images/lobsterClaw050.jpg]

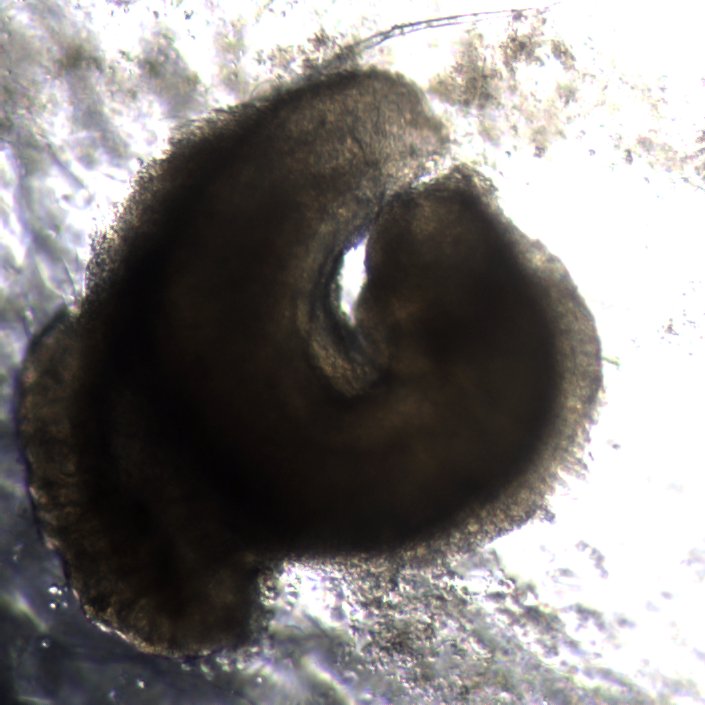

Supplement: Supplementary file 1 [file cells-11-00967-s001.zip › supplemetal videos/figure 3B mouse gut explant contraction JPEG time-lapse images/lobsterClaw118.jpg]

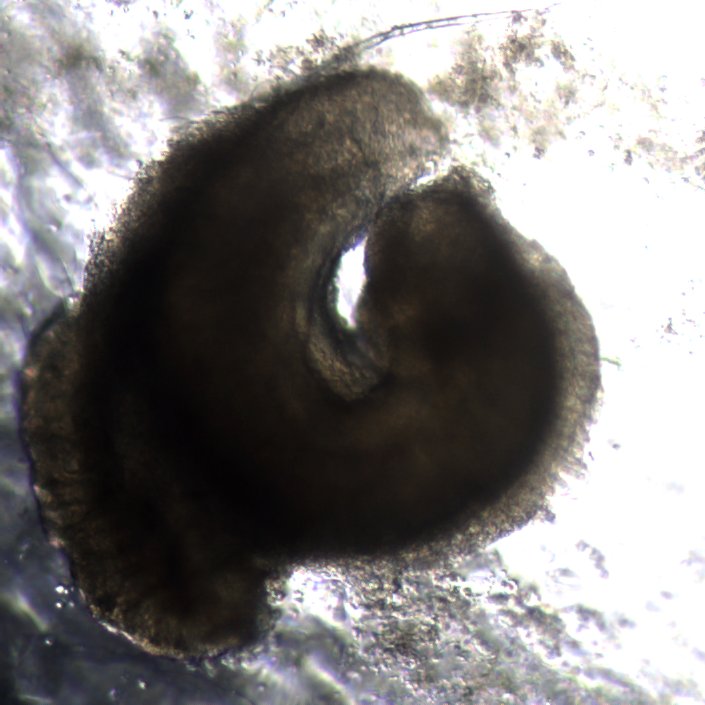

Supplement: Supplementary file 1 [file cells-11-00967-s001.zip › supplemetal videos/figure 3B mouse gut explant contraction JPEG time-lapse images/lobsterClaw130.jpg]

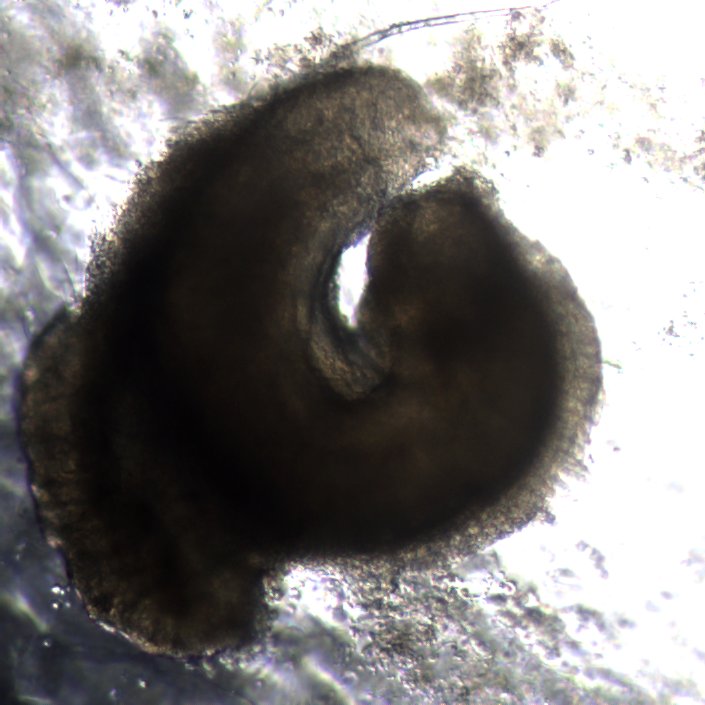

Supplement: Supplementary file 1 [file cells-11-00967-s001.zip › supplemetal videos/figure 3B mouse gut explant contraction JPEG time-lapse images/lobsterClaw124.jpg]

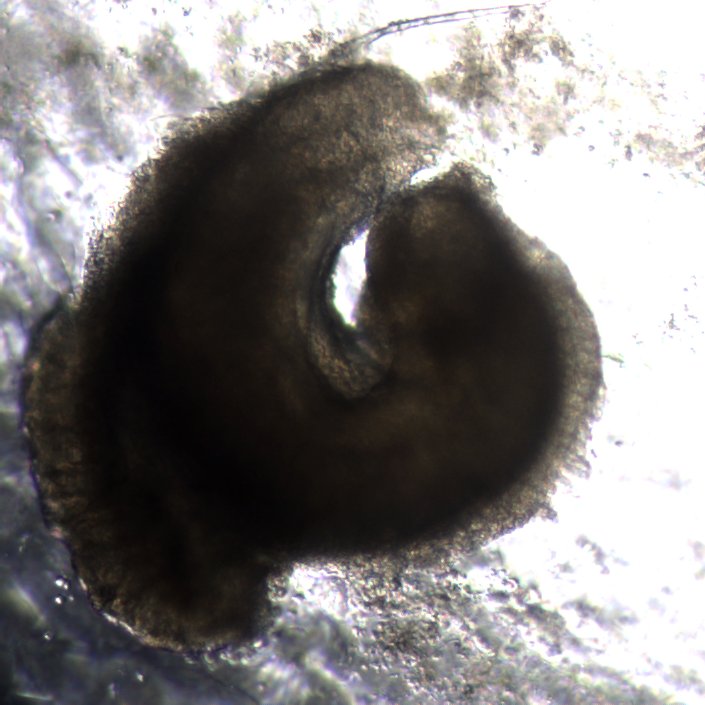

Supplement: Supplementary file 1 [file cells-11-00967-s001.zip › supplemetal videos/figure 3B mouse gut explant contraction JPEG time-lapse images/lobsterClaw497.jpg]

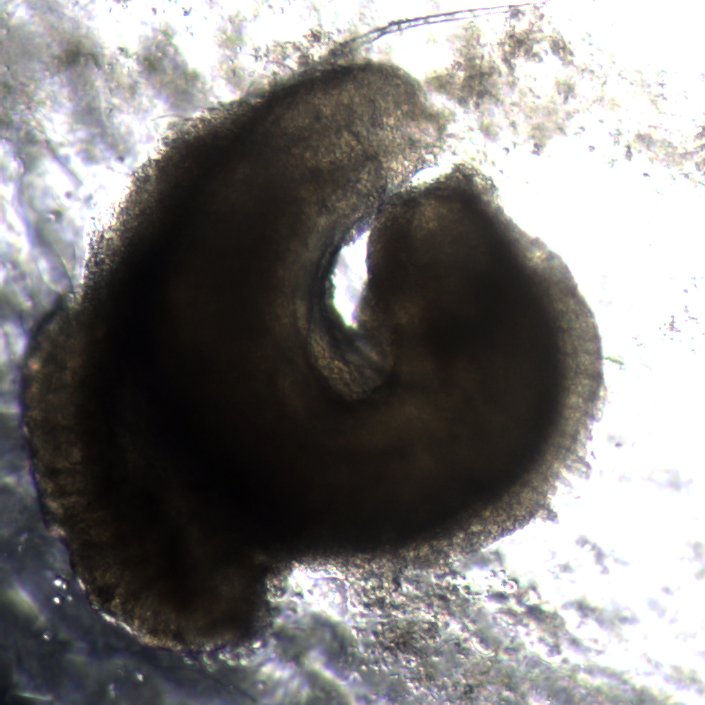

Supplement: Supplementary file 1 [file cells-11-00967-s001.zip › supplemetal videos/figure 3B mouse gut explant contraction JPEG time-lapse images/lobsterClaw483.jpg]

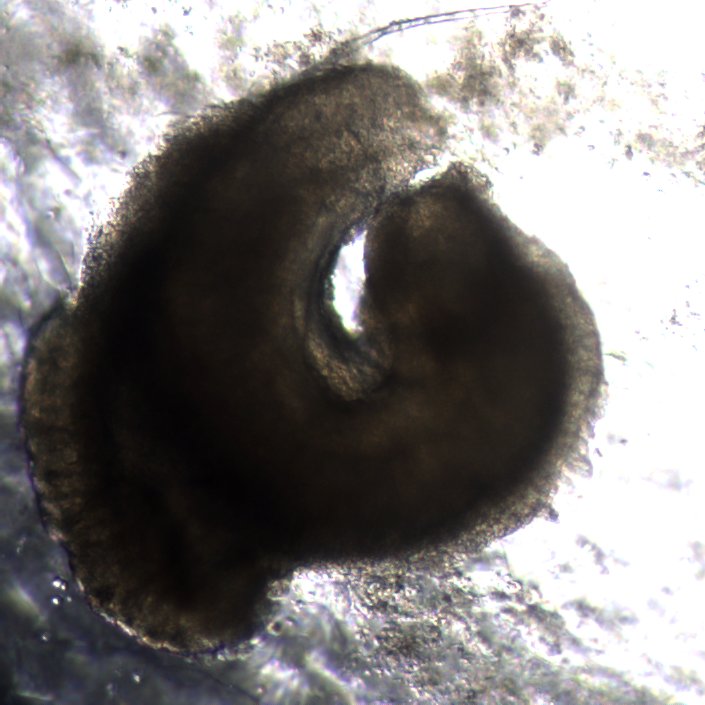

Supplement: Supplementary file 1 [file cells-11-00967-s001.zip › supplemetal videos/figure 3B mouse gut explant contraction JPEG time-lapse images/lobsterClaw468.jpg]

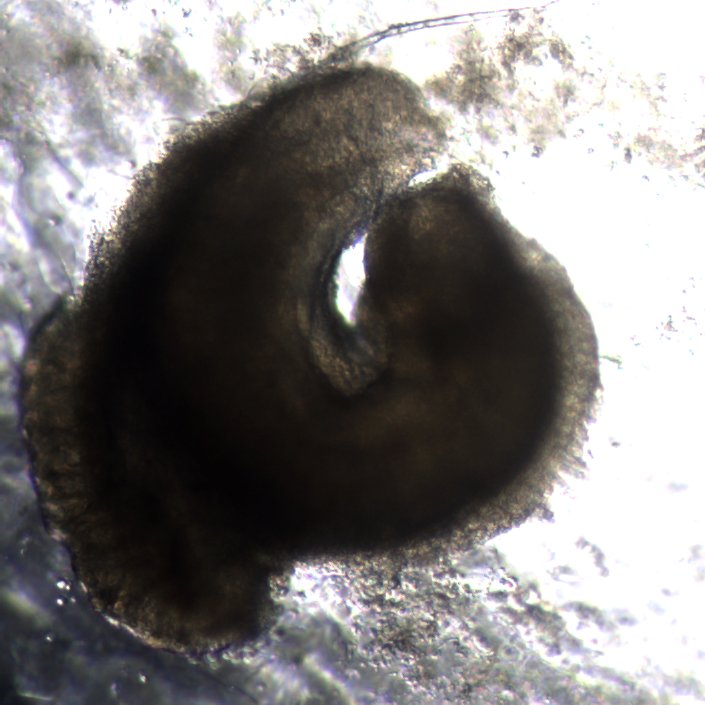

Supplement: Supplementary file 1 [file cells-11-00967-s001.zip › supplemetal videos/figure 3B mouse gut explant contraction JPEG time-lapse images/lobsterClaw332.jpg]

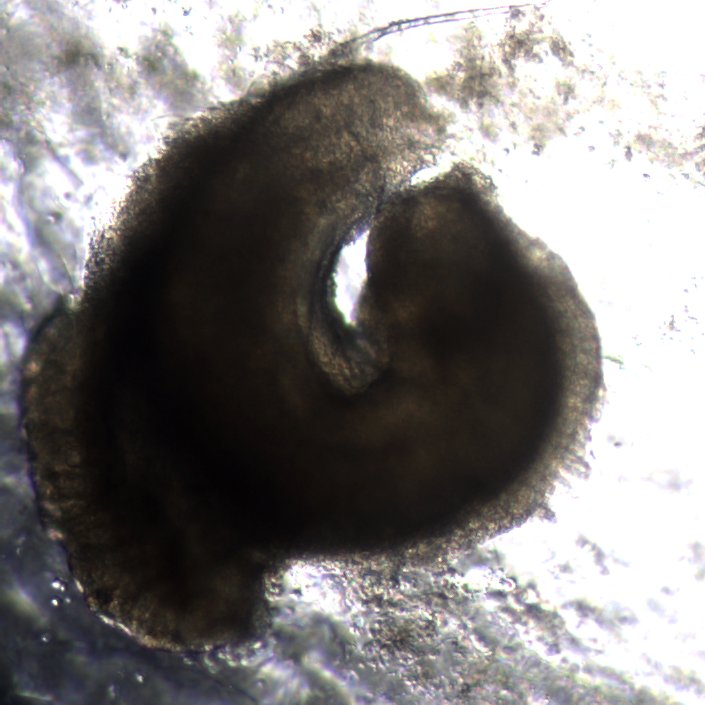

Supplement: Supplementary file 1 [file cells-11-00967-s001.zip › supplemetal videos/figure 3B mouse gut explant contraction JPEG time-lapse images/lobsterClaw454.jpg]

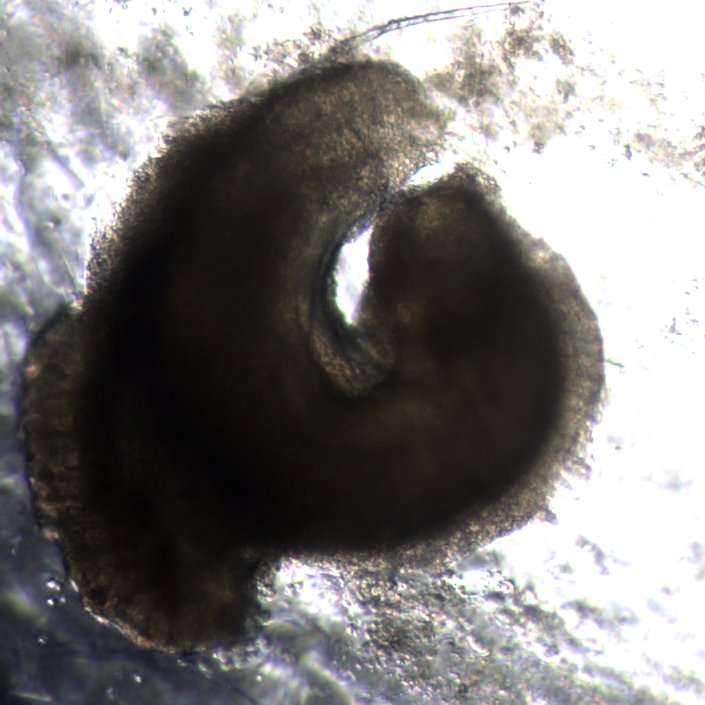

Supplement: Supplementary file 1 [file cells-11-00967-s001.zip › supplemetal videos/figure 3B mouse gut explant contraction JPEG time-lapse images/lobsterClaw440.jpg]

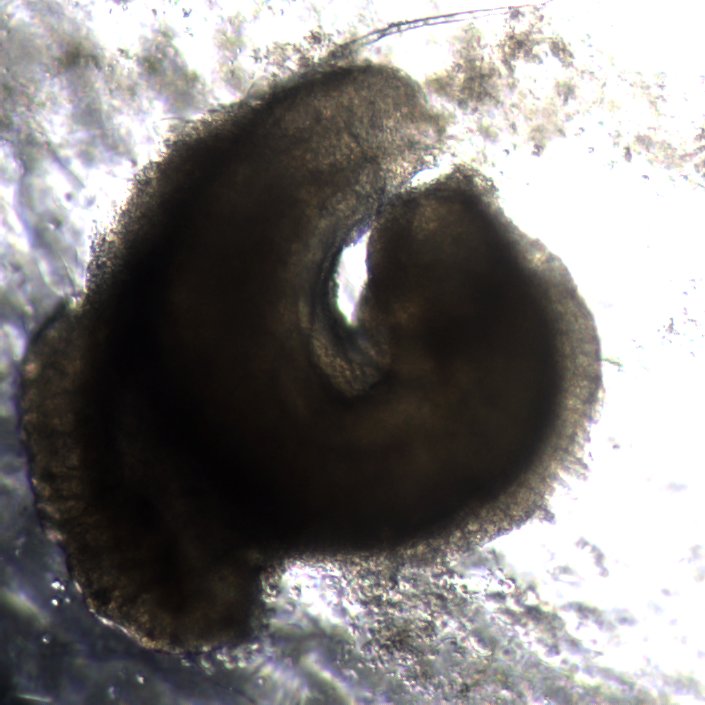

Supplement: Supplementary file 1 [file cells-11-00967-s001.zip › supplemetal videos/figure 3B mouse gut explant contraction JPEG time-lapse images/lobsterClaw326.jpg]

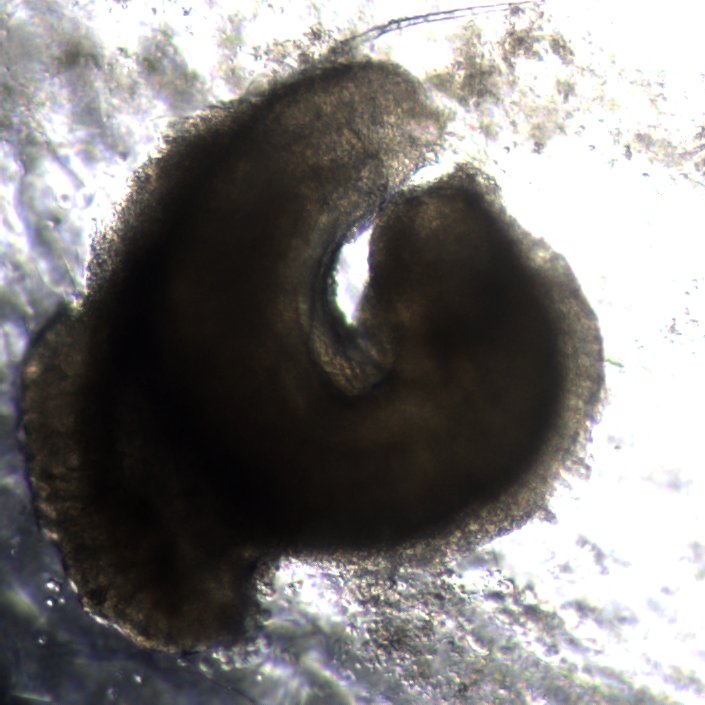

Supplement: Supplementary file 1 [file cells-11-00967-s001.zip › supplemetal videos/figure 3B mouse gut explant contraction JPEG time-lapse images/lobsterClaw456.jpg]

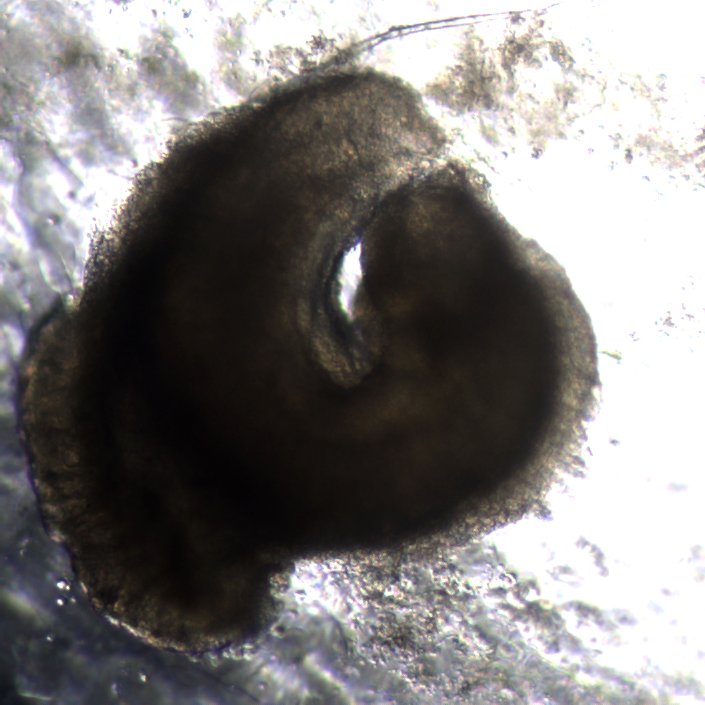

Supplement: Supplementary file 1 [file cells-11-00967-s001.zip › supplemetal videos/figure 3B mouse gut explant contraction JPEG time-lapse images/lobsterClaw330.jpg]

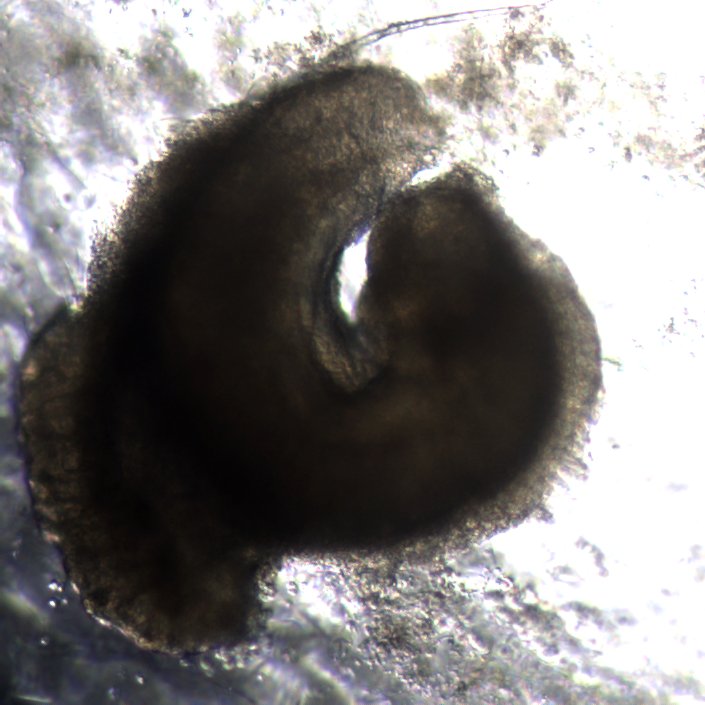

Supplement: Supplementary file 1 [file cells-11-00967-s001.zip › supplemetal videos/figure 3B mouse gut explant contraction JPEG time-lapse images/lobsterClaw324.jpg]

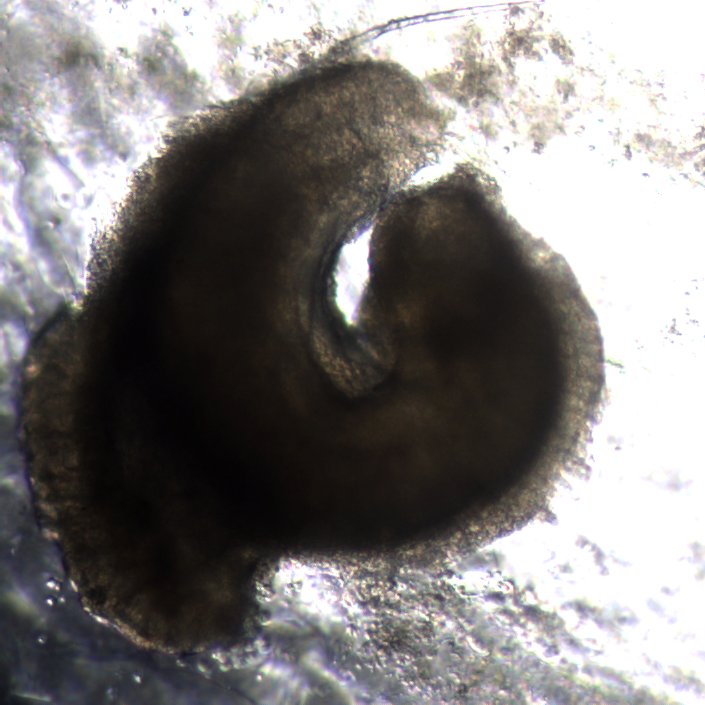

Supplement: Supplementary file 1 [file cells-11-00967-s001.zip › supplemetal videos/figure 3B mouse gut explant contraction JPEG time-lapse images/lobsterClaw442.jpg]

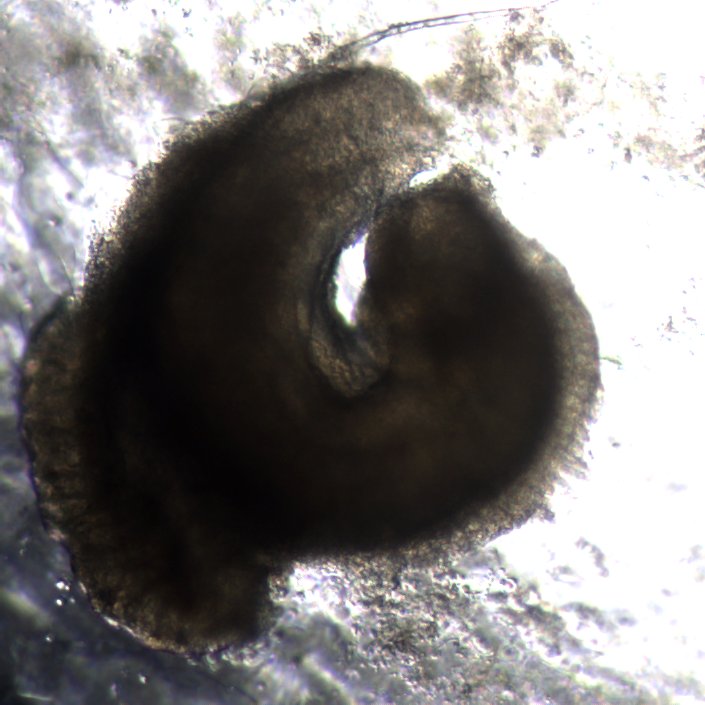

Supplement: Supplementary file 1 [file cells-11-00967-s001.zip › supplemetal videos/figure 3B mouse gut explant contraction JPEG time-lapse images/lobsterClaw318.jpg]

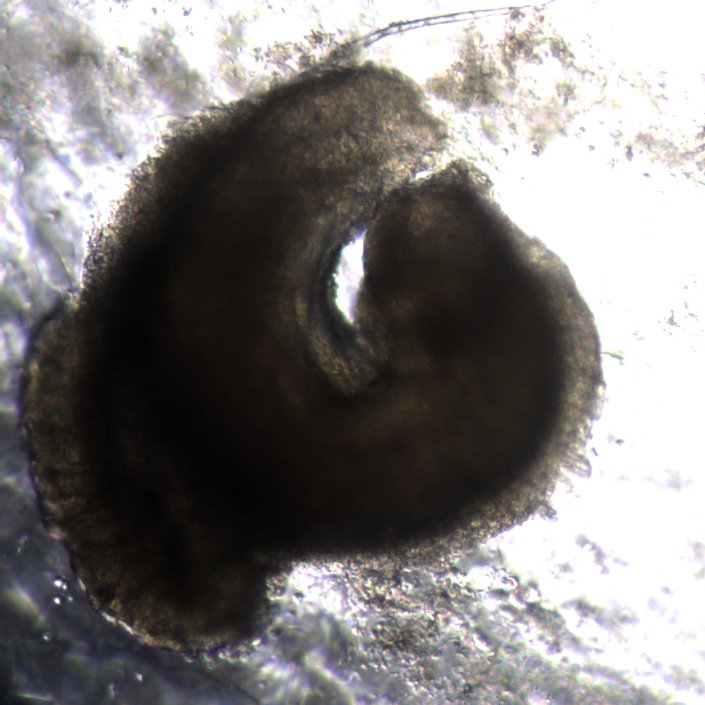

Supplement: Supplementary file 1 [file cells-11-00967-s001.zip › supplemetal videos/figure 3B mouse gut explant contraction JPEG time-lapse images/lobsterClaw495.jpg]

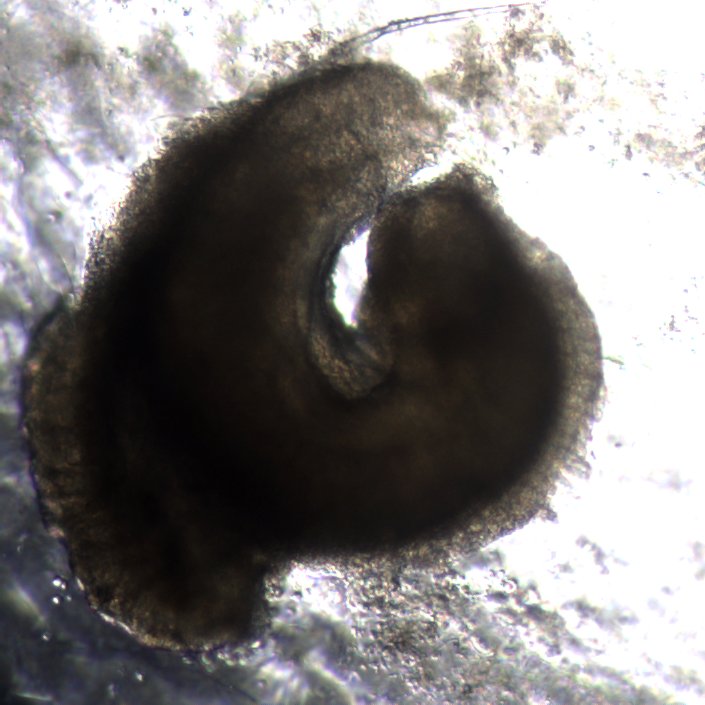

Supplement: Supplementary file 1 [file cells-11-00967-s001.zip › supplemetal videos/figure 3B mouse gut explant contraction JPEG time-lapse images/lobsterClaw481.jpg]

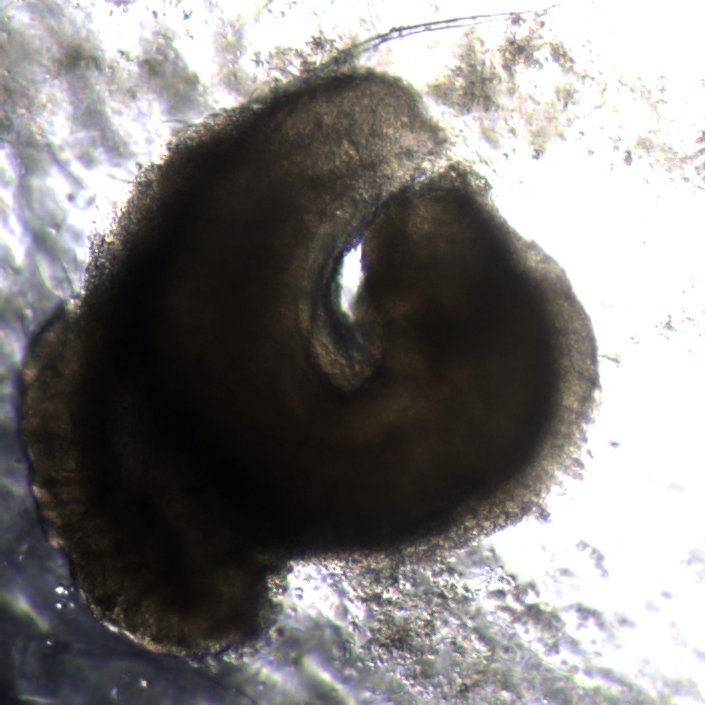

Supplement: Supplementary file 1 [file cells-11-00967-s001.zip › supplemetal videos/figure 3B mouse gut explant contraction JPEG time-lapse images/lobsterClaw132.jpg]

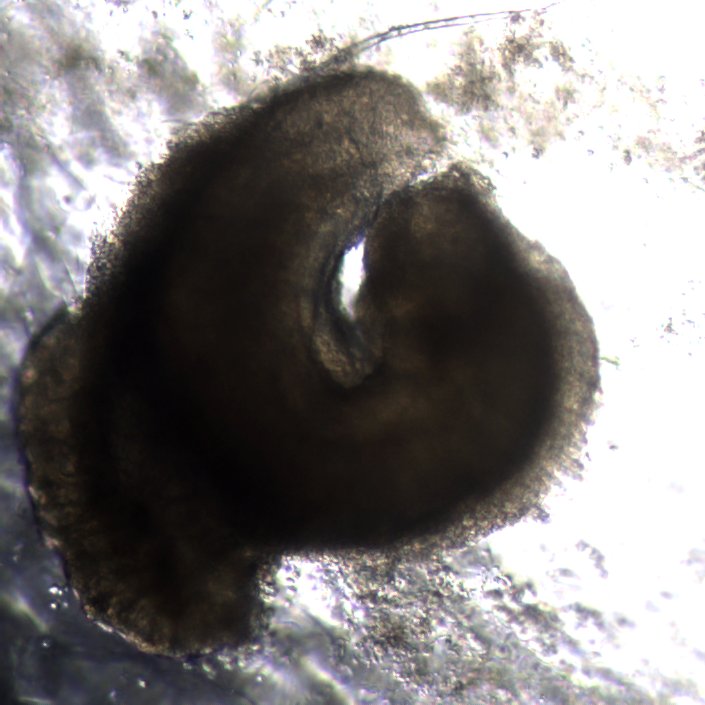

Supplement: Supplementary file 1 [file cells-11-00967-s001.zip › supplemetal videos/figure 3B mouse gut explant contraction JPEG time-lapse images/lobsterClaw126.jpg]

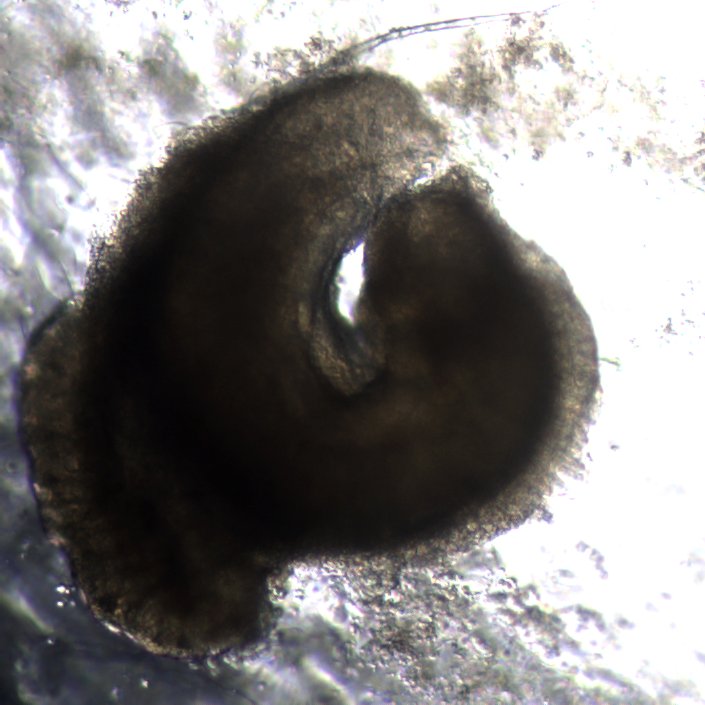

Supplement: Supplementary file 1 [file cells-11-00967-s001.zip › supplemetal videos/figure 3B mouse gut explant contraction JPEG time-lapse images/lobsterClaw046.jpg]

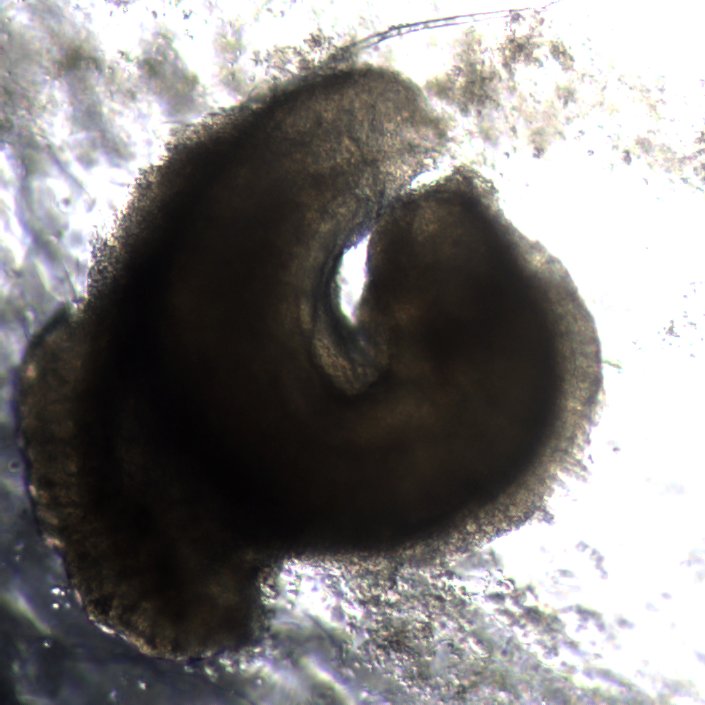

Supplement: Supplementary file 1 [file cells-11-00967-s001.zip › supplemetal videos/figure 3B mouse gut explant contraction JPEG time-lapse images/lobsterClaw052.jpg]

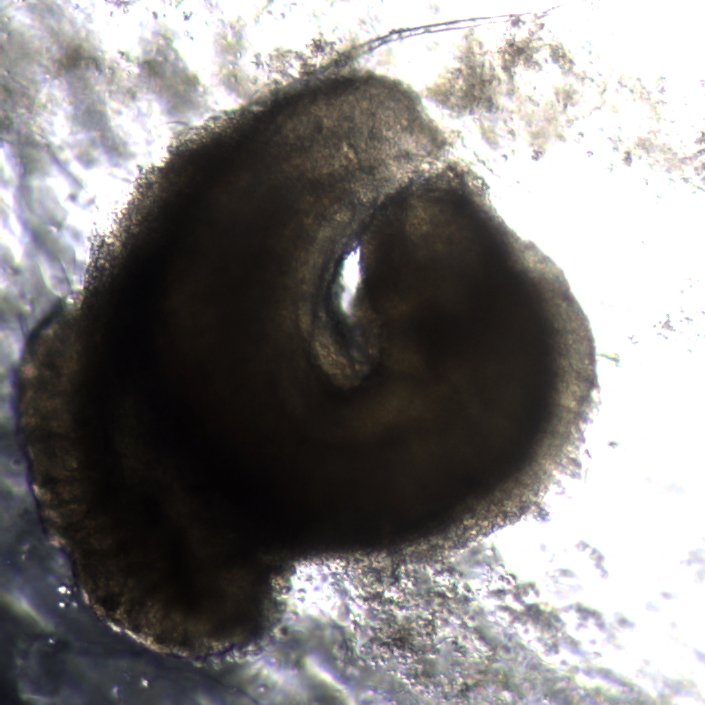

Supplement: Supplementary file 1 [file cells-11-00967-s001.zip › supplemetal videos/figure 3B mouse gut explant contraction JPEG time-lapse images/lobsterClaw085.jpg]

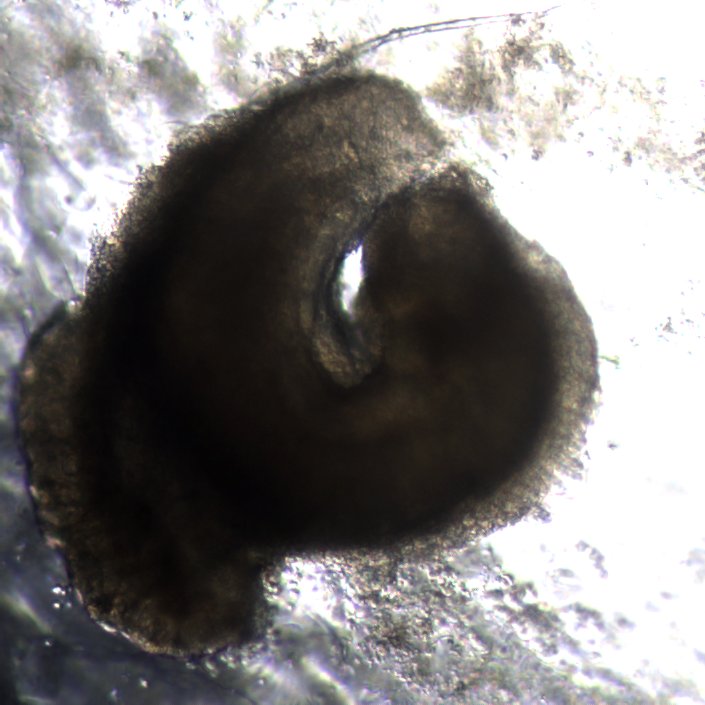

Supplement: Supplementary file 1 [file cells-11-00967-s001.zip › supplemetal videos/figure 3B mouse gut explant contraction JPEG time-lapse images/lobsterClaw091.jpg]

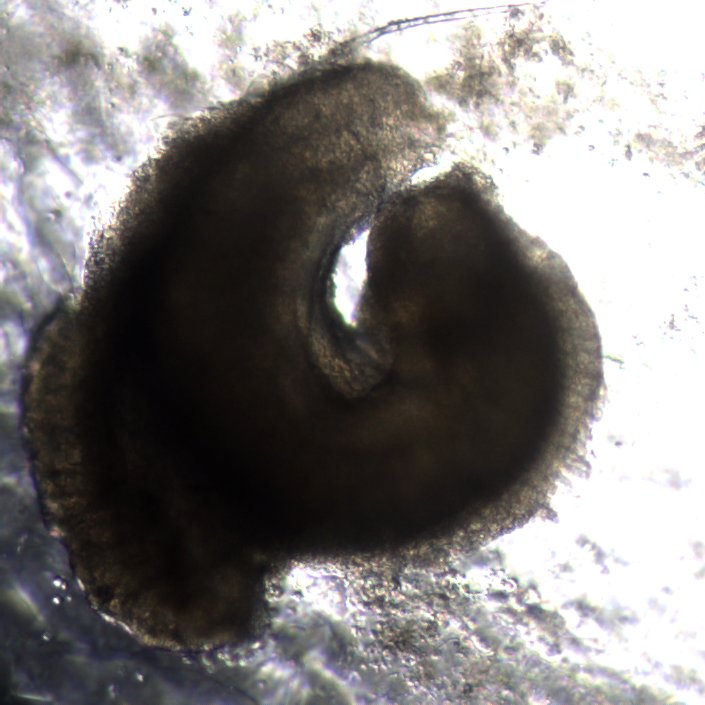

Supplement: Supplementary file 1 [file cells-11-00967-s001.zip › supplemetal videos/figure 3B mouse gut explant contraction JPEG time-lapse images/lobsterClaw522.jpg]

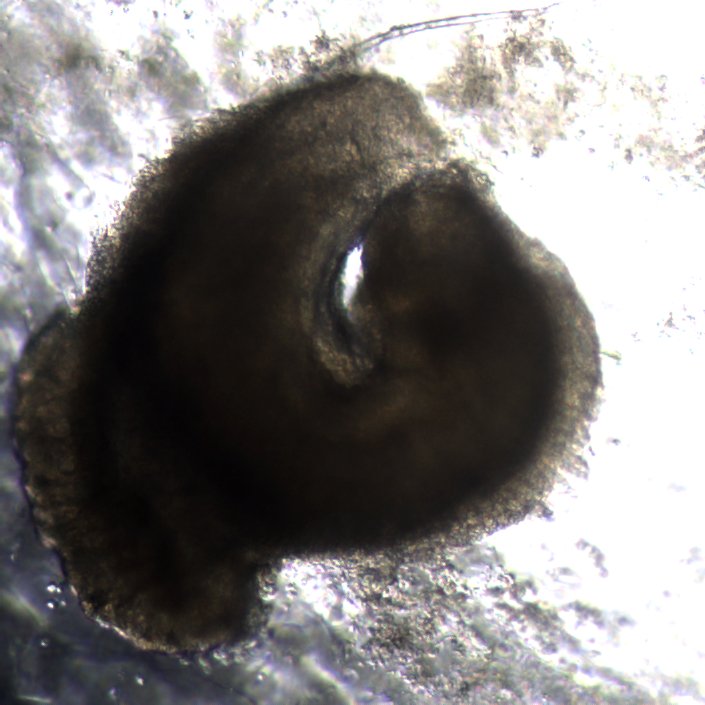

Supplement: Supplementary file 1 [file cells-11-00967-s001.zip › supplemetal videos/figure 3B mouse gut explant contraction JPEG time-lapse images/lobsterClaw244.jpg]

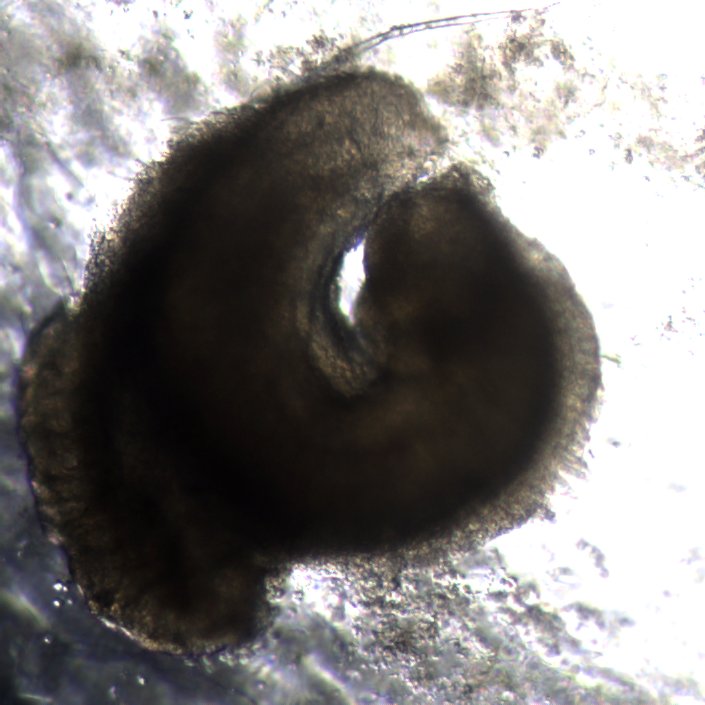

Supplement: Supplementary file 1 [file cells-11-00967-s001.zip › supplemetal videos/figure 3B mouse gut explant contraction JPEG time-lapse images/lobsterClaw250.jpg]

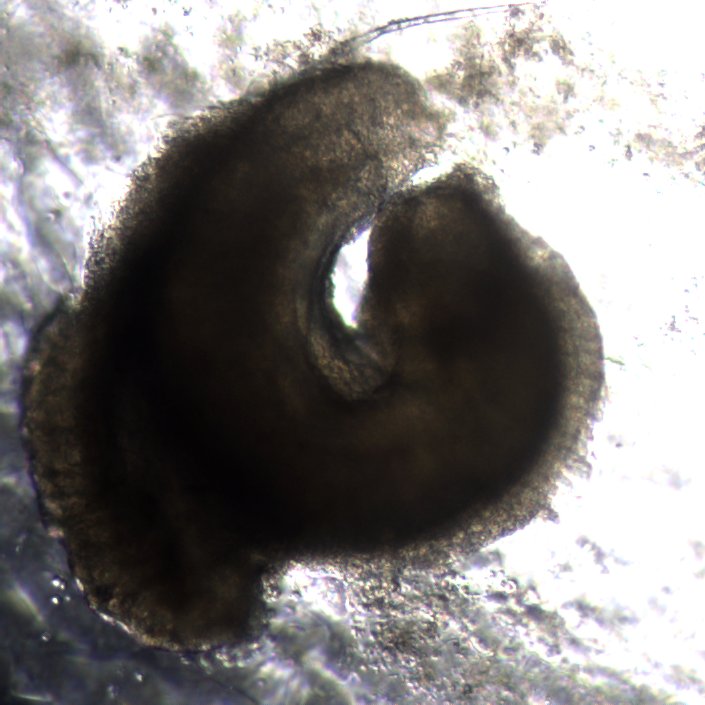

Supplement: Supplementary file 1 [file cells-11-00967-s001.zip › supplemetal videos/figure 3B mouse gut explant contraction JPEG time-lapse images/lobsterClaw536.jpg]

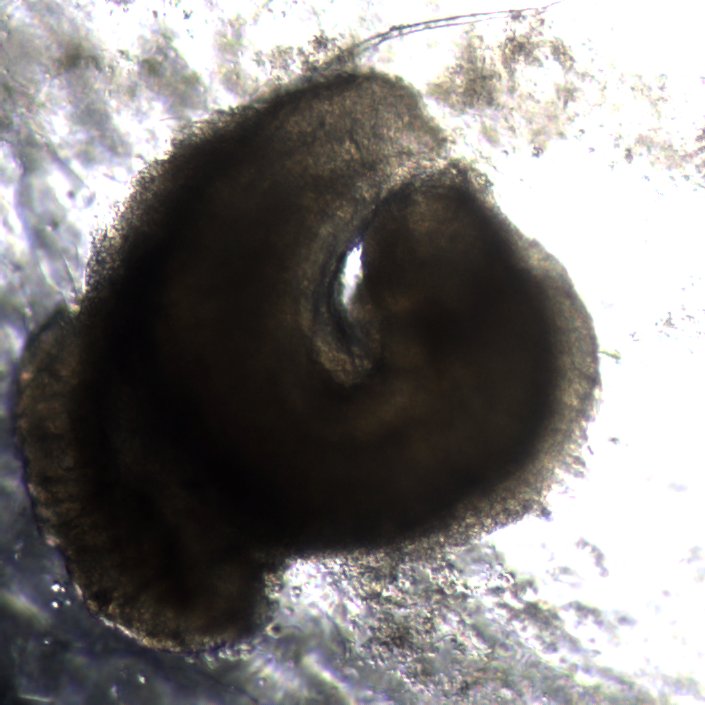

Supplement: Supplementary file 1 [file cells-11-00967-s001.zip › supplemetal videos/figure 3B mouse gut explant contraction JPEG time-lapse images/lobsterClaw278.jpg]

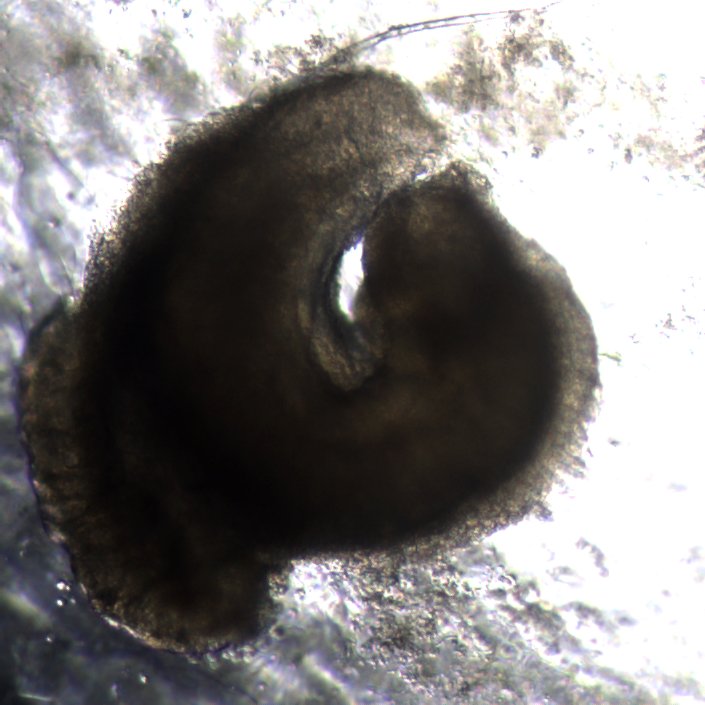

Supplement: Supplementary file 1 [file cells-11-00967-s001.zip › supplemetal videos/figure 3B mouse gut explant contraction JPEG time-lapse images/lobsterClaw287.jpg]

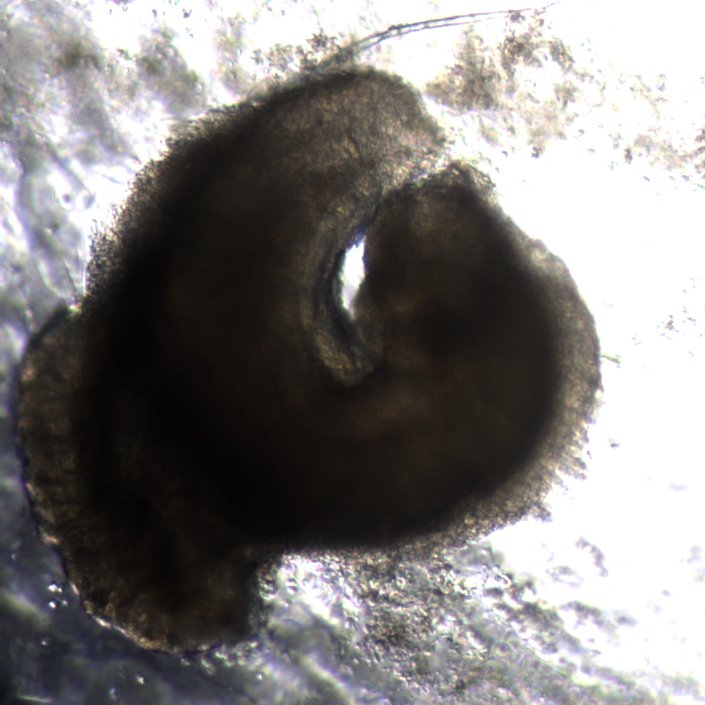

Supplement: Supplementary file 1 [file cells-11-00967-s001.zip › supplemetal videos/figure 3B mouse gut explant contraction JPEG time-lapse images/lobsterClaw293.jpg]

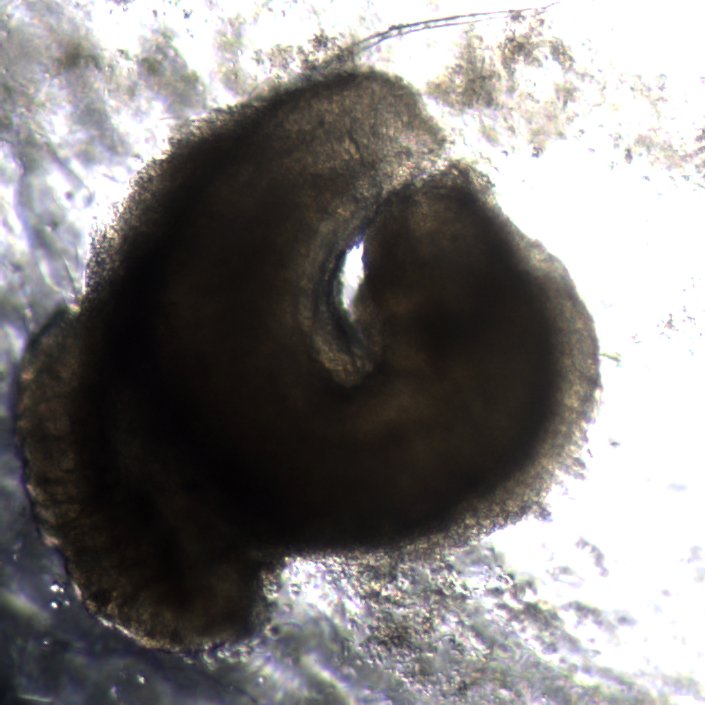

Supplement: Supplementary file 1 [file cells-11-00967-s001.zip › supplemetal videos/figure 3B mouse gut explant contraction JPEG time-lapse images/lobsterClaw292.jpg]

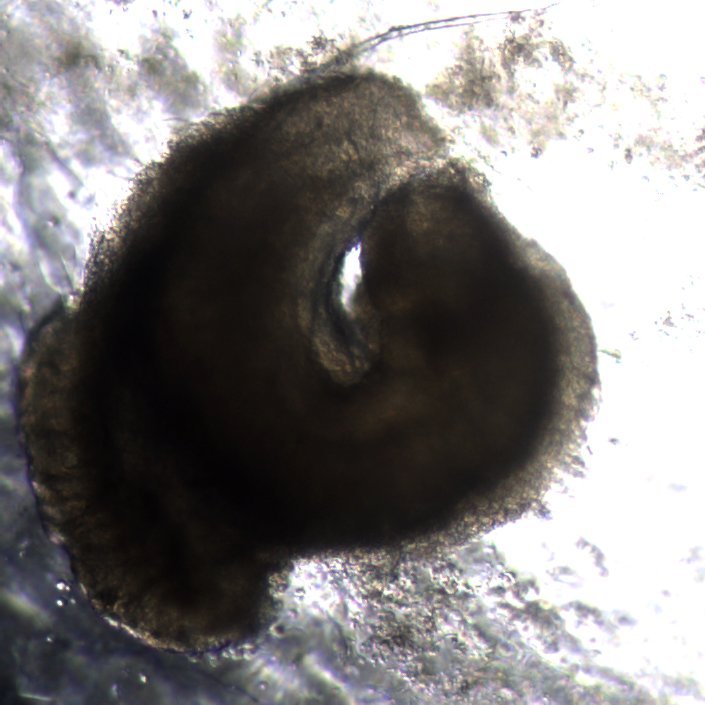

Supplement: Supplementary file 1 [file cells-11-00967-s001.zip › supplemetal videos/figure 3B mouse gut explant contraction JPEG time-lapse images/lobsterClaw286.jpg]

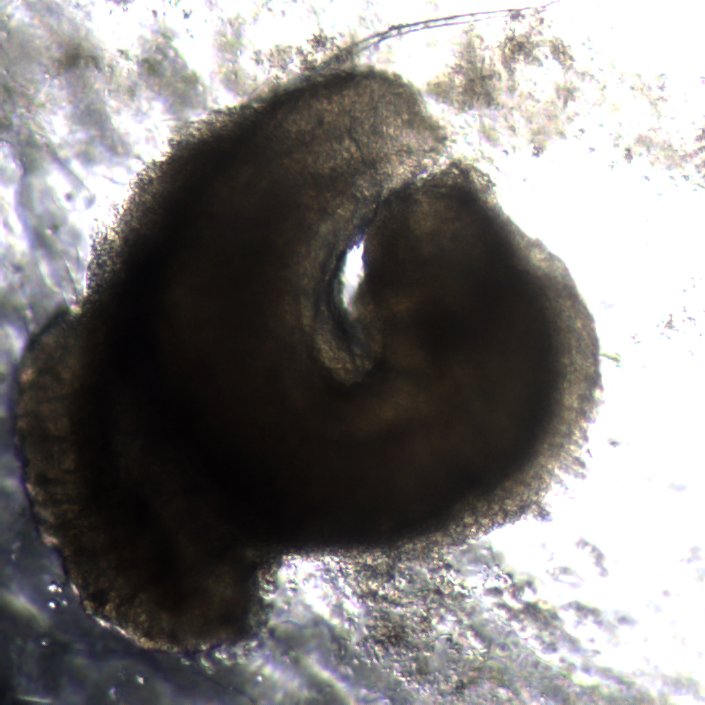

Supplement: Supplementary file 1 [file cells-11-00967-s001.zip › supplemetal videos/figure 3B mouse gut explant contraction JPEG time-lapse images/lobsterClaw279.jpg]

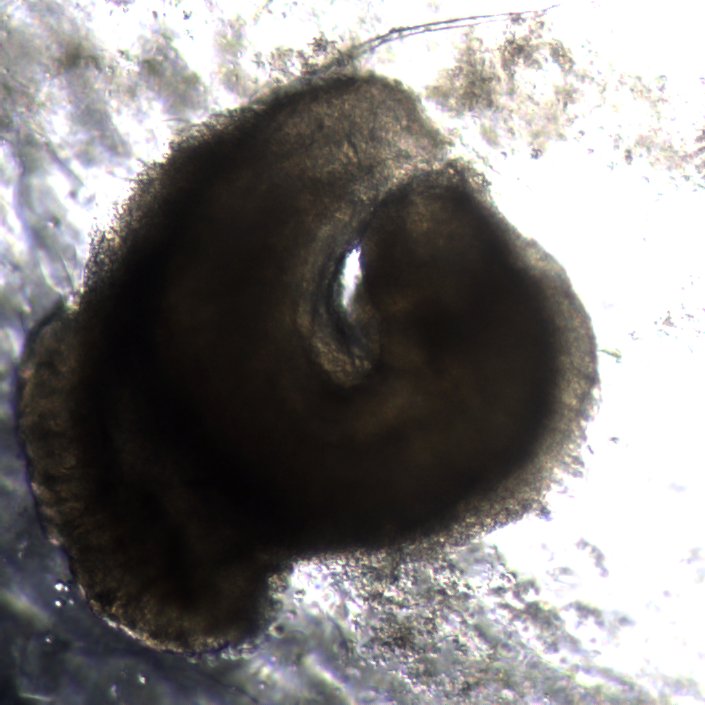

Supplement: Supplementary file 1 [file cells-11-00967-s001.zip › supplemetal videos/figure 3B mouse gut explant contraction JPEG time-lapse images/lobsterClaw251.jpg]

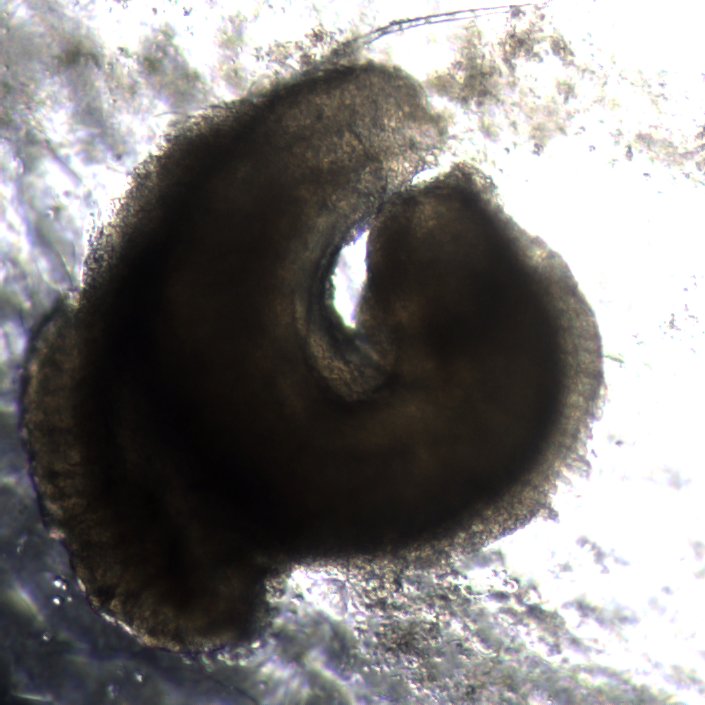

Supplement: Supplementary file 1 [file cells-11-00967-s001.zip › supplemetal videos/figure 3B mouse gut explant contraction JPEG time-lapse images/lobsterClaw537.jpg]

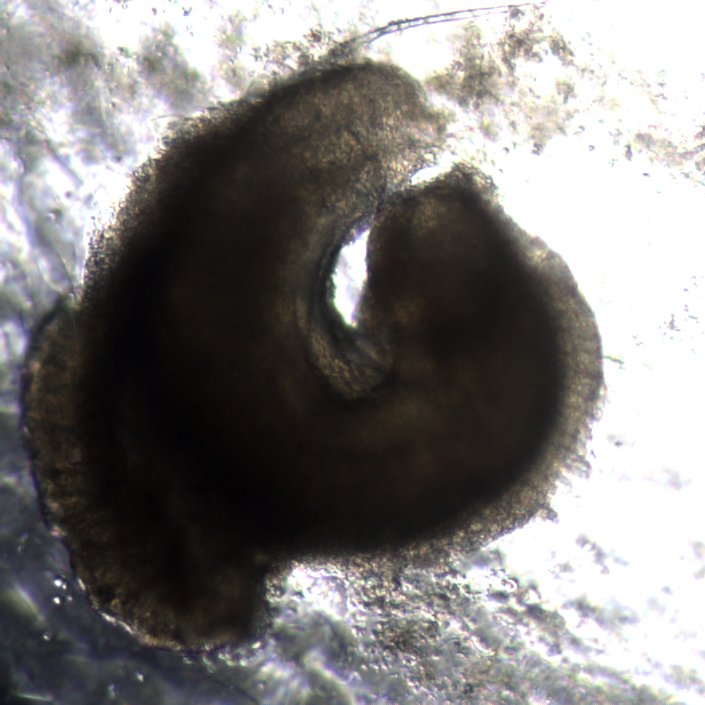

Supplement: Supplementary file 1 [file cells-11-00967-s001.zip › supplemetal videos/figure 3B mouse gut explant contraction JPEG time-lapse images/lobsterClaw523.jpg]

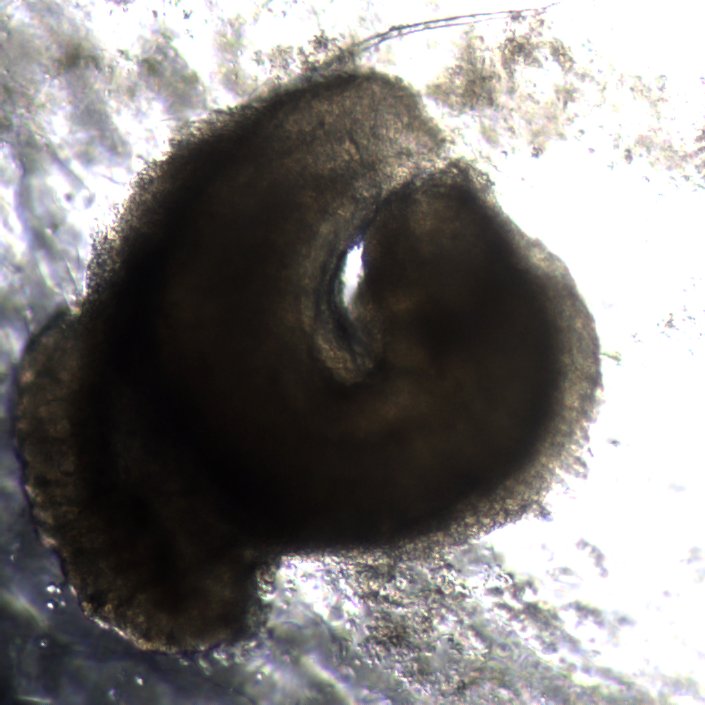

Supplement: Supplementary file 1 [file cells-11-00967-s001.zip › supplemetal videos/figure 3B mouse gut explant contraction JPEG time-lapse images/lobsterClaw245.jpg]

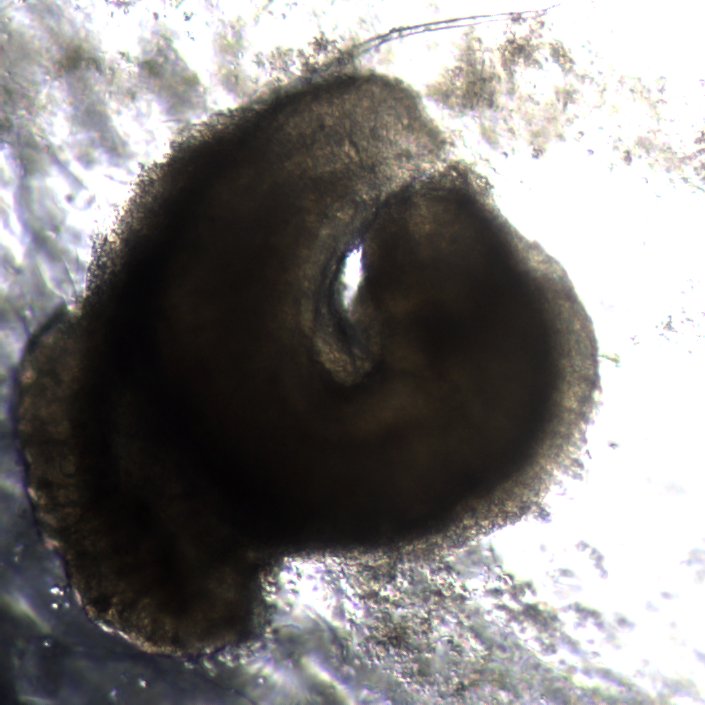

Supplement: Supplementary file 1 [file cells-11-00967-s001.zip › supplemetal videos/figure 3B mouse gut explant contraction JPEG time-lapse images/lobsterClaw090.jpg]

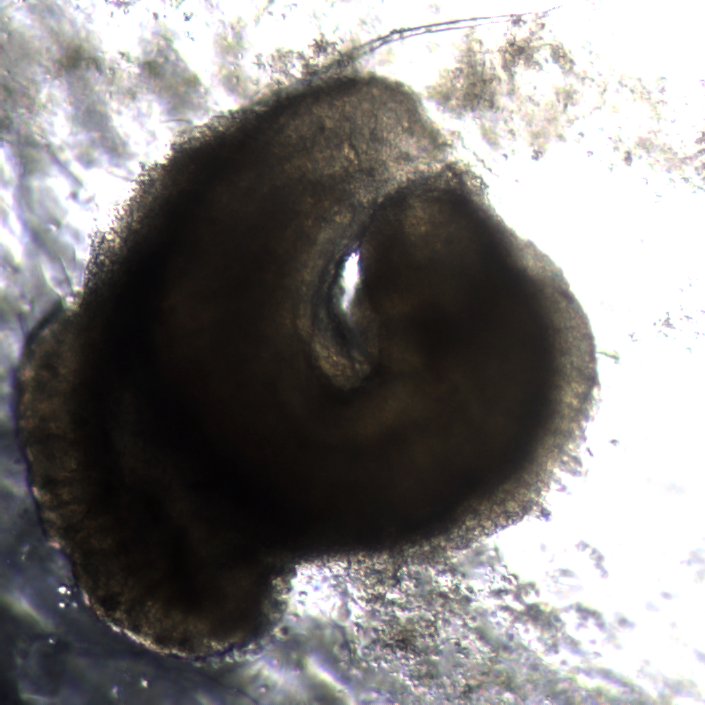

Supplement: Supplementary file 1 [file cells-11-00967-s001.zip › supplemetal videos/figure 3B mouse gut explant contraction JPEG time-lapse images/lobsterClaw084.jpg]

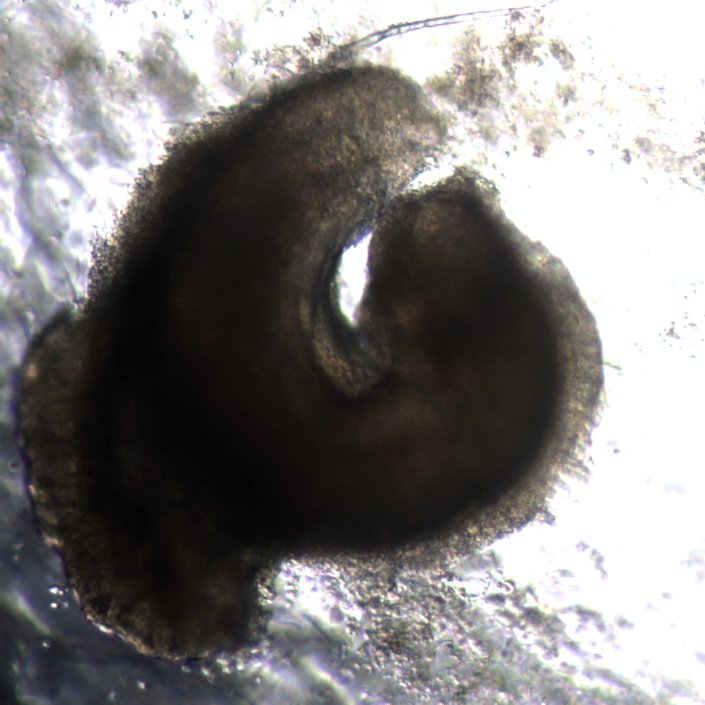

Supplement: Supplementary file 1 [file cells-11-00967-s001.zip › supplemetal videos/figure 3B mouse gut explant contraction JPEG time-lapse images/lobsterClaw053.jpg]

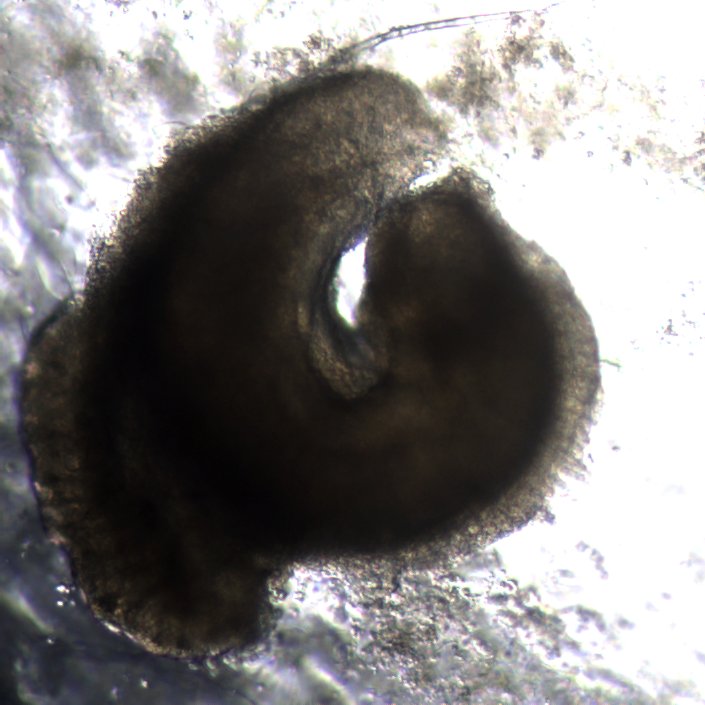

Supplement: Supplementary file 1 [file cells-11-00967-s001.zip › supplemetal videos/figure 3B mouse gut explant contraction JPEG time-lapse images/lobsterClaw047.jpg]

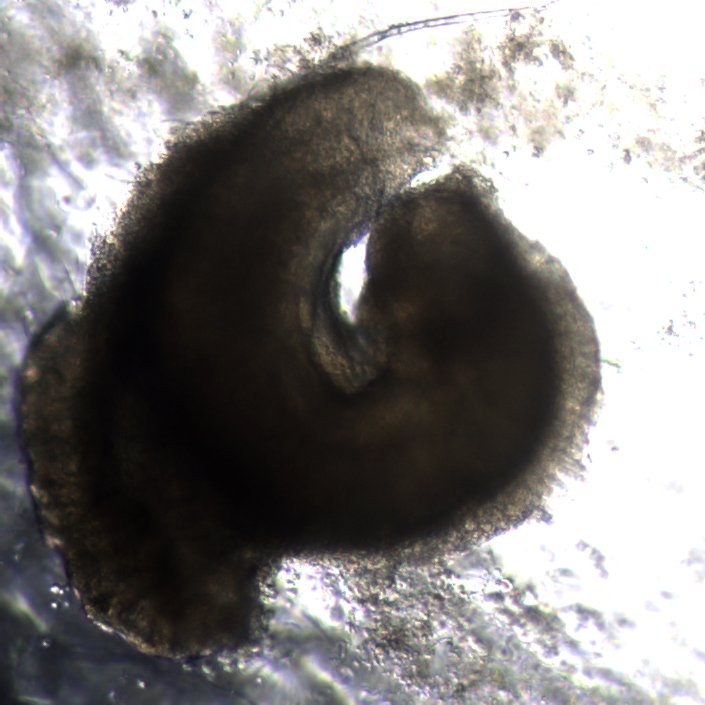

Supplement: Supplementary file 1 [file cells-11-00967-s001.zip › supplemetal videos/figure 3B mouse gut explant contraction JPEG time-lapse images/lobsterClaw127.jpg]

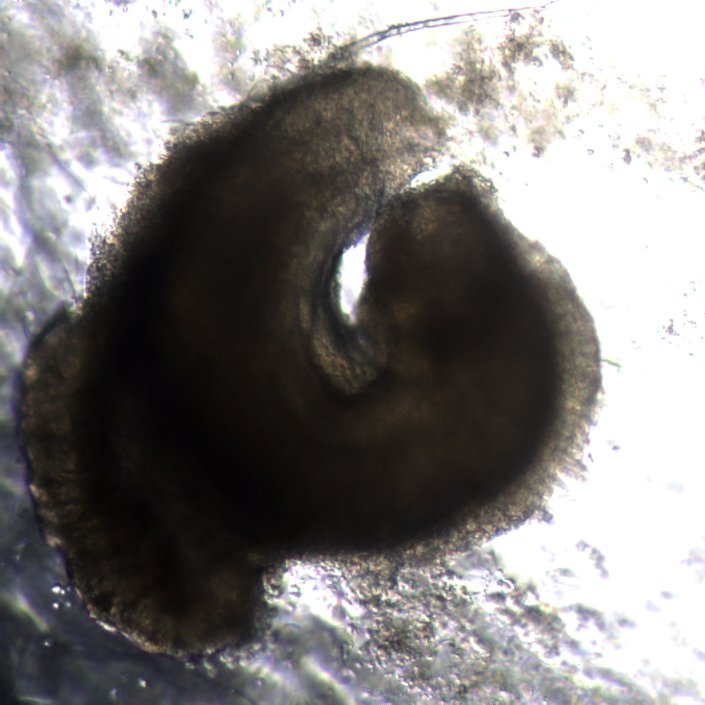

Supplement: Supplementary file 1 [file cells-11-00967-s001.zip › supplemetal videos/figure 3B mouse gut explant contraction JPEG time-lapse images/lobsterClaw133.jpg]

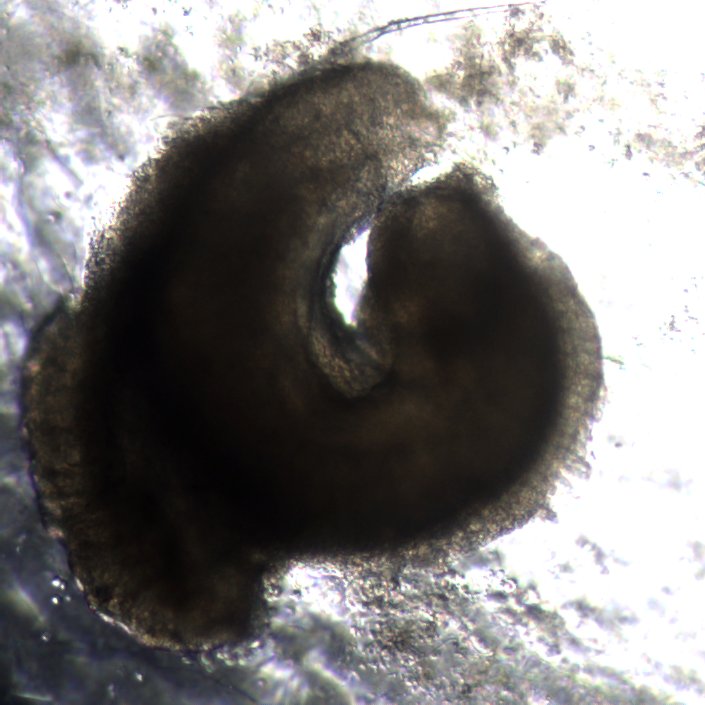

Supplement: Supplementary file 1 [file cells-11-00967-s001.zip › supplemetal videos/figure 3B mouse gut explant contraction JPEG time-lapse images/lobsterClaw480.jpg]

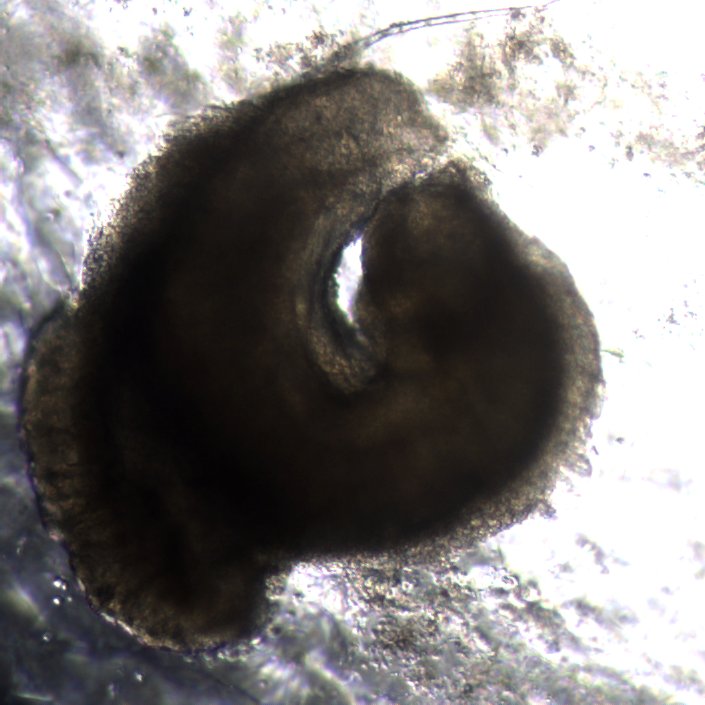

Supplement: Supplementary file 1 [file cells-11-00967-s001.zip › supplemetal videos/figure 3B mouse gut explant contraction JPEG time-lapse images/lobsterClaw494.jpg]

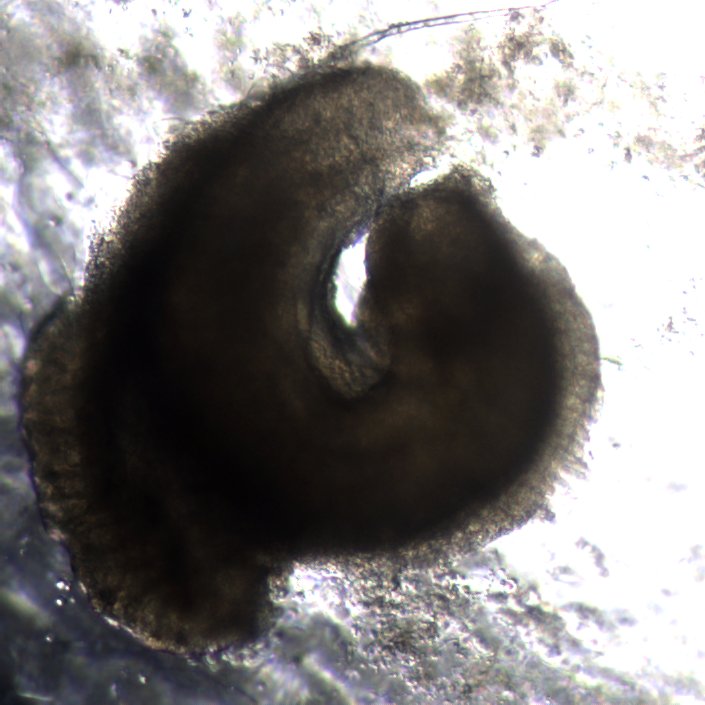

Supplement: Supplementary file 1 [file cells-11-00967-s001.zip › supplemetal videos/figure 3B mouse gut explant contraction JPEG time-lapse images/lobsterClaw319.jpg]

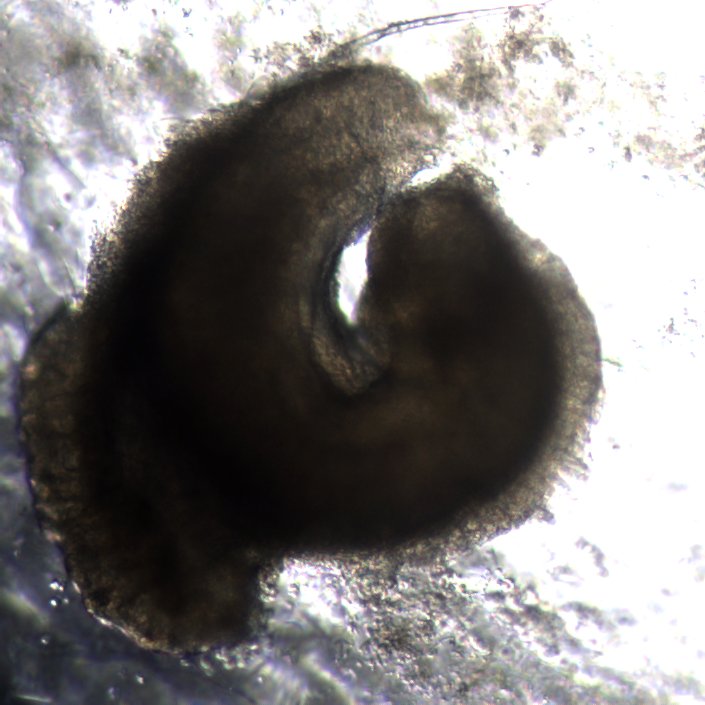

Supplement: Supplementary file 1 [file cells-11-00967-s001.zip › supplemetal videos/figure 3B mouse gut explant contraction JPEG time-lapse images/lobsterClaw325.jpg]

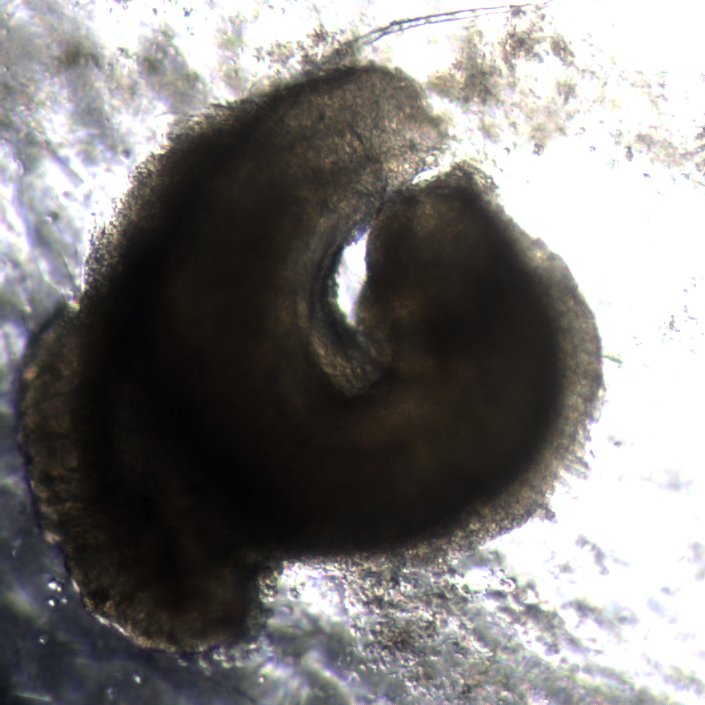

Supplement: Supplementary file 1 [file cells-11-00967-s001.zip › supplemetal videos/figure 3B mouse gut explant contraction JPEG time-lapse images/lobsterClaw443.jpg]

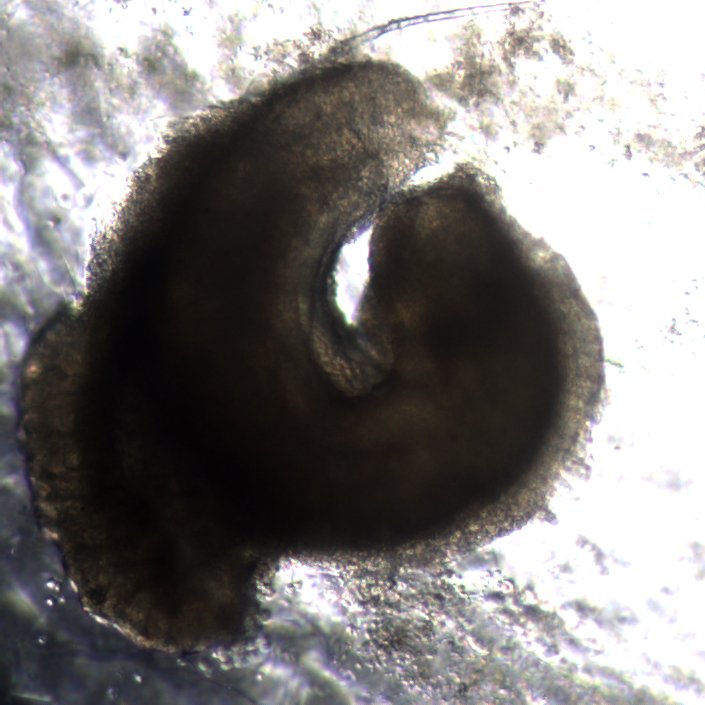

Supplement: Supplementary file 1 [file cells-11-00967-s001.zip › supplemetal videos/figure 3B mouse gut explant contraction JPEG time-lapse images/lobsterClaw457.jpg]

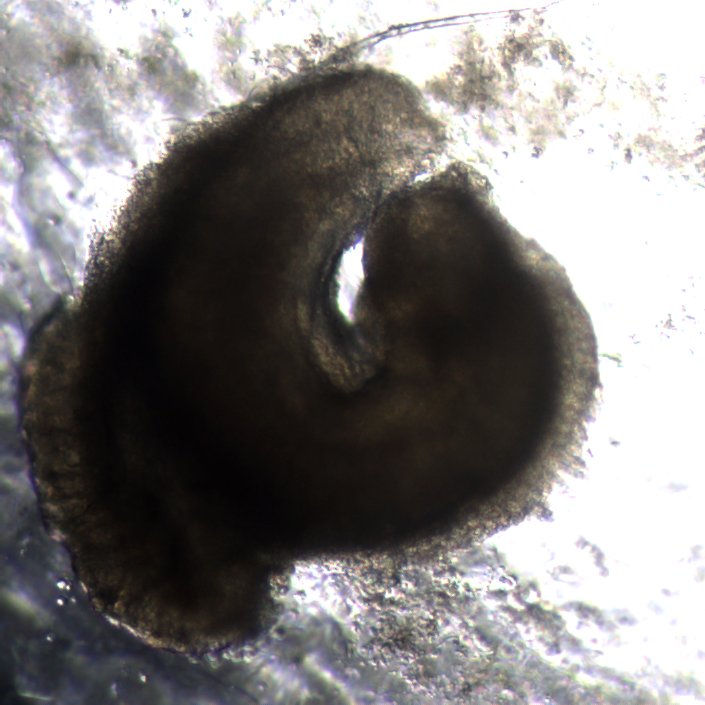

Supplement: Supplementary file 1 [file cells-11-00967-s001.zip › supplemetal videos/figure 3B mouse gut explant contraction JPEG time-lapse images/lobsterClaw331.jpg]

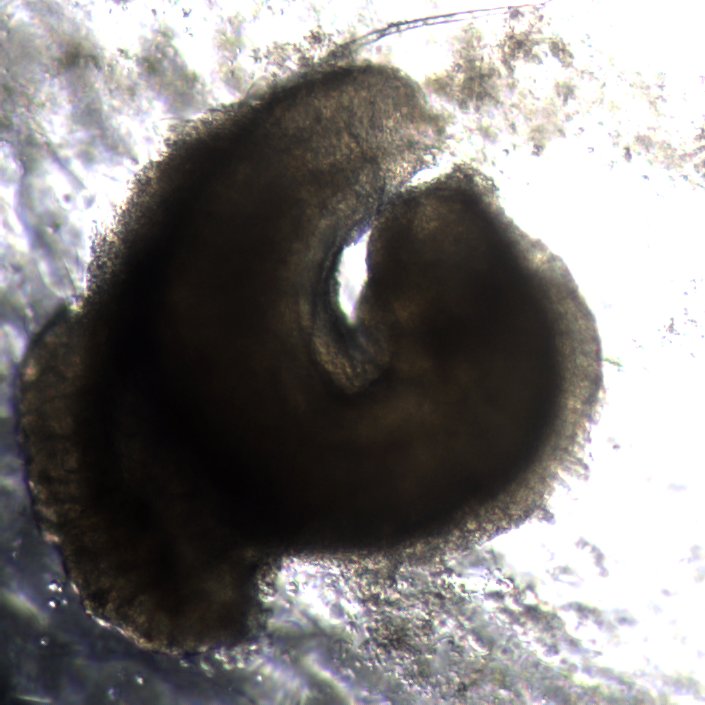

Supplement: Supplementary file 1 [file cells-11-00967-s001.zip › supplemetal videos/figure 3B mouse gut explant contraction JPEG time-lapse images/lobsterClaw309.jpg]

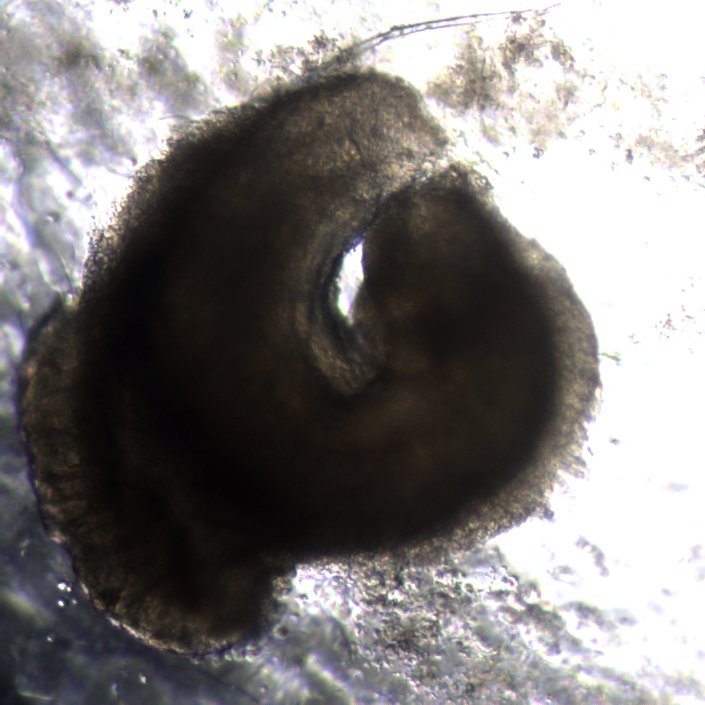

Supplement: Supplementary file 1 [file cells-11-00967-s001.zip › supplemetal videos/figure 3B mouse gut explant contraction JPEG time-lapse images/lobsterClaw335.jpg]

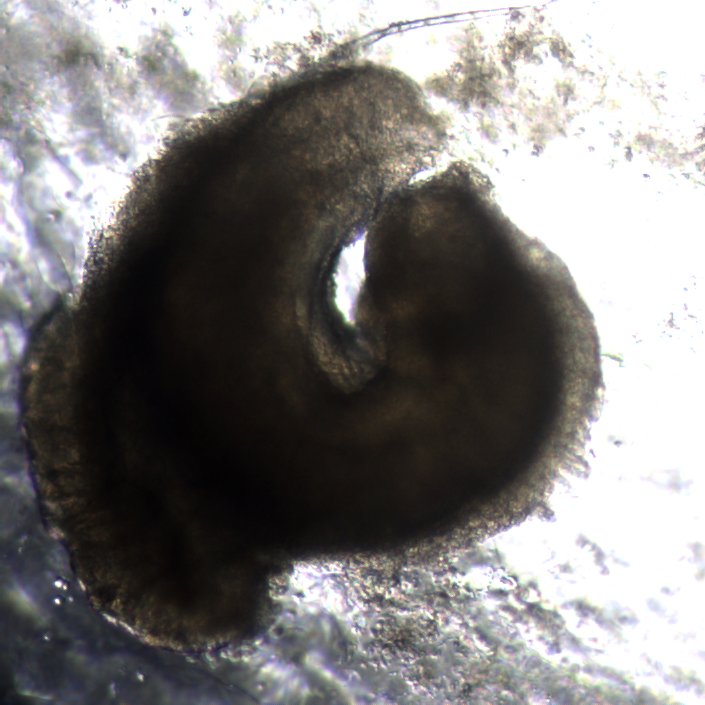

Supplement: Supplementary file 1 [file cells-11-00967-s001.zip › supplemetal videos/figure 3B mouse gut explant contraction JPEG time-lapse images/lobsterClaw453.jpg]

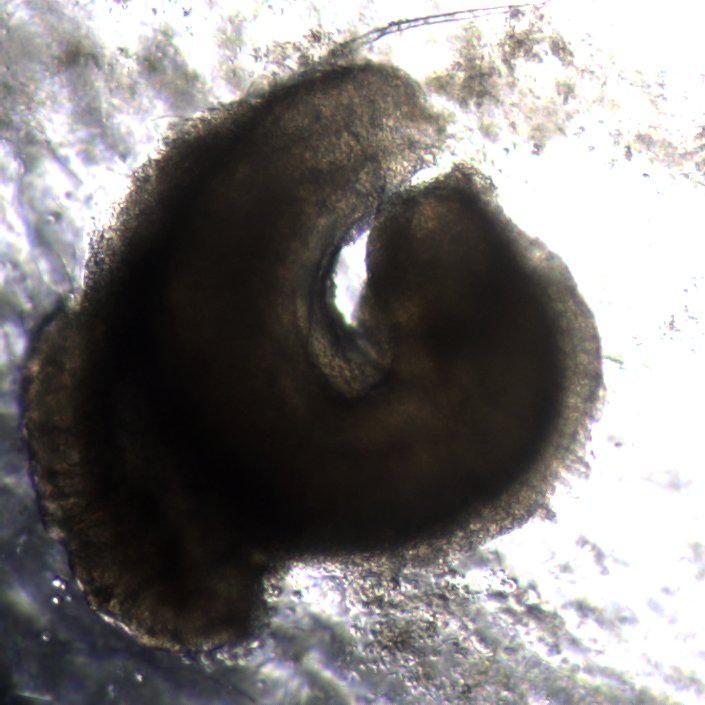

Supplement: Supplementary file 1 [file cells-11-00967-s001.zip › supplemetal videos/figure 3B mouse gut explant contraction JPEG time-lapse images/lobsterClaw447.jpg]

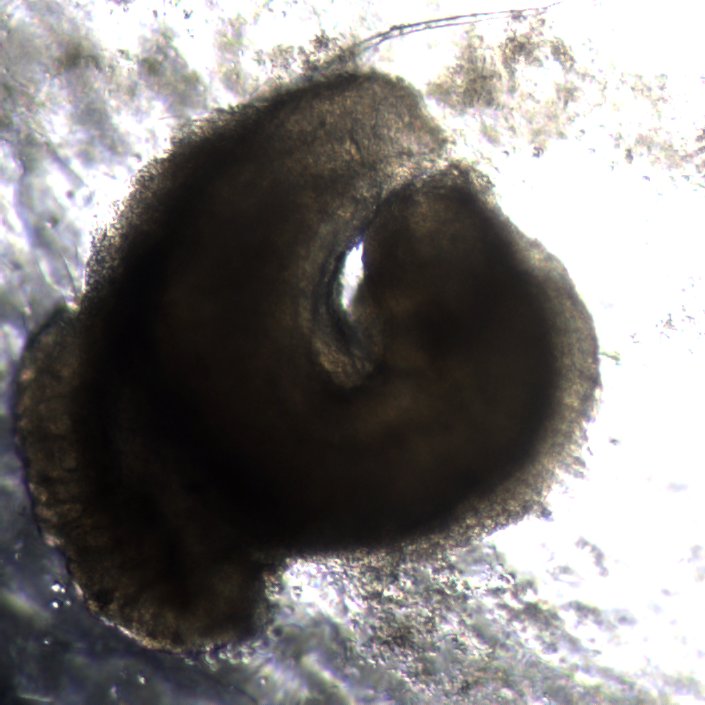

Supplement: Supplementary file 1 [file cells-11-00967-s001.zip › supplemetal videos/figure 3B mouse gut explant contraction JPEG time-lapse images/lobsterClaw321.jpg]

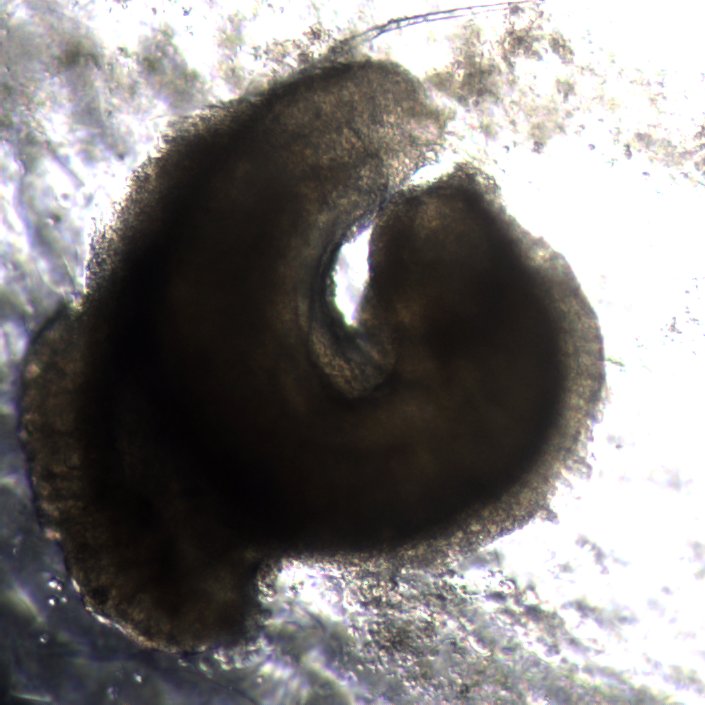

Supplement: Supplementary file 1 [file cells-11-00967-s001.zip › supplemetal videos/figure 3B mouse gut explant contraction JPEG time-lapse images/lobsterClaw490.jpg]

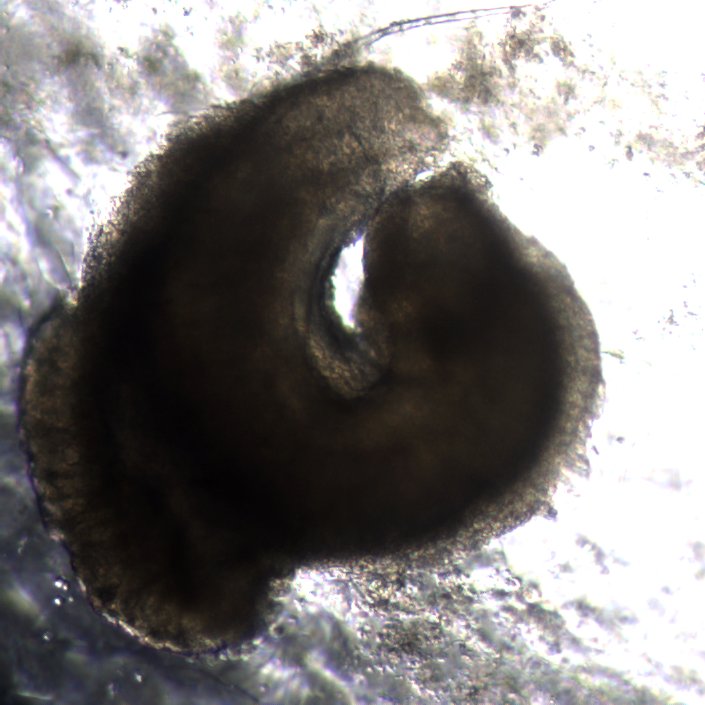

Supplement: Supplementary file 1 [file cells-11-00967-s001.zip › supplemetal videos/figure 3B mouse gut explant contraction JPEG time-lapse images/lobsterClaw484.jpg]

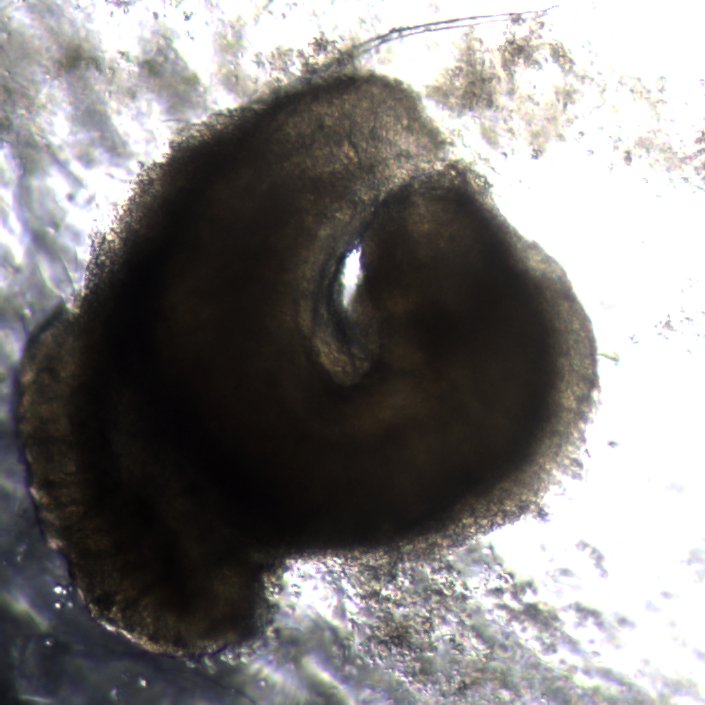

Supplement: Supplementary file 1 [file cells-11-00967-s001.zip › supplemetal videos/figure 3B mouse gut explant contraction JPEG time-lapse images/lobsterClaw137.jpg]

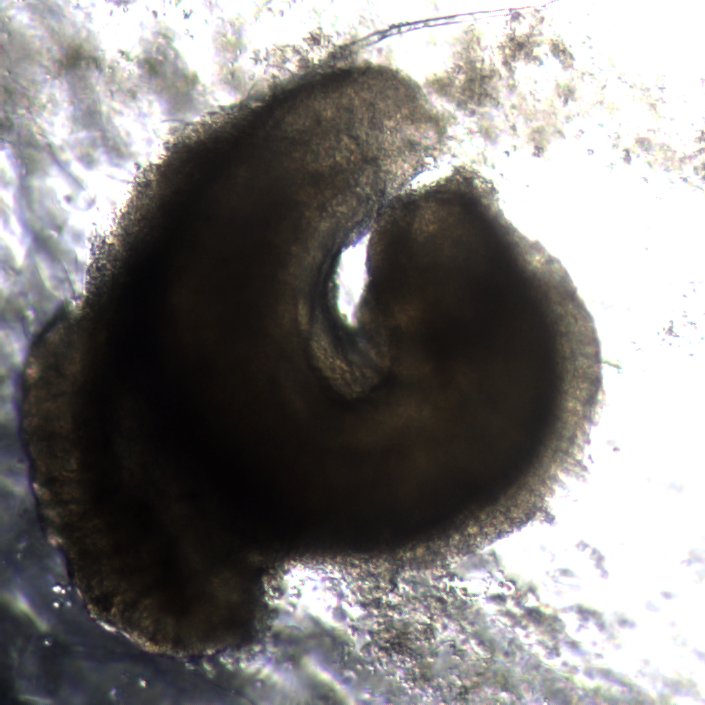

Supplement: Supplementary file 1 [file cells-11-00967-s001.zip › supplemetal videos/figure 3B mouse gut explant contraction JPEG time-lapse images/lobsterClaw123.jpg]

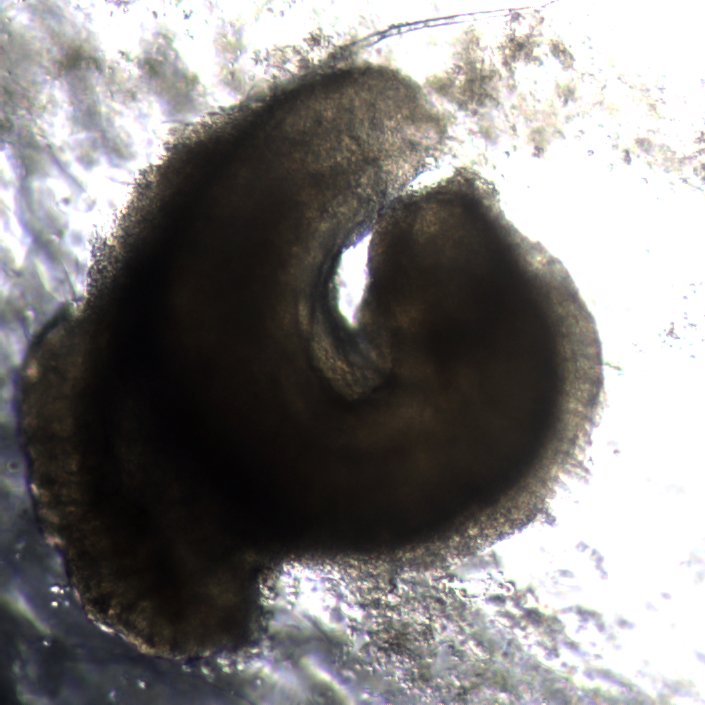

Supplement: Supplementary file 1 [file cells-11-00967-s001.zip › supplemetal videos/figure 3B mouse gut explant contraction JPEG time-lapse images/lobsterClaw043.jpg]

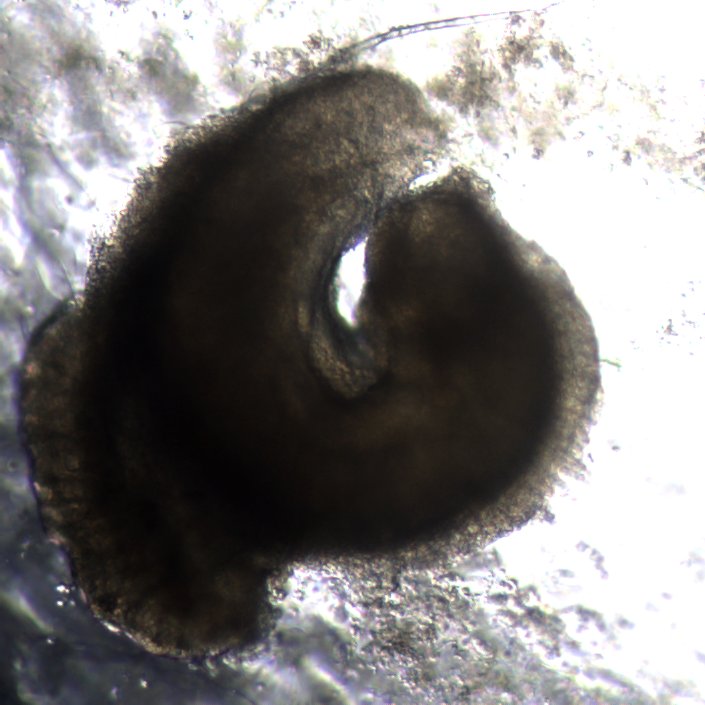

Supplement: Supplementary file 1 [file cells-11-00967-s001.zip › supplemetal videos/figure 3B mouse gut explant contraction JPEG time-lapse images/lobsterClaw057.jpg]

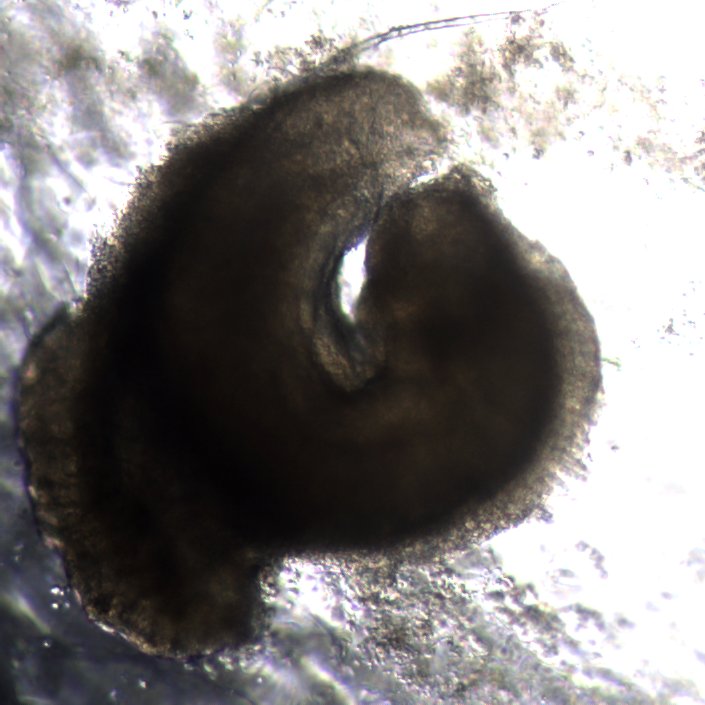

Supplement: Supplementary file 1 [file cells-11-00967-s001.zip › supplemetal videos/figure 3B mouse gut explant contraction JPEG time-lapse images/lobsterClaw080.jpg]

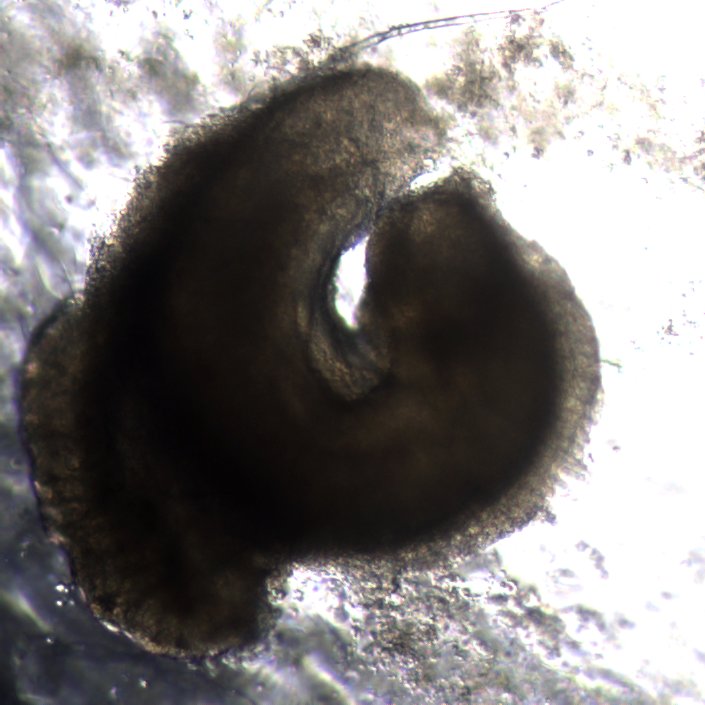

Supplement: Supplementary file 1 [file cells-11-00967-s001.zip › supplemetal videos/figure 3B mouse gut explant contraction JPEG time-lapse images/lobsterClaw094.jpg]

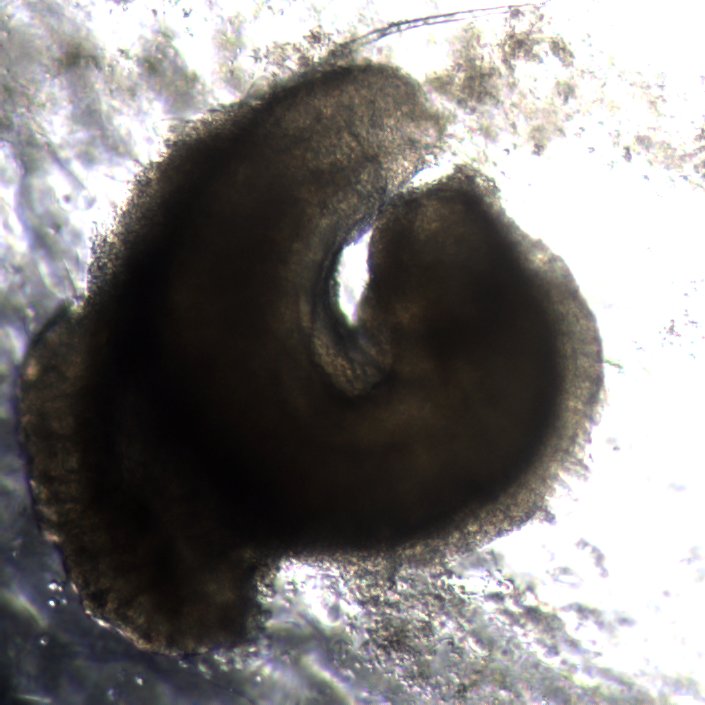

Supplement: Supplementary file 1 [file cells-11-00967-s001.zip › supplemetal videos/figure 3B mouse gut explant contraction JPEG time-lapse images/lobsterClaw269.jpg]

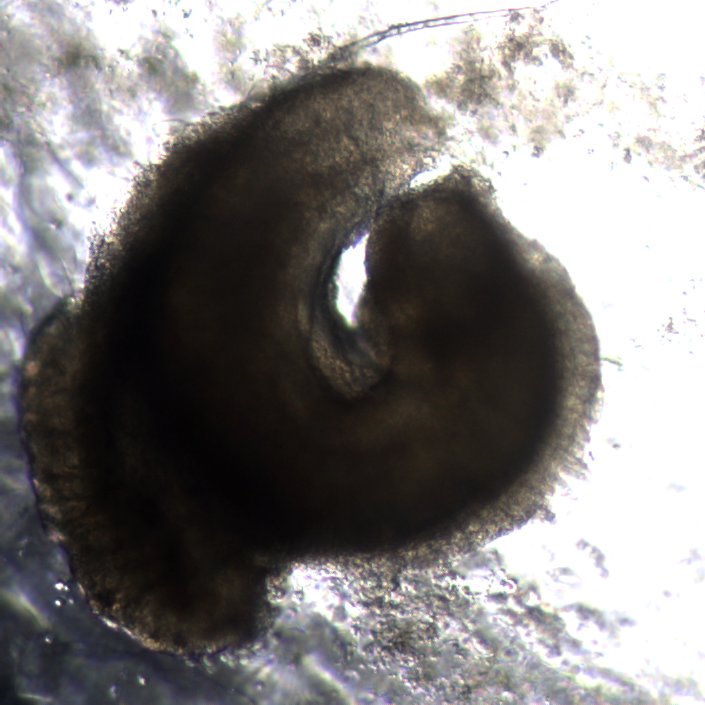

Supplement: Supplementary file 1 [file cells-11-00967-s001.zip › supplemetal videos/figure 3B mouse gut explant contraction JPEG time-lapse images/lobsterClaw241.jpg]

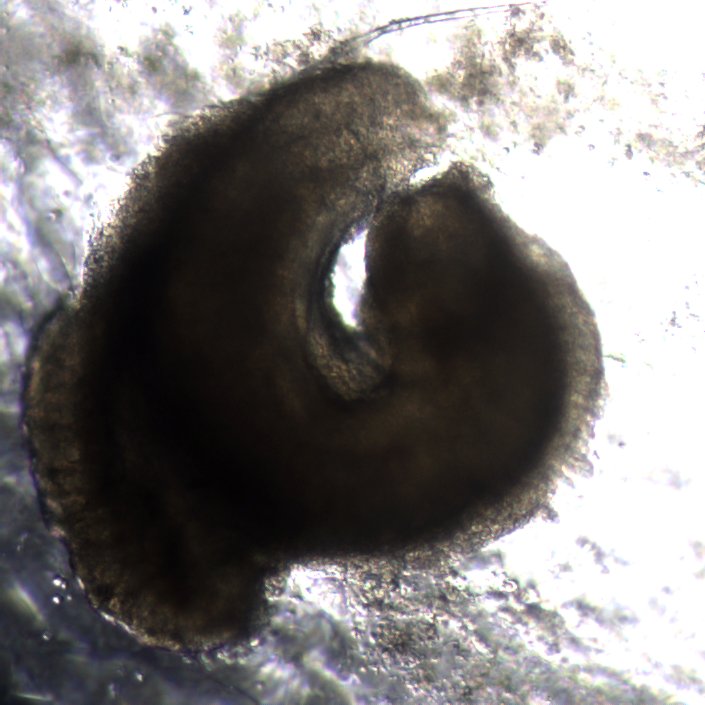

Supplement: Supplementary file 1 [file cells-11-00967-s001.zip › supplemetal videos/figure 3B mouse gut explant contraction JPEG time-lapse images/lobsterClaw527.jpg]
